# Quantitative modeling of the impact of facemasks and associated leakage on the airborne transmission of SARS-CoV-2

**DOI:** 10.1038/s41598-021-98895-9

**Published:** 2021-09-30

**Authors:** Jean Schmitt, Jing Wang

**Affiliations:** 1 Institute of Environmental Engineering, Department of Civil, Environmental and Geomatic Engineering, ETH Zurich, 8093 Zurich, Switzerland; 2 Laboratory for Advanced Analytical Technologies, Empa, Swiss Federal Laboratories for Materials Science and Technology, 8600 Dubendorf, Switzerland

**Keywords:** Engineering, Mechanical engineering, Environmental sciences

## Abstract

The ongoing worldwide outbreak of COVID-19 has set personal protective equipment in the spotlight. A significant number of countries impose the use of facemasks in public spaces and encourage it in the private sphere. Even in countries where relatively high vaccination rates are achieved at present, breakthrough infections have been frequently reported and usage of facemasks in certain settings has been recommended again. Alternative solutions, including community masks fabricated using various materials, such as cotton or jersey, have emerged alongside facemasks following long-established standards (e.g., EN 149, EN 14683). In the present work, we present a computational model to calculate the ability of different types of facemasks to reduce the exposure to virus-laden respiratory particles, with a focus on the relative importance of the filtration properties and the fitting on the wearer’s face. The model considers the facemask and the associated leakage, the transport of respiratory particles and their accumulation around the emitter, as well as the fraction of the inhaled particles deposited in the respiratory system. Different levels of leakages are considered to represent the diversity of fittings likely to be found among a population of non-trained users. The leakage prevails over the filtration performance of a facemask in determining the exposure level, and the ability of a face protection to limit leakages needs to be taken into account to accurately estimate the provided protection. Filtering facepieces (FFP) provide a better protection efficiency than surgical and community masks due to their higher filtration efficiency and their ability to provide a better fit and thus reduce the leakages. However, an improperly-fitted FFP mask loses a critical fraction of its protection efficiency, which may drop below the protection level provided by properly-worn surgical and community masks.

## Introduction

The worldwide outbreak of COVID-19 leads to a surge in demand for facemasks and a rising pressure on the supply chains^1^. Even in countries where relatively high vaccination rates are achieved at present, breakthrough infections have been frequently reported and usage of facemasks in certain settings has been recommended again^2–4^. Alternative face protections, including face-shields and homemade masks, have emerged to overcome the shortages in facemasks certified according to long-established standards (EN 149, EN 14683, NIOSH42-CFR84, and other equivalents). To regulate the protection efficiency of emerging face protections, new standards have been defined such as the SNR 30000:2021 in Switzerland or the CWA 17553:2020 in Europe.

There is evidence toward the airborne transmission of SARS-CoV-2^5–8^. Copies of SARS-CoV-2 have been found in respiratory particles emitted by infected individuals^9^ and in aerosol samples from hospital rooms^10^. The Centers for Disease Control and Prevention (CDC) describe three transmission routes for respiratory diseases^11^: contact transmission involving touching infected individuals or contaminated surfaces, droplet transmission through emitted respiratory particles that spread over a short distance from the infected individual, and aerosol transmission by smaller particles which have a long residence time in the air and can spread over longer distances.

The efficacy of facemasks in reducing the spread of respiratory viruses is supported by several epidemiological studies: a meta-analysis^12^ covering SARS-CoV, H1N1, SARS-CoV-2, and influenza estimated the reduction of the infection risk from wearing masks by 80% among healthcare workers and by 47% among non-healthcare workers. An investigation of different protection measures against Covid-19^13^ concluded that facemasks might lead to a reduction of the infection risk, with N95 providing a higher protection than surgical masks. Measurements performed on individuals infected with several respiratory viruses^9^ indicated that surgical masks used as source control significantly reduce the emission of influenza virus carried by particles larger than 5 μm.

Computational fluid dynamics (CFD) has been extensively used to investigate the spread of SARS-CoV-2 in different situations: models have been developed to simulate the spread of an aerosol cloud in a supermarket^14^ and in a restaurant including an estimation of the infection risk considering different environmental parameters^15–17^. On another scale, CFD has been applied to analyze the interaction between the emitted particles and facemasks^18^, to investigate the flow within the volume between the wearer’s face and a facemask^19^, and to compare the transport of respiratory droplets generated by various expiratory events^20^ and assess the influence of a facemask^21^. A GRAMM/GRAL atmospheric dispersion model has been applied to investigate the spread of SARS-CoV-2 in a seafood market^22^, including a 1-compartment model used to calculate the accumulation of viral charges in a closed environment^23^. Epidemiological models, SEIR (Susceptible, Exposed, Infected, Recovered)^24^ and SIR (Susceptible, Infected, Recovered)^25^, have been used to assess the efficiency of facemasks in reducing the death rate and the infection risk. A simulator has been developed to estimate the indoor infection risk in different scenarios^26^.

The development of the Droplet Nuclei Theory^27^ is the baseline for modeling frameworks used by the CDC and the World Health Organization (WHO) to set their physical distancing guidelines^28,29^. A physical model has been later developed on the basis of the Wells evaporation-falling curve to investigate the influence of humidity, air velocity, and respiratory jets on the spread of respiratory droplets^30^. A turbulent jet model^31^ has been used to compute the trajectories of expiratory droplets^32^. A general framework has been proposed to investigate the airborne transmission of COVID-19^33^, and models have been developed to estimate the risk of airborne infection, combining empirical distributions of emitted particles, modeling of particles transport and evaporation, and calculation of the inhalation of viral charges^34,35^. Even though the above mentioned models rely on simplified or empirical equations instead of full-scale CFD simulation, they have found wide applications to estimate the influence of distance and face protections on the infection risk.

The aim of the present work is to provide a model for quantitative calculation of the reduction of virus transmission through mask usage with focus on the impact of different levels of leakages to reflect realistic usage of facemasks within a non-trained population. The framework proposes a dynamic calculation of the masks’ filtration characteristics as a function of the changing flow velocities, as well as a model to estimate the fraction of particles traveling through the leakages. Such a framework can help improving the design of masks, as recent studies showed that their efficiency greatly benefited from adaptations to the wide diversity of face geometries within the population^36^. The integral model consists of a suite of detailed modules describing the processes from virus emission, blocking by the emitter’s facemask, transport and accumulation of the respiratory particles in air, protection by the receiver’s facemask, to particle deposition in the receiver’s lungs. The size distributions of particles carrying viral charges were estimated from data available in the literature, as well as the filtration efficiencies of several types of facemasks. The leaking flow was calculated according to various scenarios considering optimistic, realistic, and degraded fitting conditions taking into account the differences in pressure drops between the masks. The exposure level was based on the trajectories and accumulation of particles around the emitter, combined with the NCRP (National Council on Radiation Protection and Measurements) lung deposition model.

The modeling results are presented in the following order. The spatial spread of viral charges, illustrated through the example of a cough, and the containment through the use of a facemask are shown first, with a focus on the sizes of the emitted particles. Then the influence of the filtration properties of various types of masks on the exposure to viral charges is presented. Finally, the influence of leakage on the protection efficiency is simulated.

## Results and discussion

**Figure 1:**
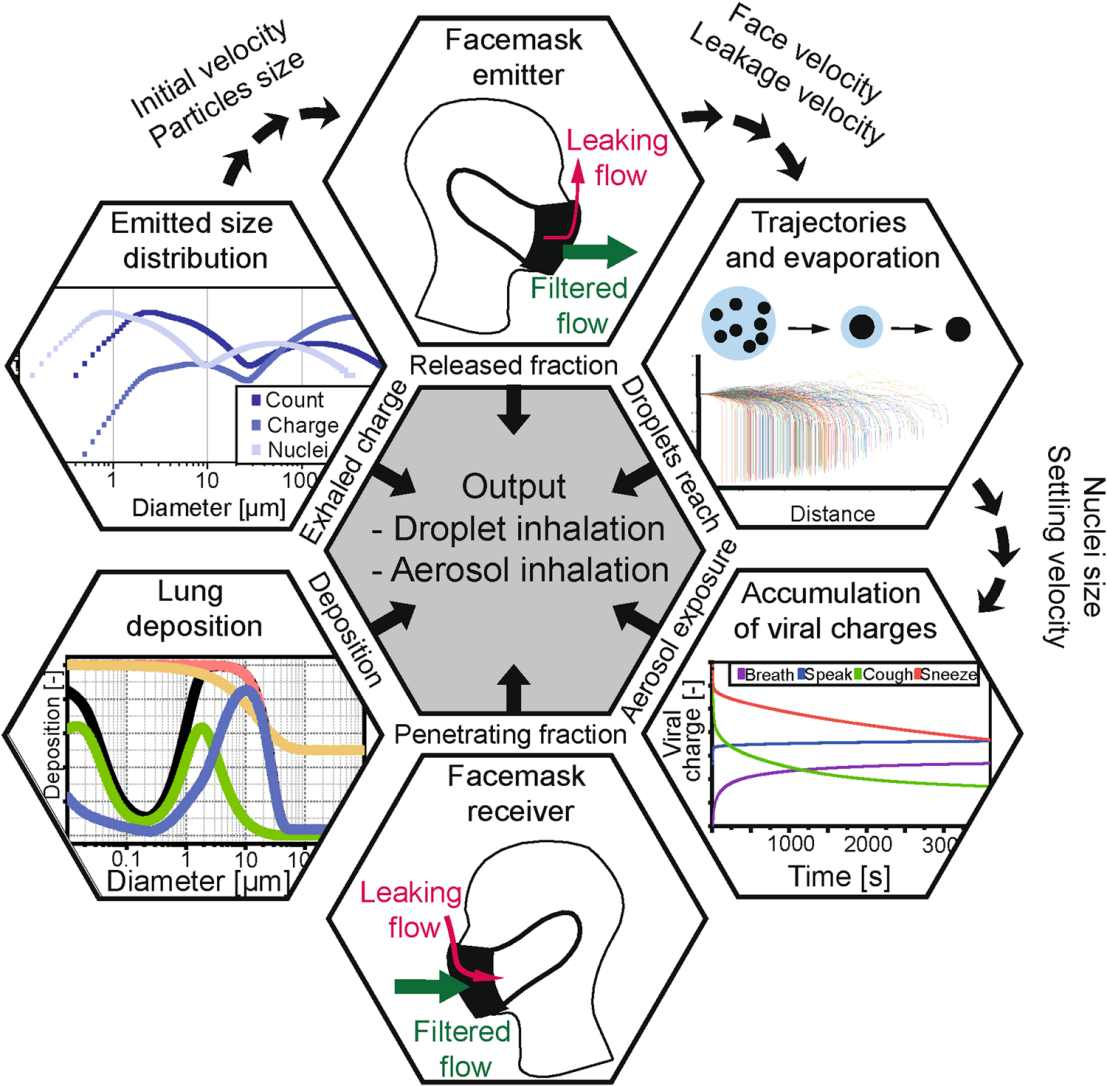
Structure of the model calculating the exposure level. The outputs from the single modules were combined to calculate the exposure levels due to droplets (near-field) and aerosol (far-field) exposures. The scenarios included different types of facemasks worn by the emitter (used as source control) and/or the receiver (used as respiratory protection) with various levels of leakage. The filtration efficiency curves of the facemasks were based on measurement data available in the literature and fitted with equations describing the filtration processes. The emission scenarios included breathing, with additional particle generation by speaking, coughing, and sneezing, and the corresponding size distributions were built from data available in the literature. The fraction deposited in the lung was calculated using the NCRP deposition model.

The most significant features of the model are summarized in the Method section, whereas a comprehensive documentation as well as the input data and hypotheses are given in Supporting Information: a theoretical background is given in section A; the implementation in Matlab R2020b is detailed in section B; a sensitivity assessment in section C; the model validation in section D with an example including detailed intermediate data, and additional data in section E. The modular model for calculation of the viral charge exposure is illustrated in Fig. 1, which was applied in different scenarios. The detailed parameters are given in Figure S1 in Supporting Information.

**Figure 2:**
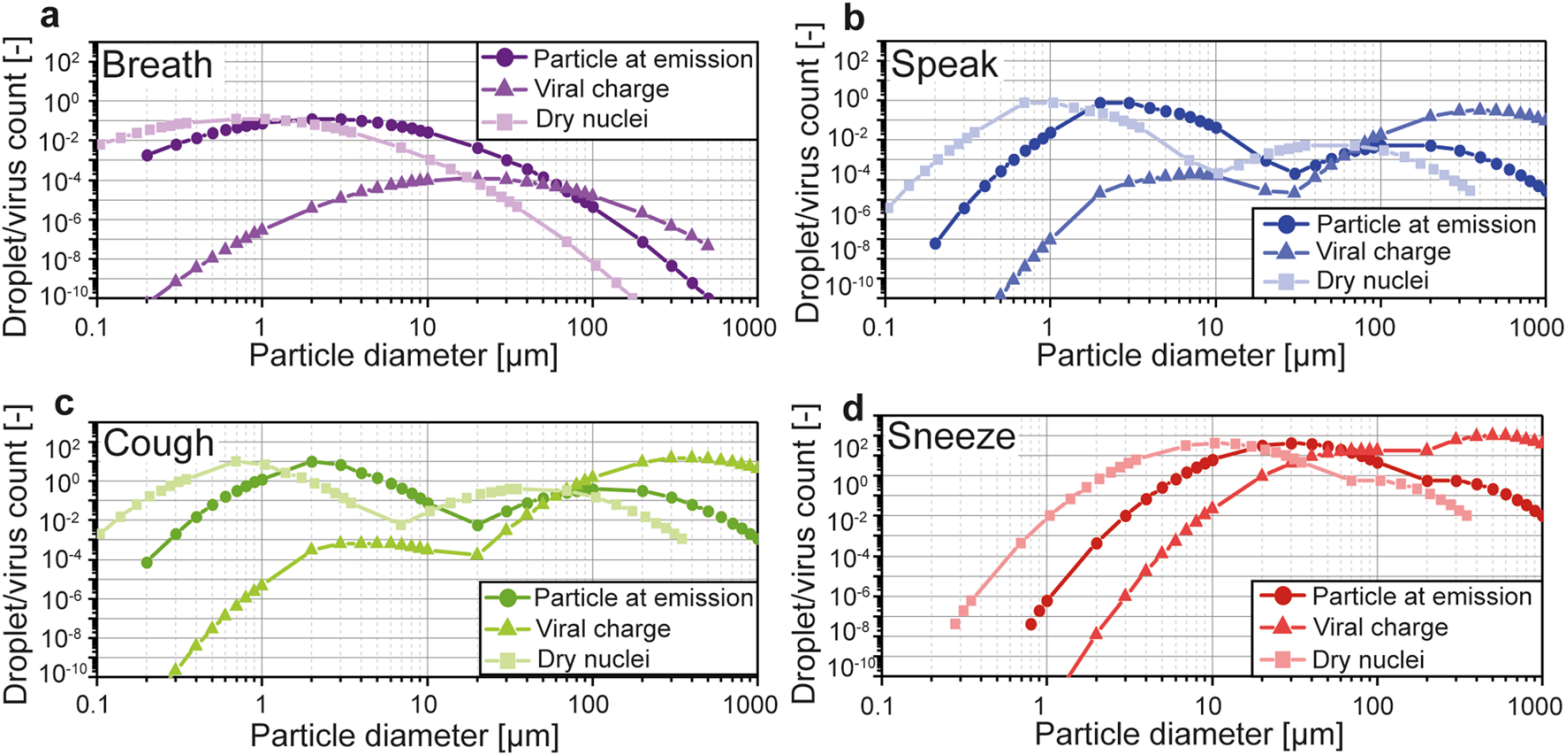
Size distributions based on the number of generated particles and the corresponding viral charge, as well as the number distributions considering the dry nuclei sizes, for breathing (**a**), speaking (**b**), coughing (**c**), and sneezing (**d**). The total particle counts in each distribution corresponds to the average values given in Table S4 and represent one minute for breathing and speaking, and one occurrence for coughing and sneezing. The viral charge was calculated considering a homogeneous concentration of viral charges in the liquid fraction of the droplets equal to 7 × 10^6^ RNA copies/mL^37^. The particle counts were normalized, taking the total number of particles emitted while breathing as reference for each size and expiratory activity. The viral charge was obtained by multiplying the normalized count and the viral charge per particle.

Two virtual individuals were considered: an infected emitter and a receiver. The terms “individuals”, “receiver”, and “emitter” used throughout this work refer to simulated entities and no tests or measurements described in the present study involved humans. The protection provided by several types of facemasks used as source control and/or respiratory protection was calculated. The size distributions for breathing, speaking, coughing, and sneezing are given in Fig. 2 and include the diameters at emission, the diameters of the dry nuclei, and the viral charge based on the volume of the droplets.

**Figure 3:**
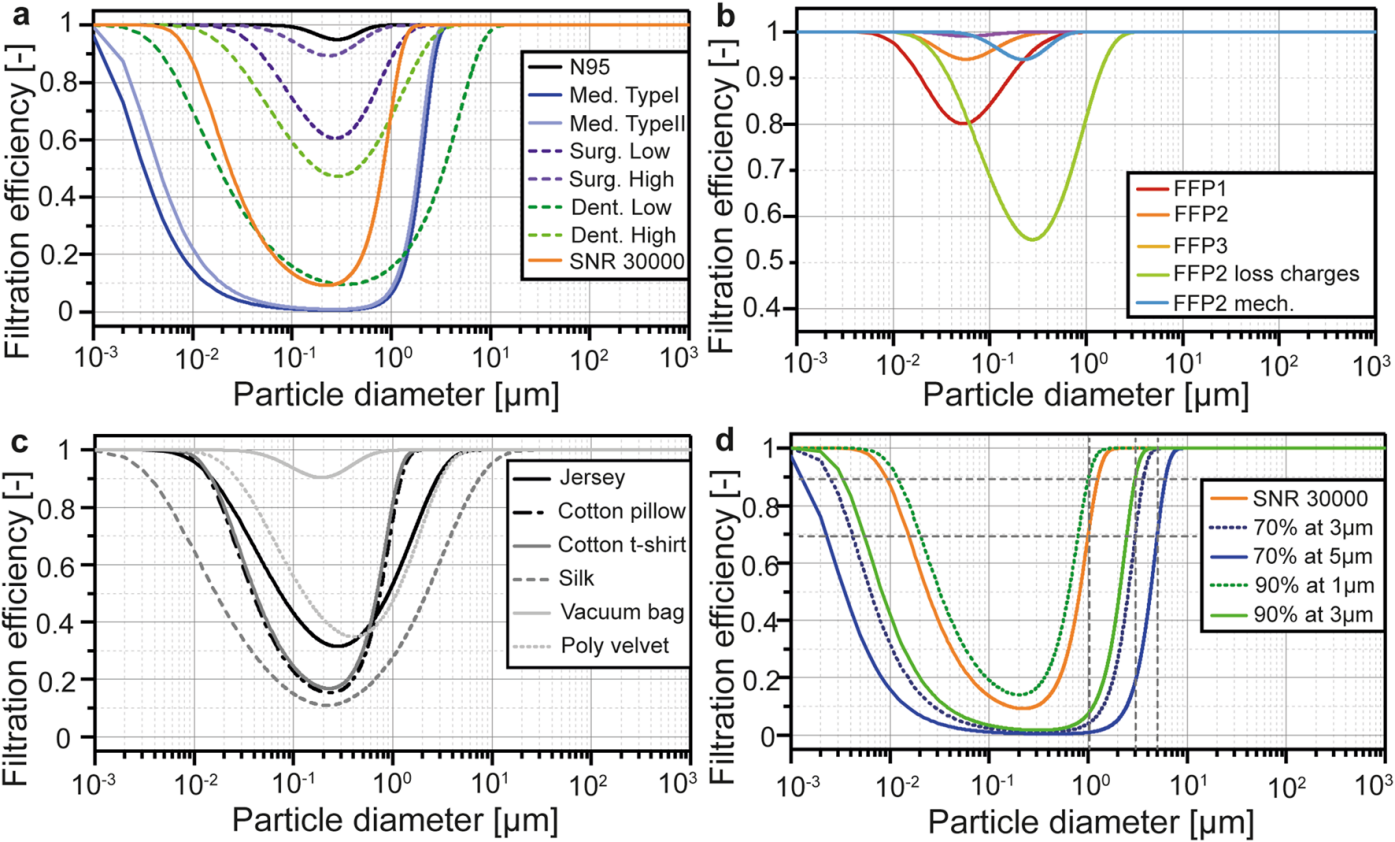
Filtration efficiency curves of the facemasks modelled and investigated in the present work, divided into three categories. Certified masks (**a**,**b**) were based on the standards EN 149 and SNR 30000 as well as on data from the literature. The different FFP-certified protections were modelled (**b**) together with additional FFP2 masks to investigate the influence of a loss of electrostatic charges and the worst-case scenario permitted by EN 149 through a FFP2 mask based on mechanical filtration. Homemade masks (**c**) were based on filtration data from the literature. Virtual masks (**d**) followed a prescribed efficiency at a defined particle size. The SNR 30000 standard is added in (**d**) for comparison purposes.

The filtration curves of the modeled facemasks at the reference face velocities are given in Fig. 3. Details on the parameters and models are given in “Method” and in Supporting information.

The exposure level, defined as the viral charges deposited in the receiver’s respiratory system, was calculated from the far-field an d the near-field exposures according to the description of the transmission routes provided by the CDC^11^. The far-field exposure corresponded to the aerosol transmission and was calculated from the average viral concentration accumulated in a space (normally a room) where the emitter and receiver were present. The near-field exposure level corresponded to the droplet transmission, and was derived from the trajectories of the emitted particles within the emitted plume and their probability to reach the receiver’s position. The calculated near-field exposure was dependent on the receiver’s position relative to the emitter, and was the highest when the receiver was on the trajectory of the emitted plume (see Figure S6 in Supporting Information). In contrast, the positions did not play a role in the far-field exposure calculation based on the uniformly mixing assumption. The far-field exposure was corrected to avoid a double-count of the viral copies already considered in the near-field exposure. The third route described by the CDC is the contact transmission, composed of the viral charges deposited on the receiver’s face without being inhaled. It was not considered in our model because we focused on the effects of masks on airborne transmission.

The carriers of viral charges emitted by the infected individual are referred to as particles in the present work. The distinction between droplets and droplet nuclei is made only to highlight the influence of evaporation.

### Spatial spread of viral charges

**Figure 4:**
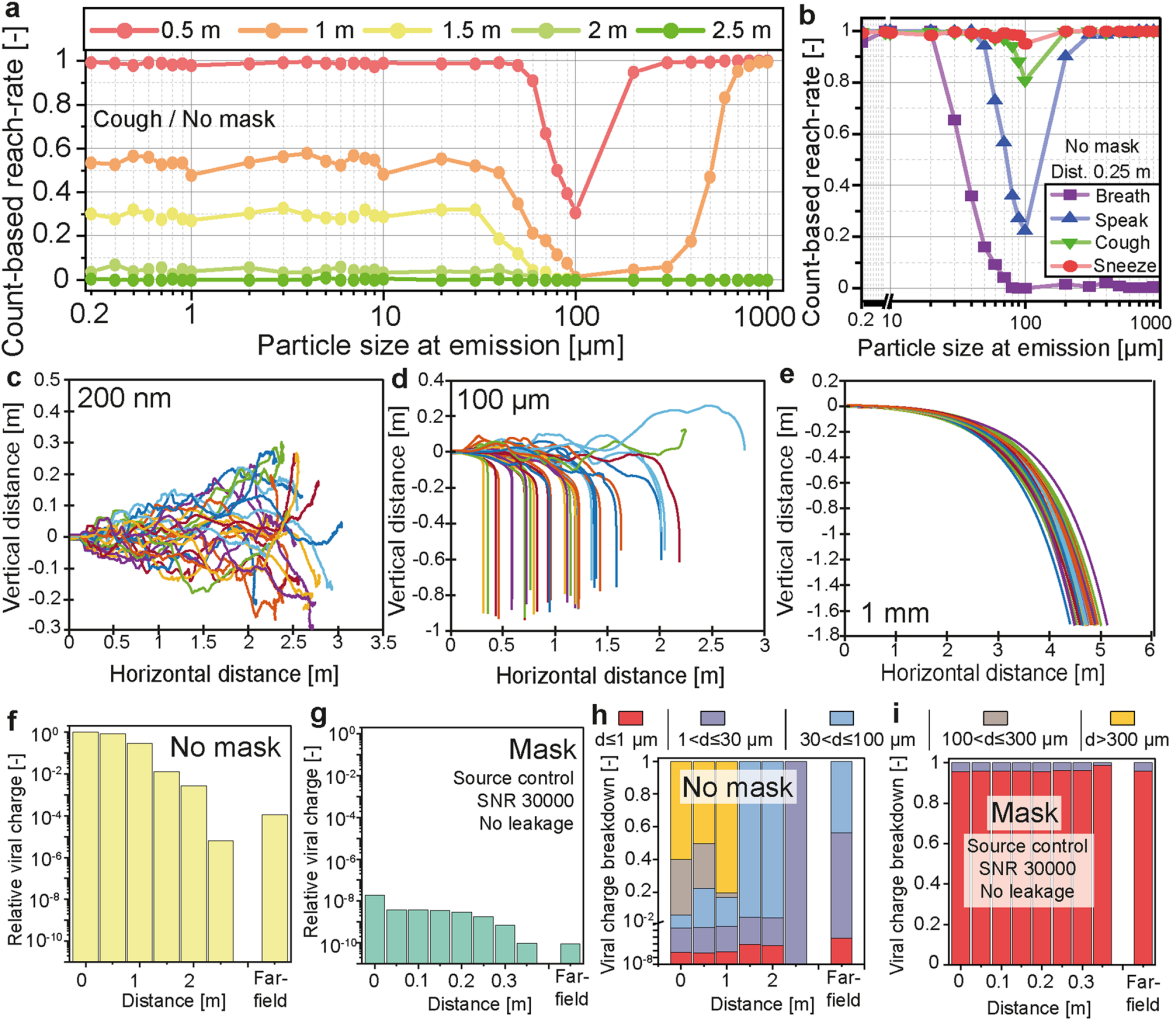
Horizontal spread of the emitted particles with and without a mask. The number-based reach-rate was calculated from the count of particles of each size reaching the face of the receiver facing the emitter and located at different distances. The data presented in (**a**) show the reach-rate considering a single cough without a facemask. The receiver’s face was represented by a 15 cm diameter disk. The reach-rates of all four expiratory activities calculated at 0.25 m without a mask are given in (**b**). Trajectories used in the calculation of the reach-rate are given in (**c**) for 200 nm particles, (**d**) for 100 μm particles, and (**e**) for 1 mm particles. The number-based reach-rate was used to calculate the near-field exposure level at different distances without a mask (**f**) and with a SNR 30000 mask used as source control (**g**). The exposure level without a mask at 0 m is taken as the reference. The particles were divided into five size classes based on the data presented in (**a**) and considering the viral charge per droplet. The distributions of viral charge into the size classes corresponding to the data from (**f**) and (**g**) are presented in (**h**) and (**i**).

The horizontal spread of virus-laden particles with and without a facemask is illustrated in Fig. 4.

The fraction of the emitted particles reaching the receiver’s facemask (referred to as the reach-rate), modelled as a 15 cm diameter disk, is given in Fig. 4a as a function of the particle size at emission, considering a single cough (not combined with breathing) without a mask. Particles were emitted with various initial positions distributed over the mouth opening. The corresponding axial and radial flow velocities, as well as the turbulences were calculated depending on the particle’s position. Particles smaller than 30 μm showed a homogeneous decrease of their reach-rate with increasing distance. These particles travelled at a velocity similar to that of the emitted airflow and remained airborne for a significant period of time (settling velocity between 2.26 × 10^–6^ m/s for a 200 nm particle and 0.027 m/s for a 30 μm particle). The emission velocity increased in the order of breathing, speaking, coughing, to sneezing, correspondingly, the reach-rate at 0.25 m significantly increased for particles larger than 30 μm along with the increasing emission velocities as shown in Fig. 4b. The trajectories of 200 nm particles are shown in Fig. 4c, for a time up to 10 s after emission. Most of the 200 nm particles stayed within the emitted plume, and its boundaries are visible from the various trajectories. Particles between 30 and 300 μm had the lowest reach-rate. As soon as they crossed the boundaries of the emitted plume (for the details of the calculation, see section A8.2 in Supporting Information), their horizontal velocity dropped and they settled on the floor rapidly. Trajectories of 100 μm particles, shown in Fig. 4d, illustrate the influence of the turbulences on the particles. The variation of their trajectories was due to their initial positions and turbulences, which influenced their position within the emitted plume. They experienced a significant drop of their settling velocity upon drying, from 0.3 m/s down to about 0.027 m/s. Larger particles, between 300 μm and 1 mm, followed ballistic trajectories, as shown in Fig. 4e, which were only influenced by their diameter, initial position and velocity. They were not significantly influenced by turbulences or drying (a 300 μm droplet lost less than 1% of its diameter before reaching the floor). The inhaled viral charge as a function of the distance between the emitter and the receiver is given in Fig. 4f (without mask) and 4 g (with a community mask as specified in SNR 30000, used for source control on the emitter with an assumed perfect fit), showing a significant decrease of the horizontal spread with a mask due to filtration and reduction of the initial velocity. The mask’s filtration characteristics led to a reduction of the inhaled viral charge by 8 orders of magnitude (initial data points in Fig. 4f,g) while the reduced initial velocity additionally lowered viral charges and led to a significant reduction of the travel distance, from 2.5 m without a mask to 0.35 m with the mask when the reach-rate, and thus the viral charge, was reduced to zero. Based on the data from Fig. 4a, the emitted particles were divided into five size classes (d ≤ 1 µm; 1 < d ≤ 30 µm; 30 < d ≤ 100 µm; 100 < d ≤ 300 µm; d > 300 µm) and the contribution of each size class to the horizontal spread of viral charges is shown in Fig. 4h without a mask and Fig. 4i with a mask. Without a mask, the fraction of the viral charge carried by larger particles (d > 100 µm) initially dominated but significantly decreased after 1 m and particles between 30 and 100 μm became prevailing. After 2 m, particles larger than 30 μm could not reach the receiver and the viral charge was exclusively carried by particles between 1 and 30 μm. The far-field exposure was mostly due to particles smaller than 30 μm accumulating around the emitter, as the larger particles settled on the floor rapidly. Filtering particles larger than 30 μm provided a significant increase of the protection level for distances within 2 m where the inhaled viral charge was dominated by the near-field exposure. The filtration performances between 1 and 30 μm were critical to further improve the protection efficiency against suspended particles (far-field exposure). Particles smaller than 1 μm were dominant in the total viral charge inhaled with a mask (Fig. 4i) and an additional reduction of the inhaled viral charge required improvement of the filtration in this size range.

### Filtration performances of facemasks

**Figure 5:**
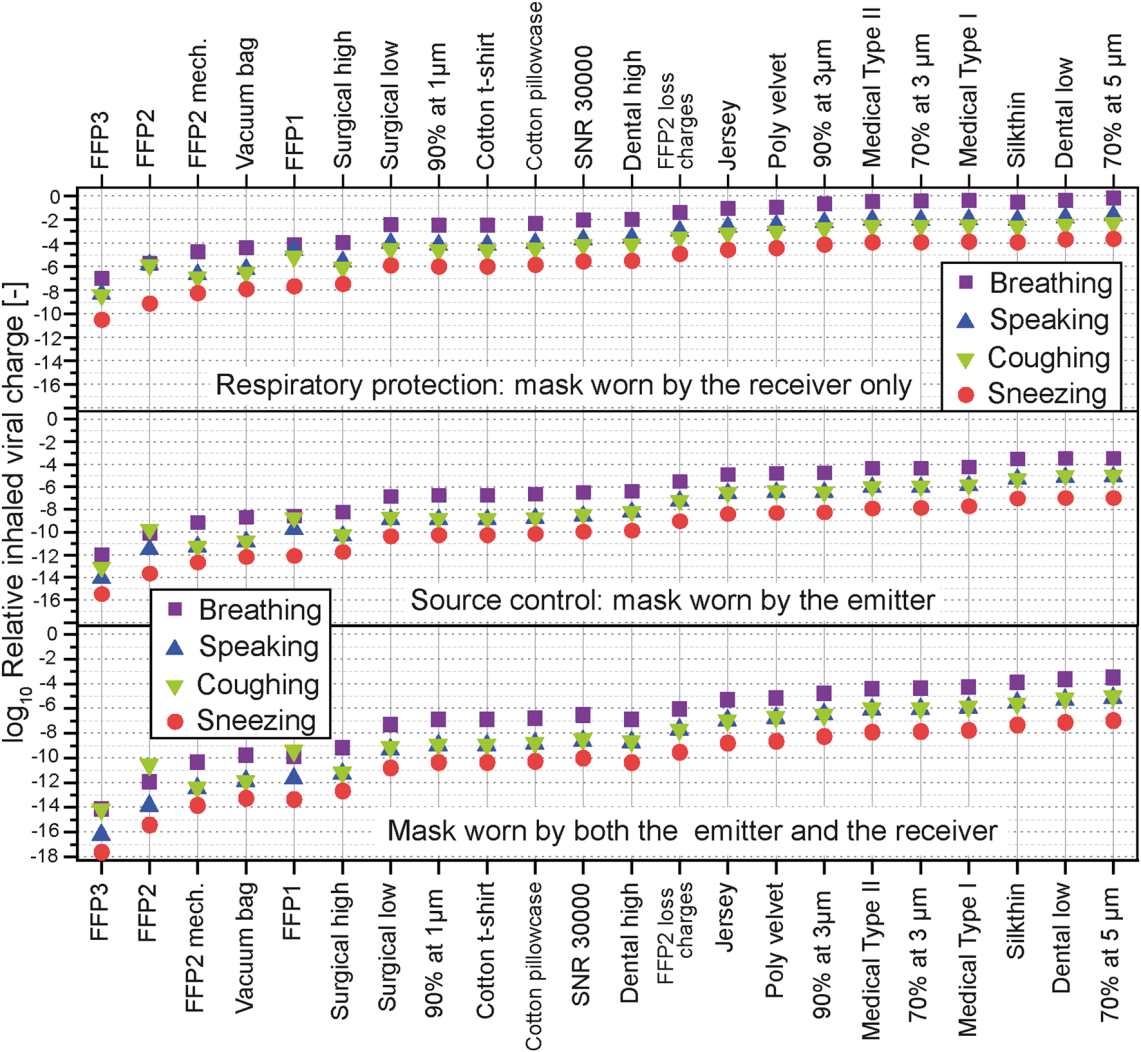
Role of different types of facemasks in reducing the inhaled viral charge. The calculations were based on the viral copies inhaled by a receiver located 0.25 m from the emitter, on the trajectory of the emitted airflow. The masks were considered to be perfectly fitted (no leakage). The data show the relative inhaled viral charge from breathing alone, and together with speaking, one cough, and one sneeze. The viral charge for each emission scenario without a mask was taken as the reference. Three different versions of FFP2 masks are presented: one based only on mechanical filtration (“FFP2 mech.”), a second one including electrostatic filtration (“FFP2”), and a third one models the loss of electrostatic charges (“FFP2 loss charges”).

The impact of the filtration properties of different types of facemasks on the inhaled viral charge was calculated with an assumption of a perfect fit on the wearer’s face (no leakage). The data are presented in Fig. 5, with the masks worn by the receiver (source control), by the emitter (respiratory protection), and by both the emitter and the receiver. The receiver was located 0.25 m away from the emitter.

The four expiratory activities all included breathing (60 min breathing, 30 min breathing and 30 min speaking, 60 min breathing and one cough, and 60 min breathing and one sneeze) as described in “Method”* section. The protection efficiency was calculated as the reduction factor of the inhaled viral charge compared to the case without a mask, and the viral charge was calculated as the sum of the near-field and far-field exposure levels. The masks were ranked based on their protection efficiencies. The highest protection was provided when the masks were used both as source control and respiratory protection (average reduction of the inhaled viral charge by 10 orders of magnitude compared to the no-mask scenario), and source control was significantly more efficient than respiratory protection (respectively 8 and 4 orders of magnitude lower inhaled viral charge). Noticeable differences appeared between various modeled masks, and reflected the different filtration requirements (efficiency at a defined particle size) by the respective standards, with an average difference of 7 orders of magnitude between the FFP3 and the mask labeled 70% at 5 µm. Used as source control, the facemasks reduced the velocity of the exhaled airflow, and therefore the travel distance of the emitted particles. The particles were filtered before their evaporation, hence with a higher efficiency as their diameters were larger. The masks used for respiratory protection provided a lower protection level as most of the particles reached evaporation equilibrium before being filtered and the size distribution was shifted toward smaller diameters, where masks generally had a lower filtration efficiency, except for particles below the most penetrating particle size (MPPS). In addition, the velocity of the incoming particles decreased due to the drag force of air and was generally lower than the velocity at emission (except for breathing, as the breathing flow was considered to have the same velocity for both the emitter and the receiver), reducing the filtration efficiency by inertial impaction.

The relative protection efficiency increased for all masks in response to a higher initial velocity and larger particles sizes.

The FFP2 mask including electrostatic filtration benefited from a lower MPPS and provided a higher protection than its counterpart based on mechanical filtration only (FFP2 mech.) with the same minimal filtration efficiency but at a higher MPPS (the inhaled viral charge was on average reduced to 1/12 in the breathing case). The protection efficiency significantly dropped upon loss of the electrostatic charges (the inhaled viral charge was on average 3.2 × 10^5^ times higher in the breathing case) as the minimum filtration efficiency decreased while the MPPS increased as shown in Fig. 3b. The FFP3 mask provided the highest protection efficiency. The SNR 30000 mask, specified as 70% at 1 µm, provided a higher protection than the Type I and Type II medical masks, specified as 95% at 3 µm, indicating the importance to define the minimum filtration efficiency at a lower particle size to provide a higher protection. This result can also be clearly seen from Fig. 3a where the filtration efficiency curve of SNR 30000 is above those of the Type I and Type II medical masks. The SNR 30000 provided on average a 55% lower protection than the mask requiring 90% at 1 µm; in contrast, it provided a significantly higher protection than the other virtual masks. These results were in agreement with the filtration efficiency curves for virtual masks shown in Fig. 3d. Additional data and discussion are available in section E1 in Supporting Information.

### Impact of the leakage

**Figure 6:**
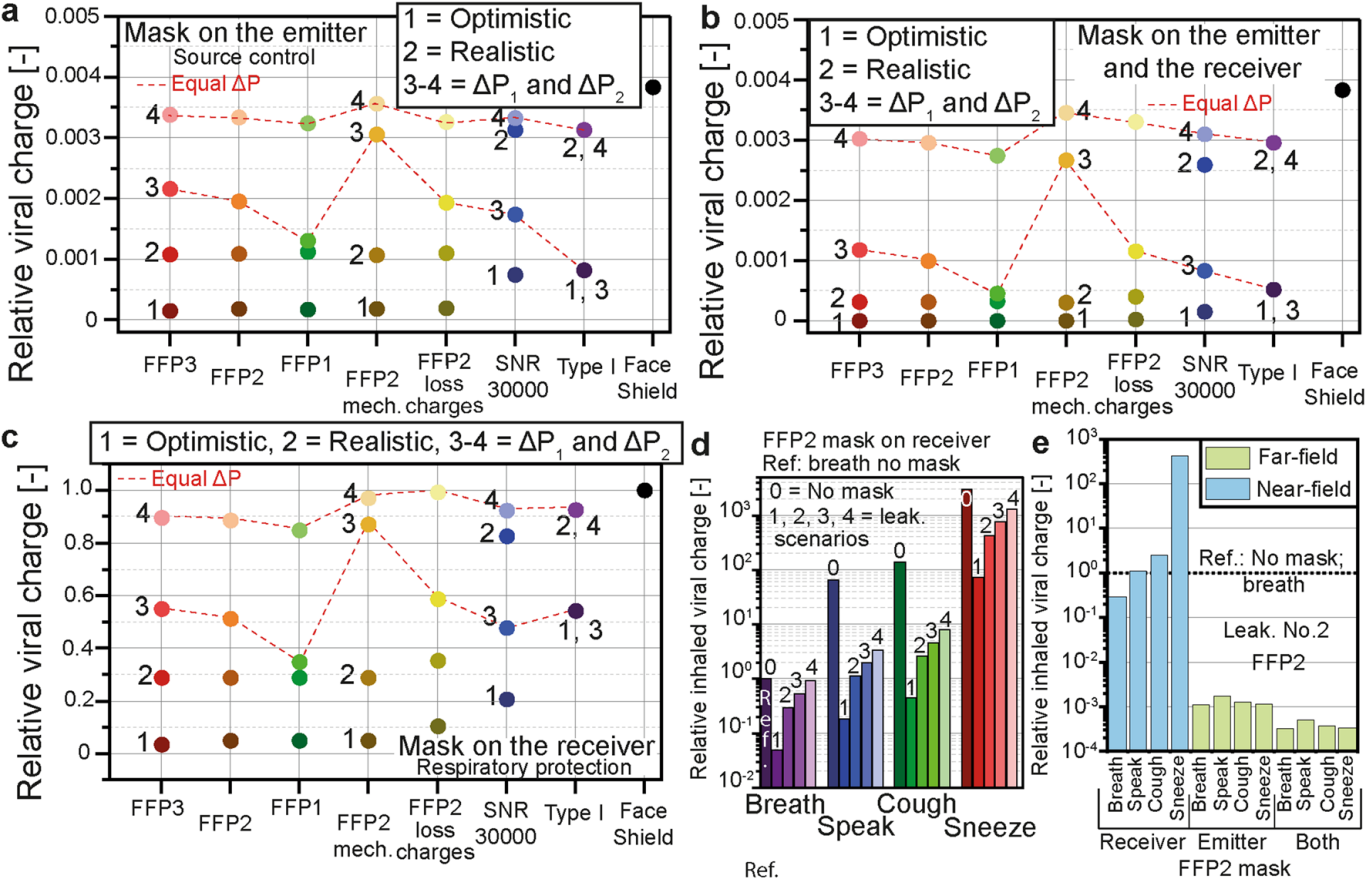
Influence of the leakage. The viral charge inhaled by the receiver located at 0.25 m from the receiver on the trajectory of the emitted airflow was calculated with different types of facemasks in several scenarios of leaking flows. The inhaled viral charge while breathing without a mask was taken as the reference. Scenario No.1 reflected an optimistic fit satisfying EN 149; No.2 represented a realistic fit; No.3 and 4 represented degraded fittings based on the hypothesis of equal breathing resistance for all the masks: the pressure drop was set to the value generated by a Type I mask in the optimistic fit scenario in No.3 and in the realistic fit scenario in No.4. The resulting exposure level, calculated as the sum of the near-field and far-field exposures, is given in (**a**) when only the emitter wore the mask ( source control), in (**b**) both the emitter and the receiver wore the masks, and in (**c**) only the receiver wore the mask (respiratory protection). All the levels of leakage applied to breathing, speaking, coughing, and sneezing are compared for a FFP2 mask on the receiver in (**d**). The no-mask scenario for the different expiratory activities is included for comparison and marked No.0. The breakdown of the inhaled viral charge into near-field and far-field exposures in the cases of speaking, coughing, and sneezing is given in (**e**) for leaking scenario No.2 for a FFP2 mask.

The impact of the different leaking scenarios on seven types of masks and a face-shield is presented in Fig. 6.

Scenario No.1 represented an optimistic fit based on the maximum allowed leakage according to the EN 149 standard. The leakage value prescribed for FFP3 masks (20%) was extended to the other types of masks. Scenario No.2 represented a realistic fit and was based on data gathered from the literature to represent the fit factors likely to be found among non-trained users. Scenarios No.3 (also named ΔP_1_) and No.4 (ΔP_2_) represented degraded fitting conditions based on the assumption of a similar pressure drop generated by the masks: in scenario No.3, the masks were fitted to generate the same pressure drop as a Type I mask in scenario No.1. In scenario No.4, they were fitted to generated the same pressure drop as a Type I mask in scenario No.2. Further details are given in “Method” section. The data were normalized taking the exposure level without protection as the reference. The total viral charge inhaled by the receiver was calculated when only the emitter wore the mask (Fig. 6a), both individuals wore the same type of mask (Fig. 6b) and only the receiver wore the mask (Fig. 6c); particles were generated by breathing. In scenarios No.1 and No.2, the FFP masks provided a significantly better protection than Type I and SNR 30000 masks. The relative values of the exposure levels were close to the relative leaking fraction when the masks were worn by the receiver only (Fig. 6c): the relative exposure was 0.289 for the certified FFP masks in the realistic scenario (No.2), and the corresponding leaking fraction was 29%. The FFP2 after discharge led to a higher exposure (0.35) for the same leaking fraction due to the strongly reduced filtration efficiency. The SNR 30000 and Type I medical masks showed relative exposure of respectively 0.83 and 0.93 for a leaking fraction of 82%. The exposure levels were significantly lower when the emitter wore a mask (Fig. 6a,b): the exhaled leaking flow was directed upward by the mask and the released particles rapidly settled on the floor or accumulated around the emitter (far-field exposure) instead of directly reaching the receiver through the exhaled plume (near-field). The protection efficiencies of the FFP masks significantly decreased when the leaking fraction was adjusted to match a lower breathing resistance. In scenarios No.3/ΔP_1_ and No.4/ΔP_2_, the FFP and SNR 30000 masks used for source control (Fig. 6a) provided a lower protection than the Type I mask due to their higher flow resistance leading to a stronger leakage. These results reflected the differences in breathing resistance, as FFP masks were modeled with pressure drops of 45 Pa (FFP1), 60 Pa (FFP2), and 65 Pa (FFP3) at 5.3 cm/s, the SNR 30000 with 294 Pa at 27.2 cm/ and the Type I mask with 196 Pa at 27.2 cm/s according to data from the literature and the respective standards (further details are given in section A3 in Supporting Information). The FFP masks used for respiratory protection (Fig. 6c) provided similar protection as the Type I mask, except the mechanical FFP2 mask, exposing the receiver to a significantly higher viral charge in scenario No.4 as it was modeled with a higher pressure drop than the standard FFP2 including electrostatic filtration (240 Pa at 5.3 cm/s according to EN 149). Notable differences appeared among the FFP masks and reflected their different breathing resistances, as well as differences among the three modeled FFP2 masks. The corresponding discussion is available in section E2 in Supporting Information. The face-shield was the least efficient among the considered protection devices. It was effective when used for source control, as it blocked the emitted plume and diverted the particles to the upward leaking flow (significant reduction of the exposure in Fig. 6a,b) but its efficiency dropped when used as respiratory protection (Fig. 6c, 99% of the viral charge without a mask was still inhaled with a face-shield). It did however reduce the near-field exposure generated by speaking, coughing, and sneezing as it blocked the incoming droplets traveling within the emitted plume. Further data are provided in section E3 in Supporting Information.

The exposure levels in the different leaking scenarios for a FFP2 mask worn by the receiver are compared for all the four expiratory activities in Fig. 6d. The viral charge inhaled without a mask for each expiratory activity is marked No.0, and the value for breathing was taken as reference. The presence of the leakages led to a significant increase of the inhaled viral charge compared to the no-leakage scenario. Considering a FFP2 mask worn by the receiver, the viral charge increased by a factor 5 × 10^4^: the mask removed 99.998% of the viral charges in the no-leakage scenario compared to the no-mask case, and 95.158% in scenario No.1 (down to 10.056% in scenario No.4). In the no-leakage hypothesis, 100% of the inhaled viral charge came from the mask flow, while in scenario No.1, 99.965% of the inhaled viral charge came from the leaking flow (up to 99.998% in scenario No.4). The preponderance of the leakage contribution to the total exposure level is aided by the higher average size of the particles released with the leaking flow compared to the particles penetrating the masks, thus carrying a higher viral load. Detailed data are given in section E4 in Supporting Information. The level of leakage was the main factor leading to the ingress and release of viral copies and was dominant over the filtration performances of the masks, showing that the no-leakage scenario did not realistically describe the level of protection provided by the masks. The exposure levels resulting from a leakage in the realistic fit scenario (No.2) on a FFP2 mask were compared among the different expiratory activities and the results are given in Fig. 6e. The exposure from breathing without a mask (not shown in Fig. 6e) was taken as the reference. The near-field exposure route was the dominant contributor to the number of inhaled viral charges when the mask was used as respiratory protection, while the far-field route was dominant when the mask was used for source control. Used for source control, the mask reduced both the spread of viral charges and filtered the emitted droplets, significantly reducing the near-field exposure. A fraction of the smaller droplets escaping through the leakage was able to stay suspended around the emitter and contributed, together with particles penetrating the mask, to the far-field exposure. Used as respiratory protection the masks filtered the incoming airflow and the near-field exposure was dominant. Further data are available in section E5 in Supporting Information.

### Limitations of the proposed model

Our model is an applied approach integrating a large number of existing models in order to provide numerical results for a wide variety of scenarios and parameters regarding the effects of facemasks on airborne virus transmission, and has several limitations. The calculation of the leaking flow and the fraction of particles traveling though the leakages did not take into account the turbulences within the leaking airflow, which are likely to decrease the fraction of particles released into the environment. The leakage model assumed a single gap and directed the flow upward, whereas gaps between the mask and the wearer’s head are likely to form at other locations and have a significant impact on the leaking flow. Furthermore, the surface roughness and the shape of the path leading to the gap have not been considered. The deformation of the mask, especially during events generating a high pressure in the volume enclosed by the mask and the wearer’s face such as coughing and sneezing, was not considered and might dynamically modify the fit factor. We focused on the interactions between the mask and the emitted particles and did not describe the complex interactions between the airflow and the mask. Finally, the impact of the inhalation following an exhalation on the emitted particles was not considered. These limitations leave room for future improvement of the model.

## Conclusion

The computational model presented in this study provides a comparison of the different factors influencing the level of protection provided by facemasks against virus-laden particles: horizontal spread of contaminated particles, filtration efficiency, and leakage. The results from different scenarios show that all the modelled facemasks provide a significantly higher protection when used as a source control rather than as a respiratory protection. FFP masks have a higher filtration efficiency than surgical or community masks and provide a better protection if they are fitted accordingly to minimize the leakages. By design, FFP masks provide a better fit around the faces of the users compared to surgical masks and minimize the leakages, as shown by the fit factors taken from the literature and used in the leaking scenario No.2. However, their higher pressure drop leads to a higher level of leakage in case of improper fitting and their protection efficiency drops below properly worn surgical and community masks. Face-shields do not constitute an efficient alternative to facemasks especially in case of inhalation protection.

## Method

### Size distributions of the exhaled particles

The size and number of particles generated from breathing, speaking, coughing, and sneezing are affected by numerous parameters. Loud singing^38^, shouting^39^ as well as the language^40^ influence the size distribution. The number of particles generated during a cough is higher for patients having an infection of the upper respiratory tract^41^ or infected by influenza^42^. High inter- and intrapersonal variabilities were reported in the emitted number and sizes of generated particles^42–45^. The particles generated by the four expiratory activities considered in this work were described by their modality and total counts. Additional activities generating particles such as mechanical ventilation, bronchoscopy or surgery^46^ were not considered.

The number-based size distributions of the emitted particles while breathing was considered as unimodal; speaking and coughing emissions were modelled by trimodal distributions according to the Bronchiolar/Laryngeal/Oral (B.L.O.) model, where each mode is linked to the site of origin of the particles^47^. The size distribution generated during a sneeze was considered to be bimodal^48^. The detailed calculation of the distributions is given in section A1 in Supporting Information. A dynamic evolution of the diameter of the evaporating droplets was applied for the computation of the trajectories. Droplets were considered to be composed of a liquid phase with dissolved and suspended species including salts (NaCl, KCl), pulmonary surfactant, cells (epithelial cells and cells from the immune system), proteins, bacteria, and viruses^49,50^. Upon drying, the dissolved and suspended species form a solid nucleus. The detailed calculation of the nuclei size and the evaporation rate is given in section A8.1 in Supporting Information. Stable ambient conditions of 50% relative humidity, 20 °C ambient temperature, and 37 °C initial droplet temperature, were used in this work.

The number of emitted particles were compiled from data published in numerous studies^44,47,51–58^ and the total counts for each activity were modelled as a lognormal distribution. The calculations presented in this work were based on the median value and were normalized, taking the count emitted during one minute breathing as 1. The resulting relative emission number was 3 for one-minute speaking, 30 for one cough and 225 for one sneeze. The emitted viral charge was proportional to the total volume of the emitted droplets. The normalized emission by total volume was 1 for breathing (the reference), 1.96 × 10^3^ for speaking, 9.48 × 10^4^ for coughing, and 7.22 × 10^5^ for sneezing. The detailed emission numbers are available in Table S3.

The size distributions based on the droplet count and the viral charge are given in Fig. 2. The viral charge for each droplet ${C}_{V}$ was calculated according to Eq. (1), based on the diameter at emission ${d}_{init}$. Viral copies were considered to be homogenously distributed in the liquid phase of the droplets with a concentration ${L}_{v}$ equal to the concentration in the pulmonary fluid. The average concentration measured in sputum is 7 × 10^6^ RNA copies/mL for SARS-CoV-2^37^. This value is likely to show a significant inter- and intra-personal variability, depending on factors such as the number of days since infection, the site of origin of the droplets, or the vaccination status^37,59–61^. The influence of the viral concentration on the calculation of the exposure level was performed and is available in section C in Supporting Information. The value of 7 × 10^6^ RNA copies/mL was applied to all four expiratory activities.

1 $${{C}_{V}=L}_{v}\cdot \frac{4}{3}\pi \cdot {\left(\frac{{d}_{init}}{2}\right)}^{3}$$

The model covers the initial droplets sizes from 200 nm to 1 mm. The diameter of the SARS-CoV-2 lies between 60 and 140 nm^62^, therefore particles with a diameter below 200 nm, corresponding to a dry diameter of 70 nm, are highly unlikely to carry any complete copy of the virus. The maximum diameter was set to 1 mm as most of the models and measurements available in the literature considered particles up to this size^47,48^. Unless stated otherwise, the emission distribution for speaking was combined with nose breathing (both activities were considered for the same duration, alternating speaking and breathing), coughing and sneezing were considered as a single activity and were combined with nose breathing.

### Filtration efficiency of facemasks

The filtration efficiencies of different types of masks and filtering materials were gathered from the literature and several standards (EN 149, SNR 30000, and EN 14683) for different particle sizes and face velocities. The filtration efficiency curves for particle sizes up to 1 mm were reconstructed using the equations describing the single-fiber efficiency for diffusion, interception, interception of diffusing particles, inertial impaction, and electrostatic filtration^63–66^. The filtration mechanisms outside of electrostatic effects are together referred to as mechanical filtration mechanisms.

The modelled masks were divided into three categories: certified masks, homemade masks, and virtual masks. The properties of certified masks (filtration efficiency, breathing resistance, and leakage) were based on the standards previously mentioned. The following masks were considered: FFP1 (Filtering Facepiece respirator), FFP2, and FFP3 masks, Medical Type I and II masks, as well as community masks based on SNR 30000. FFP1, FFP2, and FFP3 face protections were modelled based on the minimal filtration efficiency required by the EN 149 standard and their pressure drops taken from measurements data available in the literature^67^. The same pressure drop values were taken for both inhalation and exhalation. Filters based on electrostatic filtration have a smaller most penetrating particle size (MPPS) compared to equivalent filters based on mechanical filtration^68,69^. Both mechanical and electrostatic filtration mechanisms may contribute to the filtration capabilities of respirators. However, the electrical charge on the respirator decays with usage^67^. The influence of a total loss of electrostatic charges was investigated with a mask referred to as “FFP2 loss charges”. To take a conservative approach and assume that the FFP filtration curve could still fulfill the requirements of the EN 149 standard without the electrostatic contribution, an additional FFP2 mask (named “FFP2 mech.”) was modelled considering only mechanical filtration, to reach a MPPS close to 350 nm. The breathing resistance of electrostatic filters is generally lower compared to their mechanical counterpart^70,71^. The pressure drop of the mechanical FFP2 filter was therefore set to the maximum value permitted by the EN 149 standard (240 Pa at 5.3 cm/s at inhalation, and 300 Pa at 8.5 cm/s at exhalation). The breathing resistances of the other types of masks was taken from the respective standards (e.g., 294 Pa at 0.27 m/s for SNR 30000, see Table S5 in Supporting Information). In order to reflect the variability in filtration performances of medical masks, additional measurement data from the literature^72,73^ on two masks used in medical facilities (labelled as surgical high and surgical low) and two masks used in dental facilities (labelled as dental high and dental low) were adopted. Homemade masks are fabricated using various materials and do not follow any specific requirement in terms of filtration efficiency or breathing resistance. The considered materials include jersey, cotton, silk, a vacuum bag, and velvet^73–75^. The third category explores virtual masks which satisfy prescribed filtration efficiencies at a given particle size under requirements for the breathing resistance according to the SNR 30000:2021 standard. The modelled masks were defined by a minimum filtration efficiency at a prescribed particle size (e.g., 90% filtration for 1 μm particles) and were compared to the SNR 30000 standard (70% filtration efficiency for 1 μm particles). By varying both the prescribed filtration efficiency and the corresponding particle diameter, the resulting protection level was calculated to search for the better protection against virus exposure. The filtration efficiencies of the masks are given in Fig. 3 and the detailed data and method to calculate the filtration curves are given in sections A2 and A3 in Supporting Information. The modelled masks did not include an exhalation valve, which would significantly reduce the protection efficiency of the masks used as source control^76^.

### Estimation of the leakages

The leakage was quantified as the fraction of the exhaled or inhaled airflow that did not go through the facemask. Based on simulations and optical measurements^77–80^, most of the leakage occurs around the nose and the outward leaking flow is directed upward. The same leakage location was considered for the inward leakage and the flow was directed toward the nose/mouth of the receiver. The leaking flow ${Q}_{leak}$ was calculated according to Eq. (2) as a fraction of the total volumetric flow ${Q}_{total} .$ The non-leaking flow ${Q}_{mask}$ went through the mask where the particles were filtered. The total flow was calculated from the velocity ${U}_{total}$ (equal to the flow velocity of inhalation or exhalation) and the diameter ${D}_{opening}$, which is linked to the area of the opening (nose opening for nose breathing, and the opened fraction of the mouth for speaking, coughing, and sneezing). The diameter of the opening was set to 2 cm for a cough^31,81^; the same value was used here for the other exhaling activities.

2 $${Q}_{total}={Q}_{leak}+{Q}_{mask}={U}_{total}\pi {\left(\frac{{D}_{opening}}{2}\right)}^{2}$$

The leakage was redirected through a gap located around the nose and considered as a simplified model to calculate the flow escaping along the contact area between the wearer’s face and the mask. The ratio between the leaking and the mask flow was determined by balancing the pressure drop generated by the facemask and the gap^78^. The detailed method is given in section A4 in Supporting Information. A fraction of the particles was not able to follow the change of direction of the leaking flow and were kept in the mask flow. This behavior was approximated by considering an impactor based on the emission velocity (for the emitter’s mask) or the inhaling velocity (for the receiver’s mask) and the opening diameter. The facemask was considered as an impactor plate for the leaking flow. The particles smaller than the calculated cut-off size followed the leaking flow. The larger particles were removed from the leaking flow, kept in the mask flow, and filtered. Detailed information is given in section A5 in Supporting Information.

Wearing a facemask reduces the velocity of the exhaled airflow as it is spread over the mask’s surface. Simulation data and optical measurements^18,19,78^, suggest that the exhaled flow mostly spreads over a part of the facemask’s surface located between the nose and the lower part of the chin. The diameter of this surface was estimated to be 10 cm; however, this value showed a high interpersonal variability^82^. The velocity of exhalation was calculated as 1.6 m/s for breathing, based on a flow of 30 L/min emitted from the mouth or nose, considering an opening surface of 3.14 cm^2^. The initial flow velocity was set to 3.9 m/s for speaking^53^, 11.7 m/s for coughing^53,83^, and 20 m/s for sneezing^84^. The presence of the mask reduced the velocities to 0.064 m/s for breathing, 0.156 m/s for speaking, 0.468 m/s for coughing, and 0.8 m/s for sneezing. These velocities were adjusted depending on the leaking flow. The airflow generated by breathing was oriented downward with an angle of 45° to simulate nose breathing. The airflow generated by speaking, coughing, and sneezing was considered to be horizontal and oriented forward. The receiver inhaled through the nose at a constant rate of 30 L/min. The influence of the angle of the flow on the particles’ inhalability in nose breathing was considered in the lung deposition model (see section A11 in Supporting Information).

Different leaking scenarios were constructed to represent the variability in fitting quality likely to be found in the practical usage of masks by the general population. Scenario No.1, also named “Optimistic fit” was based on the maximum total inward leakage allowed by the EN 149 standard for the FFP-certified masks. As they only specify the inward leakage, the outward leakage was assumed to take the same values. EN 14683 and SNR 30000 do not specify leakage, thus the value for the FFP1 given in EN 149 (20%) was extended to all the other types of masks. Scenario No.2 was named “Realistic fit” and based on fit factors reported in the literature. The fit factor is measured during a fit test and is defined as the ratio between the outside concentration of particles and the concentration inside the mask^85^. The lowest fit factor found in the literature was 3 for a FFP2 mask and 1.2 for a surgical mask^86^. The measurement of the fit factor is not specific to a particle size. The leaking flow was estimated from the fit factor considering the minimum filtration efficiency required by the standards. The resulting leaking fractions were 0.29 (FFP2) and 0.82 (surgical). The method used to estimate the leaking fraction from the fit factor is detailed in section A6 in Supporting Information. To focus on the influence of the different filtration properties on the total inhaled viral charge, the same leaking fraction was used for FFP1, FFP2, and FFP3 masks (0.29) on one hand, and for SNR 30000 and Type I masks on the other hand (0.82).

The aim of the last two scenarios was to estimate the impact of the breathing resistance on the protection efficiency in case of degraded fit conditions: facemasks increase the effort needed to breath due to their pressure drop (ΔP), which is usually higher for certified FFPs compared to surgical masks. Individuals wearing FFP masks for a long period of time might move or adjust the facemasks to decrease the pressure drop, increasing the leakage and further decreasing the overall protection efficiency. The following configurations were considered:

- Scenario No.3 (ΔP_1_): the leaked flow and mask flow were distributed such that the FFP and SNR 30000 masks generated the same pressure drop as a Type I mask in scenario No.1;
- Scenario No.4 (ΔP_2_): the FFP and SNR 30000 masks generated the same pressure drop as a Type I mask in scenario No.2.

The detailed data for each scenario are given in section A7 in Supporting Information.

In addition to the masks, a face-shield was modelled as a fully-leaking and non-penetrable facemask. Worn by the emitter, the face-shield completely blocked the horizontal spread of the emitted airflow and particles, and diverted the emission upward with the leaking flow, where the particles were released into the environment if their diameters at emission were smaller than the cut-off size of the modelled impactor. Worn by the receiver, the face-shield offered a protection against near-field exposure.

### Transport, evaporation, and accumulation of particles

The trajectories of the particles within the emitted plume were calculated using a turbulent round jet model^31,81,87^ to describe the exhaled airflow in the four expiratory activities. The particles were released with an initial velocity given in the previous section for the different expiratory activities. Only exhalation was considered for breathing, and it was modeled as a cyclic event taking into account the breathing frequency and the duration of an exhalation, with particles released at every exhalation. A similar pattern was used for speaking, while coughing and sneezing were modeled as a single event with particles only emitted once. The velocity of the exhaled airflow, including components of local turbulences, was a function of the axial and radial distances from the release point. The air surrounding the emitted plume was considered quiescent. The resulting particle’s velocity was calculated with consideration of its drag force, Brownian diffusion, and settling velocity. The particle diameter (influenced by evaporation), position, and velocity were updated at each time step (5 ms) until it reached the receiver or settled on the floor. A time limit was set to 10 s to keep the computation time reasonable for efficiently describing the highly dynamic events (fast-settling particles, evaporation of small droplets, movement of particles with a low relaxation time). Simulations of extended time showed no further evolution of the output after 10 s (more data in section C of Supporting Information). The output was the fraction of particles of each size reaching the receiver’s face, named reach-rate. It was multiplied by the particle count generated during one minute (breathing, speaking) or a single event (cough, sneeze), scaled to the desired interaction time (one hour or one event), and multiplied by the viral charge per droplet, the fraction deposited in the lungs, and the transfer functions of the masks to calculate the total near-field exposure level. The complete trajectory model is available in section A8 in Supporting Information.

The far-field exposure level was based on the accumulation of airborne viral charges, occurring over a time scale of several minutes to hours (one hour was considered in the present work) and mostly composed of slow-settling and slow-moving particles remaining suspended for minutes or hours, spread by Brownian diffusion and movement of the ambient air e.g. room ventilation. The ambient air movement was neglected in the near-field model, however, plays a role when the time scale is extended as in the far-field model. The evolution of the concentration of airborne viral charges $\frac{dc}{dt}$ is given in Eq. (3)^22^, calculated as a function of the ventilation rate $Q$, the settling velocities on upward-facing (${v}_{du}$) and downward-facing (${v}_{dd}$) horizontal and vertical (${v}_{dv}$) surfaces (respectively ${S}_{u}$, ${S}_{d}$, and ${S}_{v}$), the decay rate of the virus $\lambda$ (derived from its half-life, taken as 1.1 h^88^), the volume of the room ${V}_{m}$, the viral shedding $\sigma$, and the concentration of airborne viral charges $c$. The viral shedding was calculated from the particle count generated by the corresponding expiratory activity and converted into the number of viral charges emitted every second (only emitted once if sneezing and coughing were considered). The concentration of airborne viral charges was considered as homogenous in a space (normally a room) around the emitter. The results presented in this work were based on a volume of 75 m^3^ (5 × 5 m^2^ surface and 3 m height) considering one air change per hour.

3 $${V}_{m}\frac{dc}{dt}=-\left(Q+{v}_{dv}\cdot {S}_{v}+{v}_{du}\cdot {S}_{u}+{v}_{dd}\cdot {S}_{d}+\lambda \cdot {V}_{m}\right)\cdot c+\sigma$$

The complete model is available in section A9 in Supporting Information. The inhaled volume was calculated based on the breathing frequency and lungs’ tidal volume. The total interaction time was set to one hour, with a fixed breathing frequency of 0.25 Hz regularly paced and inhalation representing 50% of the total breathing time. Details on the calculation of the viral shedding and the inhalation volume are given in section A10 in Supporting Information.

The diameters of the particles at emission were used for the calculation of the filtration efficiency of the emitter’s mask. The evolving diameters were calculated to obtain the trajectories, and the diameters at the time the particles reached the receiver’s mask were used for the filtration efficiency and the lung deposition. Unless stated otherwise, the figures refer to the diameter at emission.

### Lung deposition model

The lung deposition of contaminated particles was based on the NCRP (National Council on Radiation Protection and Measurements) model, implemented using a compartments-in-series model^89^ based on standard lung data^90^. Detailed equations are given in section A11 in Supporting Information.

## Supplementary Information

Supplementary Information.
