# Supplementary material for: Quantitative modeling of the impact of facemasks and associated leakage on the airborne transmission of SARS-CoV-2
Source: Sci Rep. 2021 Sep 30;11:19403. doi: 10.1038/s41598-021-98895-9 (PMC8484595; doi:10.1038/s41598-021-98895-9)
Supplement: Supplementary file 1 — Supplementary Information. [file 41598_2021_98895_MOESM1_ESM.docx]

**Quantitative modeling of the impact of facemasks and associated leakage on the airborne transmission of SARS-CoV-2 – Supporting Information**

Jean Schmitt and Jing Wang

[A. Theoretical background and initial data 2](#_Toc81320425)

[A1. Calculation of the emitted particles size distributions 2](#_Toc81320426)

[A2. Calculation of the filtration efficiency of facemasks 4](#_Toc81320427)

[A3. Reconstruction of the facemasks’ filtration curves 7](#_Toc81320428)

[A4. Calculation of the leaking fraction as a function of the pressure drop 10](#_Toc81320429)

[A5. Distribution of the emitted particles between the leaking flow and the mask flow 11](#_Toc81320430)

[A6. Estimation of the leaking fraction from the fit factor 12](#_Toc81320431)

[A7. Definition of the leakage scenarios 13](#_Toc81320432)

[A8. Trajectories of the emitted droplets 13](#_Toc81320433)

[A9. Accumulation of viral charge in a closed volume 16](#_Toc81320434)

[A10. Calculation of the inhaled viral charge 18](#_Toc81320435)

[A11. Lung deposition model 18](#_Toc81320436)

[A12. Calculation of the viral charge 22](#_Toc81320437)

[B. Implementation 22](#_Toc81320438)

[B1. Interaction between the emitted airflow and the emitter’s mask 22](#_Toc81320439)

[B2. Trajectories and evaporation 23](#_Toc81320440)

[B3. Accumulation of viral charges around the emitter 26](#_Toc81320441)

[B4. Filtration performances of the receiver’s facemask 27](#_Toc81320442)

[B5. Lung deposition model 28](#_Toc81320443)

[C. Sensitivity assessment 28](#_Toc81320444)

[D. Model validation and walkthrough 33](#_Toc81320445)

[D1 Validation 34](#_Toc81320446)

[D2 Walkthrough 34](#_Toc81320447)

[E. Additional data 39](#_Toc81320448)

[E1 Additional data and discussion on the protection efficiency of facemasks considering a no-leakage scenario 39](#_Toc81320449)

[E2 Discussion on the differences between the different types of FFP masks in the different leaking scenarios (complementary discussion to Figure 6) 44](#_Toc81320450)

[E3 Relative protection of facemasks for speaking, coughing, and sneezing (complementary discussion to Figure 6) 44](#_Toc81320451)

[E4 Detailed data for the comparison between the exposure level without leakage and with leakage according to the standards (comparison of data in figures 5 and 6) 46](#_Toc81320452)

[E5. Comparison of the reach-rate and mask filtration efficiency of a FFP2 mask considering leakages of 0% and 29% (Comparison Figures S38c and 6e) 47](#_Toc81320453)

[E6 Detailed data on the filtration of charged filters as a function of the face velocity 48](#_Toc81320454)

[F. References 49](#_Toc81320455)

1. Theoretical background and initial data

This part lists the equations used in the model. Their implementation in the program is described in part B. The numerical models used in this work and the main corresponding references are summarized in **Table S1**, the detailed documentation is provided in the respective paragraphs. The parameters used in the study are summarized in **Figure S1**.

| **Section** | **Title** | **References** |
| --- | --- | --- |
| A1 | Emitted particles size distributions | Breathing, Speaking, Coughing: [29]  Sneezing: [34]  Counts: [35], [29], [36], [37], [38], [39], [40], [41], [42], and [43]. |
| A2 | Filtration efficiency of facemasks | Filtration model for fibrous filters: [2], [3], [4], [5], [6] |
| A4 | Calculation of the leaking fraction | Equilibrium between pressure drop of the mask and the leakage: [12], [13] |
| A8.1 | Droplet nuclei size and evaporation rate | Evaporation of respiratory droplets: [1], [36], [20] |
| A8.2 | Airflow velocity in a turbulent round-jet model | Turbulent round jet model: [22], [44], [45], [46] |
| A8.3 | Turbulences | Continuous Random Walk: [20], [23] |
| A8.4 | Motion of the droplets in the airflow | Lagrangian Particle Model: [21] |
| A9 | Accumulation of particles in a closed volume | Box model: [25], [26] |
| A11 | Lung deposition model | NCRP lung deposition model: [27], [4], [47] |

**Table S1**: Summary of the models used in the different modules with the corresponding references


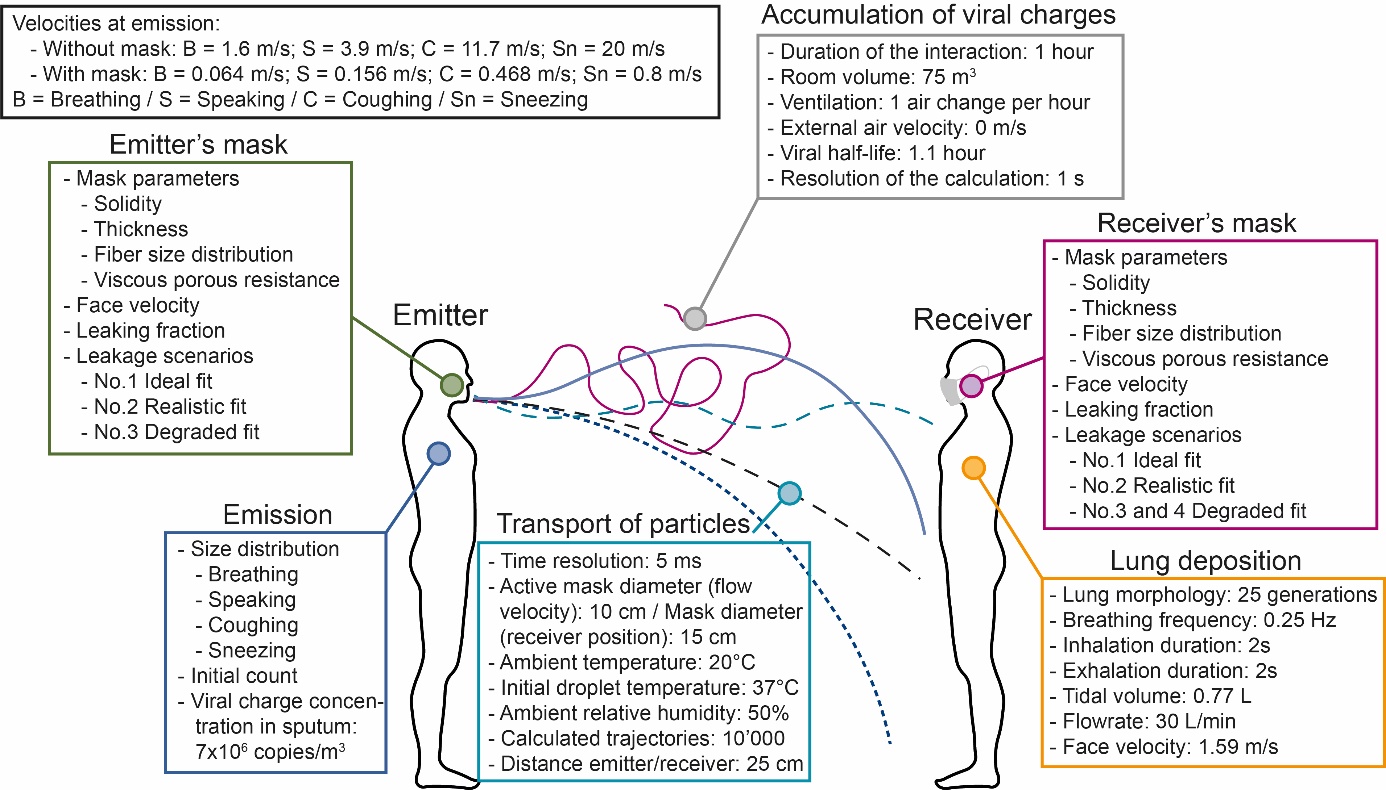


**Figure S1**: Summary of the parameters

A1. Calculation of the emitted particles size distributions

The emission size distribution for breathing, speaking, and coughing was calculated according to **Equation 1**, according to the Bronchiolar/Laryngeal/Oral (B.L.O.) model as proposed by [29], in reference to the sites of origin of the droplets generated during the different expiratory events. The model was proposed as a basis to epidemiological modeling of influenza epidemics. The parameter Cn represents the concentration, CMD the count median diameter, and GSD the geometric standard deviation. The corresponding numerical values given in **Table S2** were also taken from [29], and a correction factor was applied to compensate for the evaporation of the droplets, its value was taken from the calculation of the droplet nuclei size [1] used in the present work, and was set to 0.35.

$$\begin{aligned} \frac{dCn}{dLogD}=\ln\left( 10 \right)\cdot\sum_{i=1}^{3} \left[ \frac{Cn_{i}}{\sqrt{2\pi}\ln\left( GSD_{i} \right)}e^{-\frac{\ln\left( D \right)-\ln\left( CMD_{i} \right))^{2}}{2\ln(GSD_{i})^{2}}} \right]\#1 \end{aligned}$$

The size distribution for a sneeze is proposed by [34] and based on measurements carried out with a laser particle size analyzer immediately at the mouth to reduce the impact of droplet evaporation. **Equation 2.1** converts the volume-based distribution into a number-based distribution and Equations 2.2 and 2.3 taken from [34] are used to calculate the volume-based distribution.

$$\begin{aligned} P_{n}=\frac{max\left( P_{v,1};P_{v,2} \right)}{V_{droplet}}\#2.1 \end{aligned}$$

$$\begin{aligned} P_{v,i,1}=A_{1}\frac{1}{\sqrt{2\pi}\sigma_{1}}e^{-\frac{(log_{10}(D_{i})-\mu_{1})^{2}}{2\sigma_{1}^{2}}}\#2.2 \end{aligned}$$

$$\begin{aligned} P_{v,i,2}=A_{2}\frac{1}{\sqrt{2\pi}\sigma_{2}}e^{-\frac{(log_{10}(D_{i})-\mu_{2})^{2}}{2\sigma_{2}^{2}}}\#2.3 \end{aligned}$$

The values of the coefficients $A_{i}$, $\sigma_{i}$, and $\mu_{i}$ are given in **Table S3** and were also taken from [34]. The emission number corresponding to the different expiratory activities are given in **Table S4**. The average values in **Table S4** were considered for the normalization of the emission counts. The particles counts were compiled from [35], [29], [36], [37], [38], [39], [40], [41], [42], and [43].

| **Parameter** | **Breath** | **Speak** | **Cough** |
| --- | --- | --- | --- |
| Cn_1_ [cm^-3^] | 0.049 | 0.054 | 0.09 |
| Cn_2_ [cm^-3^] | N/A | 0.0684 | 0.1419 |
| Cn_3_ [cm^-3^] | N/A | 0.00126 | 0.01596 |
| GSD_1_ [-] | 2.3 | 1.3 | 1.25 |
| GSD_2_ [-] | N/A | 1.66 | 1.68 |
| GSD_3_ [-] | N/A | 1.795 | 1.837 |
| CMD_1_ [μm] | 2.29 | 2.31 | 2.24 |
| CMD_2_ [μm] | N/A | 3.43 | 2.29 |
| CMD_3_ [μm] | N/A | 145 | 123 |
| **Table S2**: Parameters used to build the input size distribution for breathing, speaking, and coughing. The values for the parameters were taken from [29]. | | | |

Cn_i_ represents the particle concentration in cm^-3^, GSD_i_ is the geometric standard deviation, and CMD_i_ the count median diameter in μm. The resulting distributions are shown in **Figure 2** in the main text.

|  | **Coefficient A_i_** | **Average μ_i_** | **Standard deviation σ_i_** |
| --- | --- | --- | --- |
| **Mode 1** | 6.0464 | 2.6992 | 0.2104 |
| **Mode 2** | 1.2623 | 1.9034 | 0.2465 |
| **Table S3**: parameters used to build the input size distribution for sneezing. The values for the parameters were taken from [34] | | | |

| **Level** | **Breath** | **Speak** | **Cough** | **Sneeze** |
| --- | --- | --- | --- | --- |
| **Low** | 50 | 60 | 165 | 5000 |
| **Average** | 350 | 1000 | 10000 | 75000 |
| **High** | 5000 | 20000 | 150000 | 1000000 |
| **Top** | 35000 | 150000 | 517000 | 1604600 |
| **Table S4**: Particle count for each emission level and expiratory activity. The counts for the different levels were taken from [35], [29], [36], [37], [38], [39], [40], [41], [42], and [43] and fitted to a lognormal distribution. | | | | |

A2. Calculation of the filtration efficiency of facemasks

This section describes the method used to calculate the filtration efficiency of facemasks, considering both mechanical (inertial impaction, diffusion, interception, and interception of diffusing particles) and electrostatic filtration (considering neutral particles and charged fibers). The simplified model used in the present study has been well documented, and the corresponding equations were taken from [2], [3], [4], and [5], the resulting filtration curves are given **in Figure 3** of the main text. The flow conditions used in this work are compatible with the use of the equations.

The filtration by diffusion is dominant for small particles sizes. The single-fiber efficiency E_D_ is given by **Equation 3.1** under the following conditions: 0.05 < α < 0.2 ; 0.001 < U_0_ < 2 m/s and 0.1 < d_f_ < 50 μm. If the conditions are not met, the efficiency is described by **Equation 3.2**.

$$\begin{aligned} E_{D}=2.58\cdot\left( \frac{1-\alpha}{Ku} \right)^{1/3}\cdot Pe^{-2/3}\#3.1 \end{aligned}$$

$$\begin{aligned} E_{D}=2Pe^{-2/3}\#3.2 \end{aligned}$$

α is the solidity of the filter, Ku the Kuwabara hydrodynamic factor (**Equation 4**) taking into the influence of neighboring fibers, and Pe represents the Peclet number (**Equation 5**).

$$\begin{aligned} Ku=\left\{ \begin{matrix} -\frac{\ln\left( \alpha\right)}{2}-\frac{3}{4}+\alpha-\frac{\alpha^{2}}{4}+\frac{2\cdot\lambda_{0}}{d_{f}} & \text{if}d_{f}<2\cdot{10}^{-6} \\ -\frac{\ln\left( \alpha\right)}{2}-\frac{3}{4}+\alpha-\frac{\alpha^{2}}{4} & \text{if}\text{ }d_{f}\geq2\cdot{10}^{-6} \end{matrix} \right.\#4 \end{aligned}$$

$$\begin{aligned} Pe=\frac{d_{f}U_{0}}{D}\#5 \end{aligned}$$

D is the diffusion coefficient of the particles (**Equation 6.1**) based on the slip correction factor $C_{C}$ (**Equation 6.2**), d_f_ the fiber diameter and U_0_ the face velocity.

$$\begin{aligned} D=\frac{kTC_{C}}{3\pi\mu d_{p}}\#6.1 \end{aligned}$$

$$\begin{aligned} C_{C}=1+\frac{p_{0}\lambda_{0}}{pD_{p}}\left[ 2.34+1.053e^{-0.39\frac{pd_{p}}{p_{0}\lambda_{0}}} \right]\#6.2 \end{aligned}$$

k is the Boltzmann constant, T the temperature, μ the dynamic viscosity of air, and d_p_ the particle’s diameter.

The filtration efficiency by interception E_R_ is given by **Equation 7**.

$$\begin{aligned} E_{R}=\frac{\left( 1-\alpha\right)\left( \frac{d_{p}}{d_{f}} \right)^{2}}{Ku\cdot\left( 1+\frac{d_{p}}{d_{f}} \right)}\#7 \end{aligned}$$

The filtration efficiency from inertial impaction E_I_, dominant for larger particles, is given by **Equations 8.1** to **8.3**.

$$\begin{aligned} E_{I}=\frac{Stk\cdot J}{2\cdot Ku^{2}}\#8.1 \end{aligned}$$

$$\begin{aligned} Stk=\frac{\rho_{p}d_{p}^{2}C_{C}U_{0}}{18\eta d_{f}}\#8.2 \end{aligned}$$

$$\begin{aligned} J=\left\{ \begin{matrix} \left( 29.6-28\alpha^{0.62} \right)\left( \frac{d_{p}}{d_{f}} \right)^{2}-27.5\left( \frac{d_{p}}{d_{f}} \right)^{2.8} & \text{if}\text{ }\frac{d_{p}}{d_{f}}<0.4 \\ 2 & \text{if}\text{ }\frac{d_{p}}{d_{f}}\geq0.4 \end{matrix} \right.\#8.3 \end{aligned}$$

The efficiency from the interception of diffusing particles is given by E_DR_ in **Equation 9**.

$$\begin{aligned} E_{DR}=\frac{1.24R^{2/3}}{(Ku\cdot Pe)^{1/2}}\#9 \end{aligned}$$

The efficiency by electrostatic filtration, given in **Equations 10.1** and **10.2** was calculated considered neutral particles filtered by a charged filter. σ is the charge density of the fiber, μ is the viscosity of the air, $\epsilon_{0}$ is the vacuum permittivity, $\epsilon_{f}$ is the dielectric constant of the filter, and $\epsilon_{p}$ the permittivity of the particle. The values for the different parameters were taken from [5] and are valid within the scope of the deposition of particles on electrically charged fibers. The values for the charging density were taken from [6] and were measured on commercially available filters. These values are likely to vary depending on the type of mask, the environmental conditions or the usage of the masks [48].

$$\begin{aligned} E_{q}=\left( \frac{1-\alpha}{Ku} \right)^{2/5}\frac{\pi N_{DD}}{1+2\pi N_{DD}^{2/3}}\#10.1 \end{aligned}$$

$$\begin{aligned} N_{DD}=\frac{2C_{c}\sigma^{2}d_{p}^{2}}{3\mu\epsilon_{0}(1+\epsilon_{f})^{2}d_{f}U_{0}}\left( \frac{\epsilon_{p}-1}{\epsilon_{p}+2} \right)\#10.2 \end{aligned}$$

The total single-fiber efficiency corresponds to the sum of each component (**Equation 11.1**) and the corresponding penetration P and filtration efficiency E are given in **Equations 11.2** and **11.3**.

$$\begin{aligned} E_{f}=E_{D}+E_{R}+E_{I}+E_{DR}+E_{q}\#11.1 \end{aligned}$$

$$\begin{aligned} P=e^{\frac{-4\alpha E_{f}t}{\pi\left( 1-\alpha\right)d_{f}}}\#11.2 \end{aligned}$$

$$\begin{aligned} E=1-P\#11.3 \end{aligned}$$

t is the filter’s thickness and d_f_ the diameter of the fiber. The filters are usually composed of fibers with various sizes; therefore, the fiber sizes were considered to follow a normal distribution. The filtration efficiency $P_{i}$ is therefore calculated individually for every fiber size and weighed with the occurrence in the distribution $n_{i}$, and summed to calculate the total penetration $P_{total}$ following **Equation 12**.

$$\begin{aligned} P_{total}=\sum_{i=1}^{d_{max}} P_{i}\cdot n_{i}\#12 \end{aligned}$$

The filtration efficiency by interception, impaction, diffusion, interception of diffusing particles and electrostatic filtration, as well as the total filtration, is shown in **Figure S2**. The influence of the flow velocity is highlighted in **Figure S3**.

| 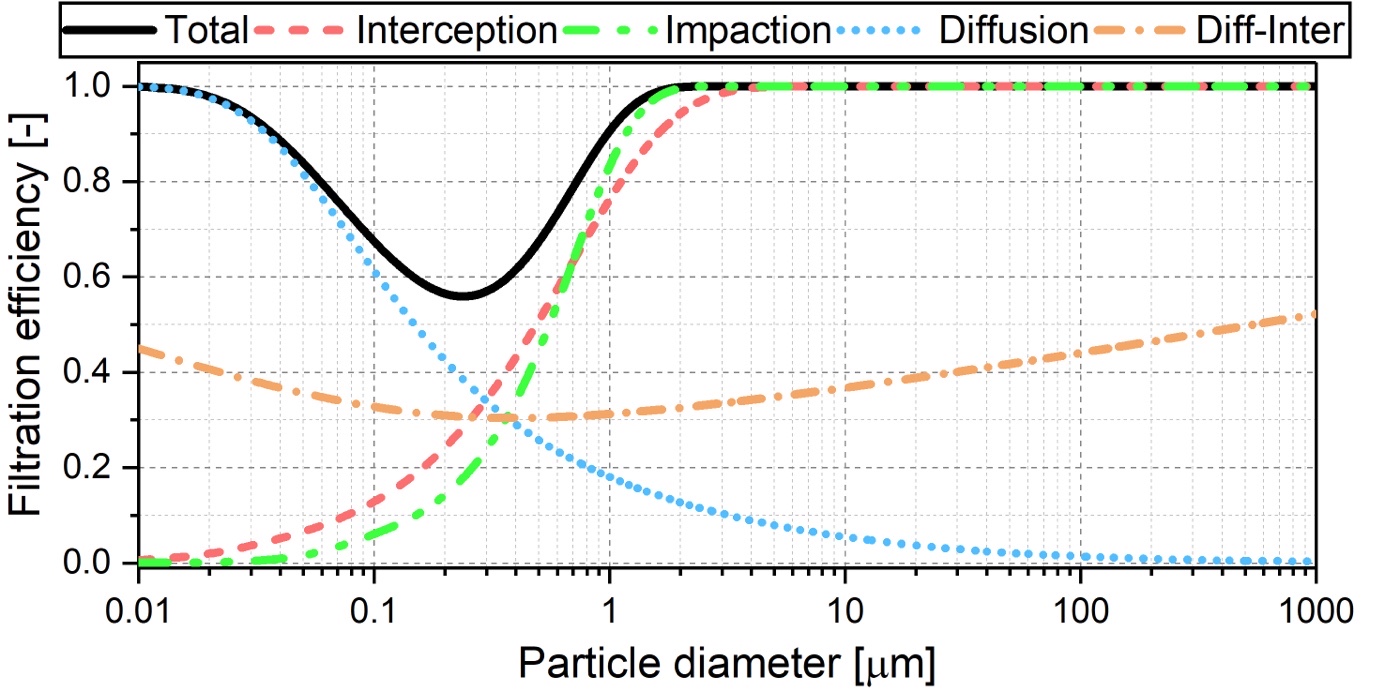  a |
| --- |
| 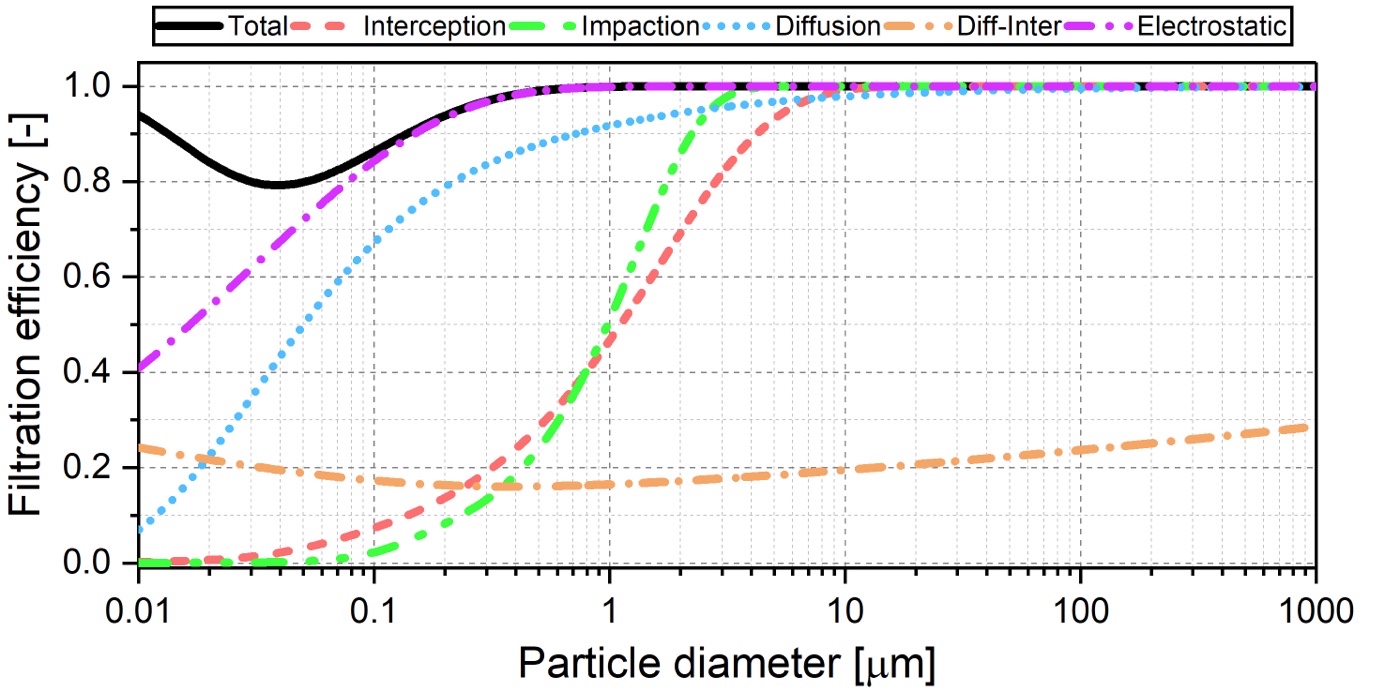  b |
| **Figure S2**: Filtration efficiency of a surgical mask as a function of the particle diameter. The contribution of the single filtration mechanisms considered in this work to the total filtration efficiency is given. A filter based only on mechanical filtration is shown in (a) and a filter based on mechanical and electrostatic filtration is shown in (b).  a   \| 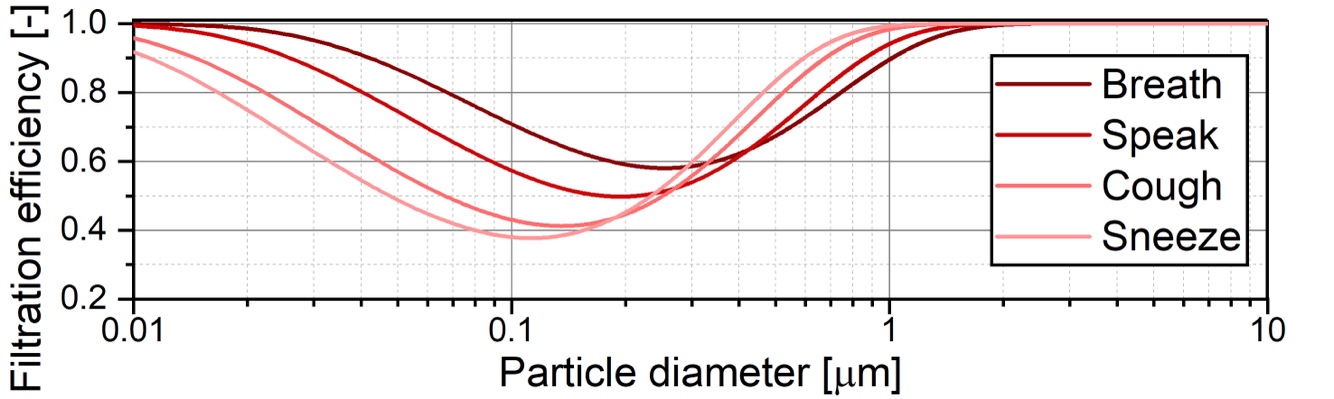  b \| \| --- \| \| 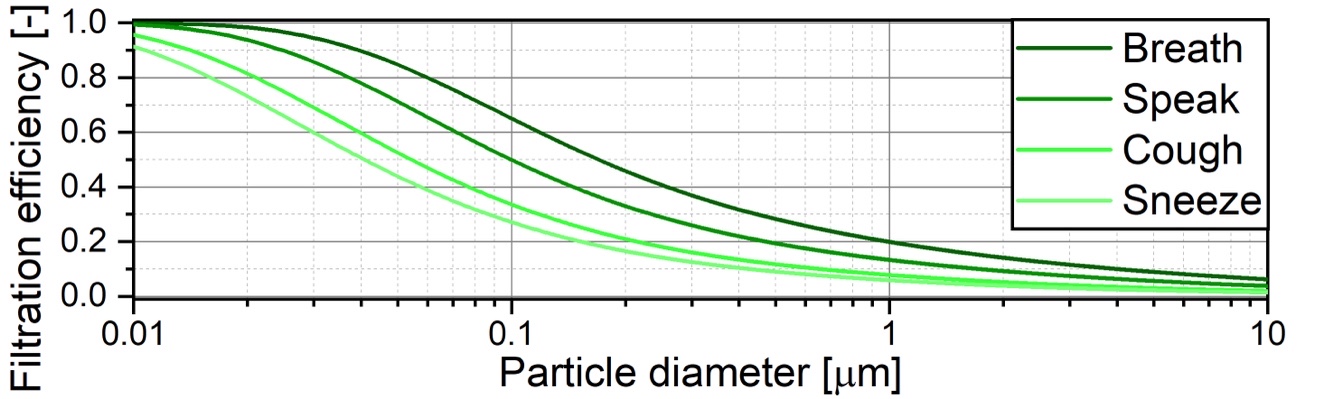 \| \|  \| \|  \| \| 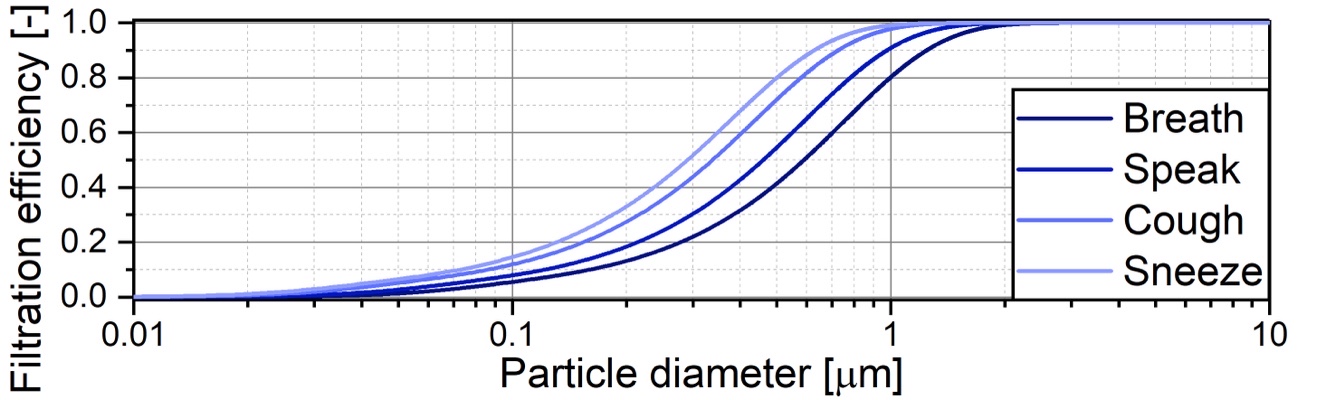  c  **Figure S3**: Evolution of the filtration efficiency of a surgical mask as a function of the face velocity, considering breathing, speaking, coughing, and sneezing. (a) complete filtration curve, (b) filtration based on diffusion, and (c) filtration based on inertial impaction. \| \| A3. Reconstruction of the facemasks’ filtration curves  The filtration efficiency and pressure drop of commercial facemasks was based on their respective standards and is summarized in **Table S5**.   \| **Mask type** \| **Standard** \| **Filtration efficiency** \| **Total inward leakage** \| **Pressure drop** \| **Flowrate (sample diameter)** \| \| --- \| --- \| --- \| --- \| --- \| --- \| \| FFP1 Inhalation \| EN149:2001  +A1:2009 \| 80% \| 25% \| 60 Pa/210 Pa \| 30 L/min/95 L/min (20 cm) \| \| FFP1 Exhalation \| 80% \| 25% \| 300 Pa \| 160 L/min (20 cm) \| \| FFP2 Inhalation \| 94% \| 11% \| 70 Pa / 240 Pa \| 30 L/min/95 L/min (20 cm) \| \| FFP2 Exhalation \| 94% \| 11% \| 300 Pa \| 160 L/min (20 cm) \| \| FFP3 Inhalation \| 99% \| 5% \| 100 Pa/300 Pa \| 30 L/min/95 L/min (20 cm) \| \| FFP3 Exhalation \| 99% \| 5% \| 300 Pa \| 160 L/min (20 cm) \| \| Medical Type I \| ﻿EN 14683:2019  +AC:2019 \| 95% \| 25%* \| 40 Pa/cm^2^ \| 8 L/min (25 mm) \| \| Medical Type II \| 98% \| 22%* \| 40 Pa/cm^2^ \| \| Medical Type IIR \| 98% \| 22%* \| 60 Pa/cm^2^ \| \| SNR 30000 \| SNR 30000:2021 \| 70% \| N/A \| 294 Pa \| Face velocity 0.27 m/s \| \| *: EN 14683:2019+AC:2019 only specifies the filtration properties of the material and not the total inward leakage; it is estimated at 25% \| \| \| \| \| \| \| **Table S5**: Requirements from commercial facemasks based on their respective standards \| \| \| \| \| \|   The EN 149 standard is based on the EN 13274-7 for the penetration through the filtering material and considers two tests: the NaCl test using an average particle diameter of 80 nm and the paraffin oil test using an average size of 370 nm. Both tests are conducted at 95 L/min on the complete facemask (20 cm), leading to an average face velocity of 5 cm/s. The paraffin test was used in the present work as a reference for the calculation of the filtration curve. The pressure drop data are based on measurements available in the literature [7] for FFP masks including electrostatic filtration: to 45 Pa for the FFP1, 60 Pa for the FFP2, and 65 Pa for the FFP3 mask, values measured at 5.3 cm/s.  The EN 14683 standard focuses on bacterial penetration and the average aerosol size is 3 μm. The test flow is 8 L/min through a 25 mm diameter sample. These assumptions led to a face velocity of 27.2 cm/s and a prescribed pressure drop of 196 Pa.  To reflect the variability in the filtration efficiencies of medical masks, additional measurements data from the literature [8] on two masks with different levels of filtration used in medical facilities (labelled as surgical high and surgical low) and two masks used in dental facilities (labelled as dental high and dental low) was considered. The filtration efficiency and pressure drops are summarized in **Table S6**.   \| **Type of mask** \| **Filtration efficiency** \| **Particle size** \| **Pressure drop** \| **References** \| \| --- \| --- \| --- \| --- \| --- \| \| **Surgical high** \| 96.04% / 99.96% \| 300 nm / 800 nm \| 54.4 at 5.3 cm/s \| [8] / [9] \| \| **Surgical low** \| 62.6% / 98.45% \| \| **Dental high** \| 47.1% / 85.3% \| EN 14683 \| \| **Dental low** \| 9.8% / 18.3% \| \| **Table S6**: Data used in the modelling of additional surgical and dental masks \| \| \| \| \|   Homemade facemasks are based on various materials. Their filtration characteristics and breathing resistance are not regulated, and these devices are not produced industrially. Their characteristics are therefore likely to show a high variability. The considered materials and their respective interpolated filtration data are given in **Table S7**.   \| **Material** \| **Minimum filtration efficiency*** \| **Most penetrating particle size*** \| **Pressure drop**** \| **References** \| \| --- \| --- \| --- \| --- \| --- \| \| **Jersey** \| 31.5% \| 270 nm \| 103 Pa \| [9] \| \| **Cotton pillowcase** \| 15% \| 230 nm \| 4.5 Pa \| [10], [11] \| \| **Cotton t-shirt** \| 17% \| 230 nm \| 14.5 Pa \| [10], [11] \| \| **Silk** \| 11% \| 220 nm \| 33.7 Pa \| [9] \| \| **Vacuum bag** \| 90% \| 200 nm \| 99.6 Pa \| [9] \| \| **Poly velvet** \| 35% \| 410 nm \| 70 Pa \| [9] \| \| * At 5 cm/s face velocity / ** The pressure drop is based on data from the literature and recalculated for a 20 cm diameter sample at 5 cm/s face velocity \| \| \| \| \| \| **Table S7**: Parameters used for the interpolation of the filtration curves of homemade masks based on various materials \| \| \| \| \|   The datapoints used to reconstruct the filtration curves of the facemasks are given in **Table S8**.   \| **Material** \| **30 nm** \| **50 nm** \| **100 nm** \| **250 nm** \| **500 nm** \| **1 μm** \| **2.5 μm** \| **5 μm** \| **10 μm** \| \| --- \| --- \| --- \| --- \| --- \| --- \| --- \| --- \| --- \| --- \| \| **Jersey** \| 70.5% \| 60.5% \| 45.5% \| 31.5% \| 31.5% \| 58.4% \| 87.3% \| 98.4% \| 100% \| \| **Silk** \| 35.5% \| 27.5% \| 15.9% \| 15.6% \| 16.3% \| 30.4% \| 56.9% \| 84.1% \| 92.4% \| \| **Vacuum bag** \| 96.4% \| 94.4% \| 93.8% \| 95.9% \| 98.5% \| 100% \| 100% \| 100% \| 100% \| \| **Poly velvet** \| 87.2% \| 77.5% \| 57.3% \| 33% \| 31.2% \| 48.6% \| 78.1% \| 100% \| 100% \| \| **Table S8.1**: Datapoints used to reconstruct the filtration efficiency curves of materials for homemade masks. Data from [9] based on a face velocity of 5.3 cm/s. \| \| \| \| \| \| \| \| \| \|  \| **Material** \| **75 nm (NaCl)^1^** \| **23 nm (phage)^2^** \| **1000 nm (bacteria)^2^** \| \| --- \| --- \| --- \| --- \| \| **Cotton pillow cover** \| 5.04% \| 69.42% \| 50.85% \| \| **Cotton t-shirt** \| 21.62% \| 61.28% \| 57.13% \| \| **Table S8.2**: Datapoints used to reconstruct the filtration efficiency curves of cotton-based filters. ^1^Data from [11] based on a flowrate of 82 L/min; ^2^data from [10] based on a flowrate of 30 L/min. \| \| \| \|  \| **Masks** \| **300 nm** \| **800 nm** \| **2000 nm** \| **3100 nm** \| \| --- \| --- \| --- \| --- \| --- \| \| **Surgical high** \| 96.04% \| 99.96% \| 99.994% \| 99.98% \| \| **Surgical low** \| 62.6% \| 98.45% \| 99.72% \| 99.9% \| \| **Dental high** \| 47.1% \| 85.3% \| 95.82% \| 99.38% \| \| **Dental low** \| 9.8% \| 18.3% \| 22.8% \| 32.6% \| \| **Table S8.3**: Datapoints used to reconstruct the filtration efficiency curves of additional surgical and dental masks. Data from [8] considering a flowrate of 6 L/min on the complete mask. \| \| \| \| \|   Virtual facemasks simulate the protection of a device based on different requirements for filtration efficiency at a defined particle diameter. The simulated masks were based on a filtration efficiency of 70% at 1 μm, 3μm, and 5μm, and two more masks were based on 90% efficiency at 1 μm and 3 μm. The efficiency was calculated for a face velocity of 8 cm/s, and the pressure drop was set to 294 Pa at 0.27 m/s, following the guidelines from the standard SNR 30000:2021.  The datapoints from the literature are fitted using the equations describing the filtration efficiency. FFP masks were based on the lowest filtration efficiency allowed by the EN 149 standard. The charge density of the fibers was taken from [6]. FFP1, FFP2, and FFP3 masks were modelled with mechanical and electrostatic filtration. Additional FFP2 masks were modelled to take into account the influence of the pressure drop and the MPPS on the protection efficiency:   - FFP2 Mech.: based on mechanical filtration only with the condition of the lowest filtration efficiency at 350 nm - FFP2 Charges loss: had the same mechanical parameters (solidity, thickness, and fiber size distribution) than FFP2-2 but the electrostatic charge of the fibers was set to zero to simulate a discharge of the mask   The pressure drops of the FP1, FFP2, and FFP3 masks was taken from the literature [7]. The mechanical FFP2 mask was modeled with the highest pressure drop allowed by the EN 149 standard. \| \|  \| |

A4. Calculation of the leaking fraction as a function of the pressure drop

The leaking flow is calculated as a function of the equilibrium of the pressure drops generated by both the facemask and the gap. A simplified analytical method to calculate the flow distribution between, the leaking flow and the mask flow as a function of the viscous porous resistance of the mask and the dimensions of the gap is proposed by [12], summarized in this section, and integrated into the model. The method is based on the assumption of an incompressible fluid and a uniform distribution of the pressure within the facemask. We considered a simplified geometry with an equivalent rectangular gap between the wearer’s face and the mask accounting for the different gaps that might appear in a real usage case. The flow $Q$ through a rectangular gap as a function of the pressure drop$\Delta P$ can be approximated by **Equation 13** for flat gaps ($w>10 h$) [13], with $h^{3}$ representing the height of the gap (distance between the wearer’s face and the mask), $w$ is its width, $L$ its length and $\eta$ the air viscosity.

$$\begin{aligned} Q=\frac{h^{3}w}{12\eta L}\Delta P\#13 \end{aligned}$$

The velocity of the flow through the gap $u_{g}$ is given by **Equation 14**, $S_{g}=w\cdot h$ is the gap’s cross section.

$$\begin{aligned} u_{g}=\frac{Q}{S_{g}}\#\#14\# \end{aligned}$$

Combining Equations 12 and 13 gives the pressure drop through the gap as a function of the velocity (**Equation 15**).

$$\begin{aligned} \Delta P_{gap}=\frac{12\eta L}{h^{2}}u_{g}\#15 \end{aligned}$$

The pressure drop at the gap’s inlet and outlet is given by **Equation 16**, considering the loss coefficient $\xi$=1.5.

$$\begin{aligned} \Delta P_{gap,i,o}=\frac{u_{t}}{\left| u_{t} \right|}\xi\frac{\rho}{2}u_{g}^{2}\#16 \end{aligned}$$

The pressure drop$\Delta P_{mask}$ generated by the facemask can be approximated as given in **Equation 17**, as a function of the viscous porous resistance $C_{m}$, the volumetric mass of the fluid $\rho$ and flow velocity through the mask $u_{m}$.

$$\begin{aligned} \Delta P_{mask}=C_{m}\rho u_{m}\#17 \end{aligned}$$

**Equations 18.1** to **18.4** describes the velocity through the leakage, injected into Equation 14 to calculate the volumetric flow through the gap.

$$\begin{aligned} u_{g}=\frac{-b+\sqrt{b^{2}-4ac}}{2a}\#18.1 \end{aligned}$$

$$\begin{aligned} a=\frac{u_{t}}{\left| u_{t} \right|}\frac{\xi\rho}{2}\#18.2 \end{aligned}$$

$$\begin{aligned} b=\frac{12\mu L}{h^{2}}+\frac{C_{m}\rho hw}{S_{m}}\#18.3 \end{aligned}$$

$$\begin{aligned} c=-\frac{C_{m}\rho F_{t}}{S_{m}}\#18.4 \end{aligned}$$

The leaking flow was calculated as $F_{g}=u_{g}hw$ and the leaking fraction is the ratio $\frac{F_{g}}{F_{t}}$, based on the total emitted flow $F_{t}$.

The outward and inward leakage as a function of the height of the gap between the mask and the wearer’s face is given in **Figure S4**. The width and length are constant (respectively 10 cm and 5 mm).

| 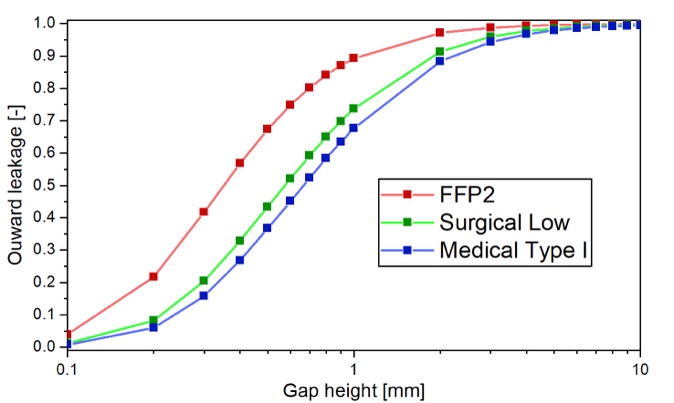 | 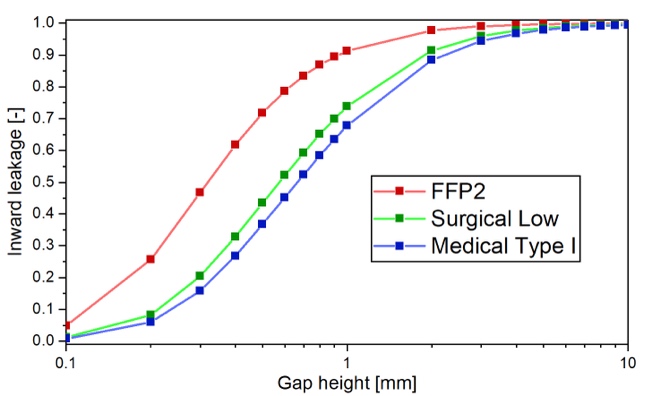 |
| --- | --- |
| **Figure S4**: Outward leakage (a) and inward leakage (b) as a function of the height of the gap between the mask and the wearer’s face. The length (10 cm) and width (5 mm) are constant. In the model, the width was kept constant while the height and length were adapted to reach the desired leaking flow.  A5. Distribution of the emitted particles between the leaking flow and the mask flow  The leaking flow changed its direction compared to the initial flow, and a fraction of the emitted droplets were not able to follow the direction change. These particles stayed in the mask flow where they were filtered. This fraction was estimated considering an impactor whose cutoff size depends on the initial flow velocity (or inhalation velocity for the receiver) and the mouth diameter. The equations 19 and 20, describing the behavior of the particles passing through an inertial impactor are given by [4]. We chose not to consider a virtual impactor, as the minor flow (mask flow in the present model) would be limited to 5-10% of the total flow [4]. The inertial impactor model allows for more flexibility. We considered that the facemask acted like a non-penetrable plate for the leaking flow (while it was penetrated by the mask flow, treated separately). The Stokes number as a function of the particle size $d_{p}$ (taken as the size at emission for the emitter’s mask or the size upon impact on the receiver’s mask) is given in **Equation 19**. $C_{C}$ represents the slip correction factor, whose calculation is detailed in **Supporting Information**.  $\begin{aligned} Stk=\frac{\rho_{p}d_{p}^{2}U_{0}C_{C}}{9\eta D_{init}}\#19 \end{aligned}$  The transfer function of the virtual impactor was modelled by a stepwise function as described in **Equation 20**. The Stokes number corresponding to a 50% collection efficiency is given by [4] for a circular jet: $Stk_{50}=0.24$.  $\begin{aligned} \text{Leaking fraction}=\left\{ \begin{matrix} 1 & \text{if}\text{ }Stk<Stk_{50} \\ 0.5 & \text{if}\text{ }Stk=Stk_{50} \\ 0 & \text{if}\text{ }Stk>Stk_{50} \\ & \end{matrix} \right.\#20 \end{aligned}$  Particles with a diameter smaller than the cut-off size corresponding to Stk = St_k50_ followed the leaking flow, while larger particles stay in the mask flow. The concept is illustrated in **Figure S5**, with the decision tree for the particles in **Figure S5a** for the emitter, **Figure S5b** for the receiver. The considered flow velocities are given in **Figure S5c** for the emitter and **Figure S5d** for the receiver. The penetrating fraction through a surgical mask considering different levels of leakages is given in **Figure S5e** for the emitter and **Figure S5f** for the receiver.   \| 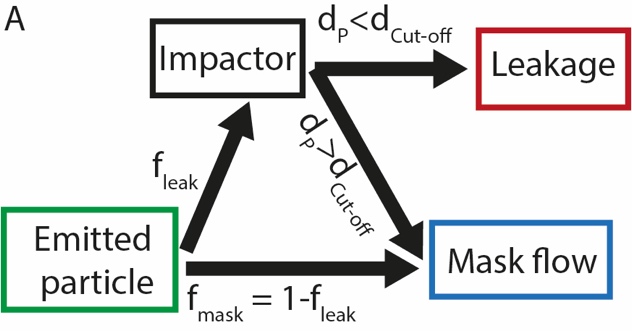 \| 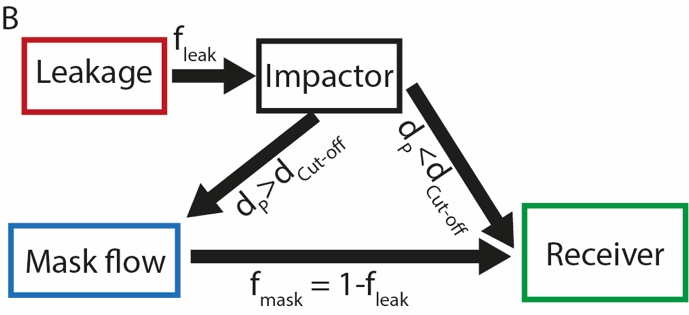 \| \| \| --- \| --- \| --- \| \| 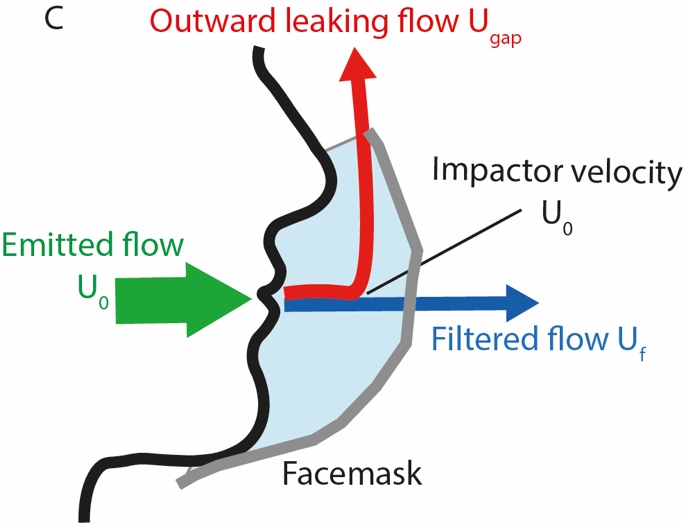 \| 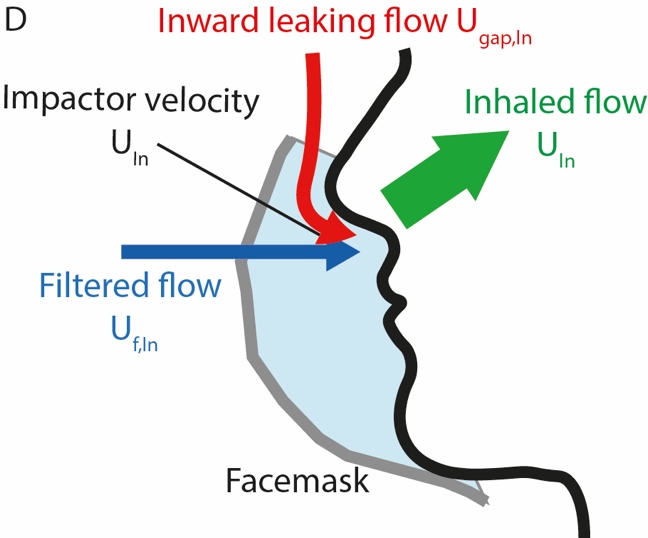 \| \| \| 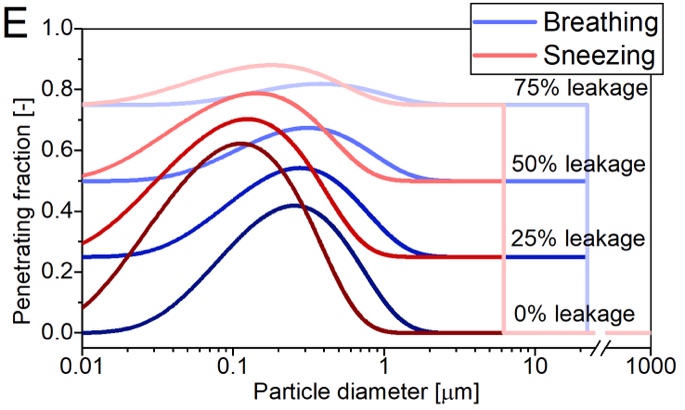 \| \| 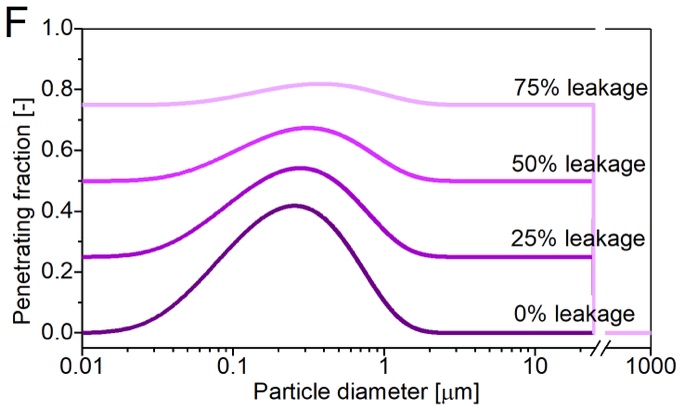 \| \| **Figure S5**: Illustration of the model considered for the leaking flow and the mask flow. The distribution of the emitted particles between the mask flow and the leaking flow is shown in (A) for the emitter and (B) for the receiver. *f_leak_* represents the leaking flow as a fraction of the total emitted flow, and d_Cut-off_ is the cut-off size of the impactor. Particles larger than the cut-off size are removed from the leaking flow and move with the emitted mask flow. The considered flow velocities are given in (C) for the emitter and (D) for the receiver. The emitter’s initial emission velocity U_0_ and the mouth diameter are used to calculate the cut-off size. The leaking flow is released after passing through the gap with the corresponding velocity U_gap_ , and the mask flow is released at the face velocity U_f_. The face velocity of the flow interacting with the receiver’s mask is calculated from the inhalation face velocity U_f,in_ calculated from the inhalation flow (30 L/min flowrate, leading to a flow U_in_). The fraction of particles removed from the inward leaking flow is calculated considering the receiver’s mouth (or nose) diameter and the inhaled flow. The leaking flow enters the mask at a velocity U_gap_. The penetration as a function of the particle’s diameter is shown in (E) for the emitter and (F) for the receiver. The calculation is based on a surgical mask and on the initial velocity of breathing and sneezing for the emitter. The curve for the emitter is calculated considering U_in_ at 30 L/min. The diameters are equal to the diameter at emission for the emitter’s mask and to the diameter after evaporation for the receiver’s mask.  A6. Estimation of the leaking fraction from the fit factor  The leaking fraction was estimated from the fit factor using **Equation 21.2**, where the mask’s filtration efficiency was approximated with a coefficient e$ff_{mask}$, taken as the minimum efficiency of the mask (e.g., 94% for a FFP2 mask). For simplification purposes, the inward leaking flow was considered to carry the same particle concentration as the ambient air. Combining 21.1 and 21.2 leads to **Equation 21.3**. $f_{mask}$is the fraction of the volumetric flow entering through the mask, and the leaking fraction is ${1-f}_{mask}$.  $\begin{aligned} \text{fit factor}=\frac{C_{out}}{C_{in}}\#21.1 \end{aligned}$  $\begin{aligned} C_{in}=\left( f_{mask}\cdot\left( 1-eff_{mask} \right)+\left( 1-f_{mask} \right) \right)C_{out}\#21.2 \end{aligned}$  $\begin{aligned} f_{mask}=\frac{1}{eff_{mask}}\left( 1-\frac{1}{\text{fit}\text{ }\text{factor}} \right)\#21.3 \end{aligned}$ \| \| \| | |

A7. Definition of the leakage scenarios

- **Fit according to the standards**: the lower limit of the compliance to the standards was simulated. The level of leakage was derived from the data in **Table S5** and summarized in **Table S9**. The outward leakage was considered to be equal to the inward leakage.

| **Mask** | **Total inward leakage (mask + leakage)*** | **Maximum penetration (mask only)** | **Calculated leaking fraction** |
| --- | --- | --- | --- |
| **FFP1** | 25% | 20% | 5% |
| **FFP2** | 11% | 6% | 5% |
| **FFP3** | 5% | 1% | 4% |
| **Medical Type I** | 25%** | 5% | 20% |
| **Surgical** |  |  |  |
| * The outward leakage is considered equal to the inward leakage  **Considered to be similar to a FFP1 mask, the EN 14683 standard does not indicate a level of leakage. | | | |
| **Table S9**: Levels of leakage considered in the fit according to the standards | | | |

- **Fit based on values reported in the literature**. Measurements of the fit factors on experienced and trained individuals show a high variability: [14] reported fit factors ranging from 5 to >5000 for FFP3 facemasks, [15] measured an average fit factor of 20.5 for FFP2 masks (ranging from 55 to 3) and a fit factor of 2.6 for surgical masks (ranging from 3.3 to 1.2). [16] estimated the protection factor for homemade masks (2.5 for adults, 2.1 for children), surgical masks (4.7 for adults and 3.8 for children) and FFP2 protections (90 for adults and 20 for children). [17] measured the fit factor of N95 respirators worn by trained users performing seal checks. The average fit factor was 141 with a minimum at 66 and a maximum at 200. After one hour exercise, the average fit factor dropped to 75 with extreme values of 22 and 146. [18] measured the total inward leakage and found an average of 8.6% for FFP2 masks (5^th^ and 95^th^ percentiles at 0.3% and 35%) and 32% (5%-82.5%) for surgical masks. [19] measured the fraction of particles penetrating from leakages. A maximum leaking penetration of 5% for 100 nm particles was measured for FFP2 masks, while this value increased to 37% for surgical masks.

The lowest fit factors found in the literature were used for this scenario: 3 for the FFP masks, and 1.2 for the surgical and homemade masks and converted into leaking fraction following the method described in the previous section. The data from the literature highlights the ability of FFP masks to better fit on the wearer’s head, which was also considered in scenario F3.

A8. Trajectories of the emitted droplets

The trajectories of single droplets are computed, resulting in the calculation of the fraction of the emitted particles reaching the position of the receiver’s face. The module dedicated to the calculation of the trajectories considers the influence of the evaporation detailed in section A8.1 and leading to the calculation of the dynamic diameter of the droplets, from their emission with the corresponding initial diameter between 200 nm and 1 mm to their dried state at the droplet nuclei size. The equations leading to the calculation of the diameter of the drying particles are proposed by [1]. The complete turbulent steady cough jet model based on a Lagrangian particle model has been proposed by [21] to investigate trajectories of respiratory droplets. A modification of the calculation of the turbulences was proposed by [20], with the introduction of a continuous random walk model to simulate the turbulent fluctuating velocity. The model for the cough jet is well established and has been widely described ([22], [44], [45], [46]).

Different expiratory activities are described by adapting the initial velocity. We used a simplified model to describe the influence of the facemasks on the emitted jet: the initial velocity as well as the initial diameter of the jet were calculated from the mask’s surface. We did not consider the additional turbulences resulting from the porous media.

**A8.1 Droplet nuclei size and evaporation rate**

Droplets are composed of a liquid fraction composed of water, and a solid fraction mostly composed of sodium chloride, dead cells, proteins, pulmonary surfactant, bacteria and viruses. The calculation of the dry nuclei size is given by [1], we considered the same initial sodium chloride concentration in the droplets (150 mM). [1] takes the initial solid volume ratio as 1.8%, according to [36] who investigated the emission and drying of expiratory droplets ejected during various expiratory events. The dry diameter is given by **Equation 22**.

$$\begin{aligned} d_{dry}=\left( \frac{\Phi_{i}^{*}}{\Phi_{max}} \right)^{1/3}d_{i}\#22.1 \end{aligned}$$

$$\begin{aligned} \Phi^{*}=\Phi_{i}+\frac{\rho_{i}\cdot c_{i}}{\rho_{NaCl}}\#22.2 \end{aligned}$$

$d_{dry}$ is the nuclei diameter, $\Phi_{i}$ is the initial solid volume ratio, $\rho_{i}$ is the average initial density of the droplet, $\rho_{NaCl}$ the density of the sodium chloride, $c_{i}$ is the initial sodium chloride concentration in the droplet and $\Phi_{max}=0.5236$ [1] is a correction factor to account for the formation of porosities in the solid fraction during the evaporation process and the crystallization of the salt.

The evaporation rate and final diameter depend on the ambient humidity. Taking into account the hydrophilicity of the solid fraction, a certain amount of water can stay in the droplet at high relative humidity, above the threshold humidity (TRH) calculated in **Equation 23**.

$$\begin{aligned} TRH=e^{\frac{4M_{W}\sigma_{LV}}{\rho_{W}RT_{\infty}d_{i}}\cdot\frac{\Phi_{max}}{\Phi_{i}}-\frac{\nu\Psi_{salt}M_{W}c_{i}\left( 1-\Phi_{i} \right)}{M_{salt}\left( 1-c_{i} \right)}\cdot\frac{\Phi_{max}}{\Phi_{i}-\Phi_{i}\cdot\Phi_{max}}}\#23 \end{aligned}$$

If the ambient humidity is lower than the TRH value, the droplet will evaporate into its droplet nuclei size. If it is higher than the TRH, the final size is given by **Equation 24**.

$$\begin{aligned} ln\left( RH \right)=\frac{4M_{W}\sigma_{LV}}{\rho_{W}RT_{\infty}d_{e}}-\frac{\nu\Psi_{salt}M_{W}c_{i}\left( 1-\Phi_{i} \right)}{M_{salt}\left( 1-c_{i} \right)}\cdot\frac{1}{(\frac{d_{e}}{d_{i}})^{3}-\Phi_{i}}\#24 \end{aligned}$$

The evaporation rate is calculated as a change of the water mass as a function of time and converted into a change in diameter. The evaporation rate is given in **Equation 25**.

$$\begin{aligned} \frac{dm_{d}}{dt}=\frac{2\pi pd_{s}M_{W}D_{\infty}C_{T}Sh}{RT_{\infty}}ln\left( \frac{p-p_{s}}{p-p_{\infty}} \right)\#25 \end{aligned}$$

$C_{T}$is a temperature correction coefficient $C_{T}=\frac{T_{\infty}-T_{p}}{T_{\infty}^{\lambda-1}}\cdot\frac{2-\lambda}{T_{\infty}^{2-\lambda}-T_{p}^{2-\lambda}}$, $Sh$ is the Sherwood number $Sh=1+0.3Re^{\frac{1}{2}}Sc^{\frac{1}{3}}$ and $Sc$ the Schmidt number $Sc=\frac{\nu}{D_{\infty}}$. The droplet temperature is given by [20] in **Equation 26**.

$$\begin{aligned} c_{p}m_{p}\frac{dT_{p}}{dt}=2\pi d_{p}K_{g}\left( T_{\infty}-T_{p} \right)Nu-L_{v}\cdot I_{v}-\pi d_{p}^{2}\Gamma\left( T_{p}^{4}-t_{\infty}^{4} \right)\#26 \end{aligned}$$

$Nu$ is the Nusselt number $Nu=1+0.3Re^{\frac{1}{2}}Pr^{\frac{1}{3}}$ and $Pr$ the Prandtl number $Pr=\frac{c\mu}{K_{g}}$ .

**A8.2 Airflow velocity in a turbulent round jet**

The equations describing the air velocity and the motion of the droplets were taken from [20], [1], [21], and [22]. This model was developed to simulate a cough, but was also used in the present work to approximate the different situations: breathing, speaking, coughing and sneezing, considering adapted initial parameters for each situation. The flow can be divided into two zones:

- The zone of flow establishment where the stream velocity is constant and equal to the initial velocity, the radial velocity is negligible and no turbulences occur. The length of the zone is defined as $6.2\cdot D_{m}$ where $D_{m}$is the mouth diameter.
- The zone of established flow, where the streamwise velocity has a gaussian distribution in the jet’s cross section and is given by **Equations 27** and **28**.

The air velocity $U_{C}$ along the centerline of the jet is given by **Equation 27**, with x representing the distance from the emission point.

$$\begin{aligned} U_{C}=6.2\cdot U_{0}\frac{D_{m}}{x}\#27 \end{aligned}$$

In order to consider the limited duration of the jet emission for the cough and sneeze, the centerline velocity is multiplied by a correction factor $e^{-\frac{t}{\tau}}$, where the time constant τ depends on the expiratory activity. The streamwise velocity follows a gaussian distribution and is given by **Equation 28**. It depends on the radial distance$r$ from the centerline and the Gaussian half-width $b_{g}=\beta x$ with $\beta=0.114$ represents the jet width growth rate.

$$\begin{aligned} U_{r}=U_{c}\cdot e^{-\frac{r^{2}}{b_{g}^{2}}}\#28 \end{aligned}$$

The radial velocity is given by **Equation 29**, with the jet entrainment coefficient $\alpha=0.057$.

$$\begin{aligned} V_{r}=\alpha\cdot U_{C}\frac{1-e^{-\frac{r^{2}}{b_{g}^{2}}}-\frac{\beta}{\alpha}\frac{r^{2}}{b_{g}^{2}}\cdot e^{-\frac{r^{2}}{b_{g}^{2}}}}{\frac{r}{b_{g}}}\#29 \end{aligned}$$

**A8.3 Turbulences**

Turbulences were modeled using a continuous random walk (CRW) method as described by [20] in a similar particle tracking application as an alternative to the Discrete Random Walk (DRW) model presented by [21]. The equations are given by [23] and applied by [20] to the round jet model. It is based on the turbulent kinetic energy (**Equation 30**) and the dissipation rate (**Equation 31**).

$$\begin{aligned} k=U_{c}^{2}\cdot c_{1}\left( e^{-c_{2}\left( \frac{r}{b}-c_{3} \right)^{2}}+e^{-c_{2}\left( \frac{r}{b}+c_{3} \right)^{2}} \right)\#30 \end{aligned}$$

$$\begin{aligned} \epsilon=\frac{U_{c}^{3}}{b}\cdot c_{4}\left( e^{-c_{5}\left( \frac{r}{b}-c_{6} \right)^{2}}+e^{-c_{5}\left( \frac{r}{b}+c_{6} \right)^{2}} \right)\#31 \end{aligned}$$

With the coefficient $c_{1}=0.0667$, $c_{2}=1.079$, $c_{3}=0.6853$, $c_{4}=0.0178$, $c_{5}=1.963$, and $c_{6}=0.6126$. The lagrangian time scale is described in **Equation 32** and the mean velocity fluctuation is given by **Equation 33**, considering $C_{\mu}=0.09$.

$$\begin{aligned} \tau_{i}=\sqrt{\frac{3}{2}}C_{\mu}^{\frac{3}{4}}\frac{k}{\epsilon}\#32 \end{aligned}$$

$$\begin{aligned} \sigma=\sqrt{\frac{2k}{3}}\#33 \end{aligned}$$

[20] uses a discrete form (**Equation 34.4**) of the Langevin equation (**Equation 34.1**) to calculate the turbulent contribution to the air velocity $u_{i}^{'}$, proposed by [24].

$$\begin{aligned} \frac{du_{i}^{'}}{dt}=-\alpha u_{i}^{'}+\beta\xi_{i}\#34.1 \end{aligned}$$

$$\begin{aligned} \alpha=\frac{1}{\tau_{i}}\#34.2 \end{aligned}$$

$$\begin{aligned} \beta=\sigma_{i}\left( \frac{2}{\tau_{i}} \right)^{\frac{1}{2}}\#34.3 \end{aligned}$$

$$\begin{aligned} u_{i}^{'}\left( t+\Delta t \right)=u_{i}^{'}\left( t \right)e^{-\frac{\Delta t}{\tau_{i}}}+\sigma_{i}\left( 1-e^{-2\frac{\Delta t}{\tau_{i}}} \right)^{\frac{1}{2}}\xi_{i}\#34.4 \end{aligned}$$

$\Delta t$ is the time step of the simulation and $\xi_{i}$ Gaussian white noise. The turbulent component was generated for all three flow directions.

**A8.4 Motion of droplets in the air flow**

The lagrangian equations describing the motion of droplets in the airflow is given by **Equations 35.1** and **35.2**, taken from [21].

$$\begin{aligned} \frac{dx_{i}}{dt}=u_{p,i}\#35.1 \end{aligned}$$

$$\begin{aligned} \frac{du_{p,i}}{dt}=\frac{3\rho_{g}C_{d}}{4d_{p}\rho_{p}}\left( u_{i}-u_{p,i} \right)\left\| u_{i}-u_{p,i} \right\|+g_{i}\#35.2 \end{aligned}$$

$u_{p,i}$ is the velocity and $C_{d}$the drag coefficient given by **Equation 36**.

$$\begin{aligned} C_{d}=\left\{ \begin{matrix} \frac{24}{Re} & \text{if}\text{ }Re\leq1 \\ \frac{24}{Re}\left( 1+0.15\cdot Re^{0.687} \right) & \text{if}\text{ }1<Re\leq1000 \\ 0.44 & \text{if}\text{ }Re>1000 \end{matrix} \right.\#36.1 \end{aligned}$$

$$\begin{aligned} Re=\frac{d_{p}\left\| u_{i}-u_{p,i} \right\|}{\nu}\#36.2 \end{aligned}$$

The near-field exposure was calculated from the droplets directly reaching the receiver. The near-field to far-field ratio is given in **Figure S6** as a function of the lateral distance of the receiver relative to the trajectory of the emitted plume.

| 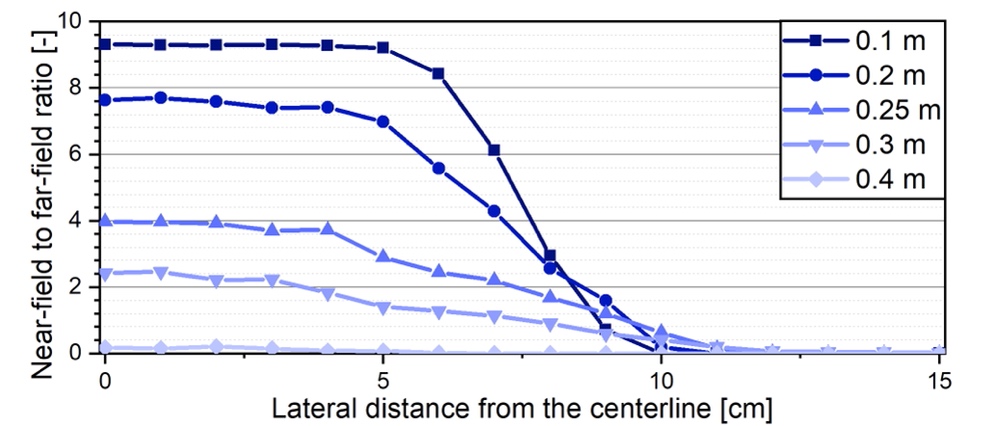 |
| --- |
| **Figure S6**: Near-field exposure level as a function of the lateral position of the receiver relative to the emitter |

A9. Accumulation of viral charge in a closed volume

The calculation of the far-field viral charge was based on the accumulation of contaminated droplets over a longer time scale. The method is used by [25] for a similar calculation and is based on [26]. The evolution of the viral charge over time $\frac{dc}{dt}$ is given by **Equation 37**, as a function of the ventilation rate $Q$, the room’s volume $V_{m}$, the settling velocities on horizontal and vertical surfaces, the decay rate $\lambda$ and the viral shedding $\sigma$.

$$\begin{aligned} V_{m}\frac{dc}{dt}=-\left( Q+v_{dv}\cdot S_{v}+v_{du}\cdot S_{u}+v_{dd}\cdot S_{d}+\lambda\cdot V_{m} \right)\cdot c+\sigma\#37 \end{aligned}$$

The deposition velocities are calculated in **Equation 38** on vertical (38.1), upward-facing horizontal (38.2) and downward-facing horizontal surfaces (38.3).

$$\begin{aligned} v_{dv}=\frac{u^{*}}{I}\#38.1 \end{aligned}$$

$$\begin{aligned} v_{du}=\frac{v_{s}}{1-e^{-\frac{v_{s}I}{u^{*}}}}\#38.2 \end{aligned}$$

$$\begin{aligned} v_{dd}=\frac{v_{s}}{e^{\frac{v_{s}I}{u^{*}}}-1}\#38.3 \end{aligned}$$

$$\begin{aligned} I=3.64\cdot Sc^{2/3}\cdot\left( a-b \right)+39\#38.4 \end{aligned}$$

$$\begin{aligned} a=\frac{1}{2}ln\frac{\left( 10.92\cdot Sc^{-\frac{1}{3}}+4.3 \right)^{3}}{Sc^{-1}+0.0609}+\sqrt{3}\cdot arctan\frac{8.6-10.92\cdot Sc^{-\frac{1}{3}}}{\sqrt{3}\cdot10.92\cdot Sc^{-\frac{1}{3}}}\#38.5 \end{aligned}$$

$$\begin{aligned} b=\frac{1}{2}ln\frac{\left( 10.92\cdot Sc^{-\frac{1}{3}}+r^{+} \right)^{3}}{Sc^{-1}+7.669\cdot{10}^{-4}\cdot\left( r^{+} \right)^{3}}+\sqrt{3}arctan\frac{2r^{+}-10.92\cdot Sc^{-\frac{1}{3}}}{\sqrt{3}\cdot10.92\cdot Sc^{-\frac{1}{3}}}\#38.6 \end{aligned}$$

$$\begin{aligned} r^{+}=\frac{d_{p}\cdot u^{*}}{2\nu}\#38.7 \end{aligned}$$

$$\begin{aligned} u^{*}=\sqrt{\frac{\tau_{w}}{\rho_{a}}}=\sqrt{\frac{C_{f}}{2}}U_{avg}\#38.8 \end{aligned}$$

$$\begin{aligned} \tau_{w}=C_{f}\frac{1}{2}\rho_{a}U_{avg}^{2}\#38.9 \end{aligned}$$

$$\begin{aligned} C_{f}=\frac{0.027}{Re^{\frac{1}{7}}}\#38.10 \end{aligned}$$

$$\begin{aligned} Re=U_{avg}\frac{d_{h}}{\nu}\#38.11 \end{aligned}$$

The Schmidt number is given by **Equation 39.1**, D is the diffusion coefficient and $\nu$ the kinematic viscosity of air. The settling velocity in **Equation 39.2** ([4]).

$$\begin{aligned} Sc=\frac{\nu}{D}\#39.1 \end{aligned}$$

$$\begin{aligned} v_{s}=\frac{\rho_{p}d_{p}^{2}gC_{c}}{18\eta}\#39.2 \end{aligned}$$

$C_{c}$ is the slip correction factor.

The decay rate$\lambda$ is calculated from the half-life of the virus $t_{\frac{1}{2}}$=1.1 hour [32], and given in **Equation 40**.

$$\begin{aligned} \lambda=\frac{\ln2}{t_{\frac{1}{2}}}\#40 \end{aligned}$$

The evolution of the viral charge accumulated during one hour for different expiratory activities is given in **Figure S7**. Speaking was combined with breathing, both activities were considered for the same duration. Cough and sneeze happen once at the beginning of the interaction, followed by constant breathing.

| 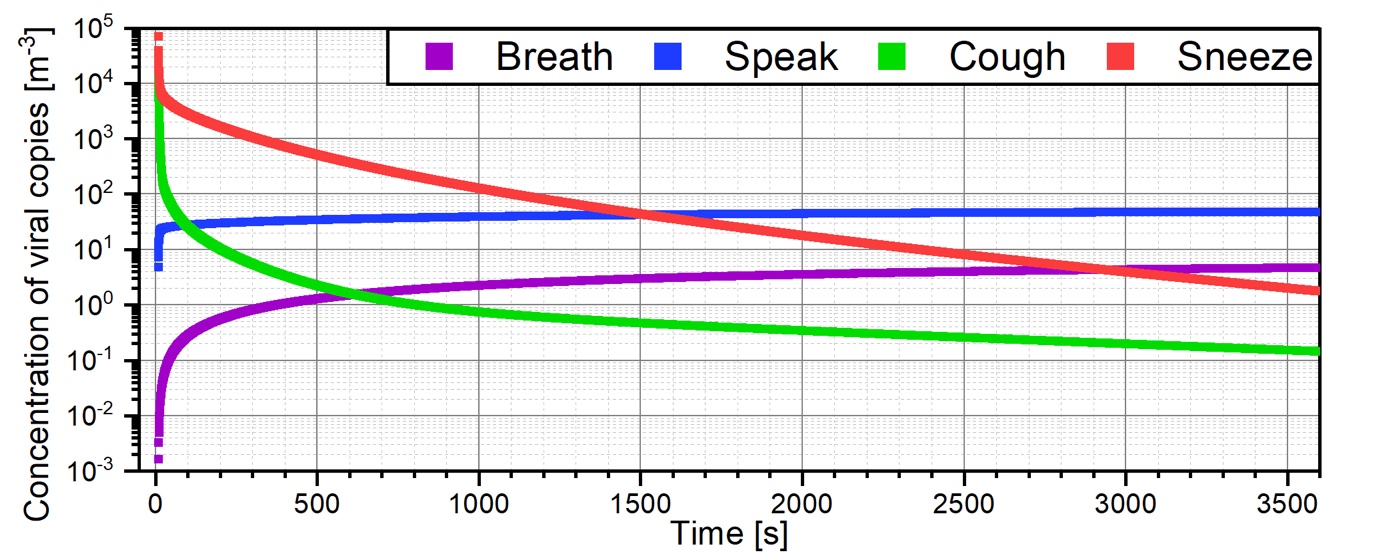 |
| --- |
| **Figure S7**: Evolution of the viral charge accumulated in the surroundings of the emitter, considering one hour of breathing, one hour of speaking, a single sneeze, and a single cough (not combined with breathing).  A10. Calculation of the inhaled viral charge  **A10.1 Near-field exposure level** |

The calculation of the near-field exposure level was based on the reach-rate for each particle size, describing the number-based fraction of the emitted particles reaching the receiver’s face. The reach-rate was multiplied by the emitted particle count and the viral charge per droplet, the lung deposition fraction and the fraction penetrating the receiver’s facemask. The emitted count corresponded to one minute of breathing and speaking, and is scaled to the desired interaction time (60 minutes for the data presented in this work). The data for a cough and a sneeze correspond to a single event and were not scaled down.

**A10.2 Far-field exposure level**

The far-field exposure level was calculated from the concentration of viral copies in the air surrounding the emitter. As shown in **Figure S7**, it depended on the time. The viral shedding for breathing and speaking corresponded to one minute and was scaled down to one second (and adapted to the time step used for the calculation). The inhalation followed a breathing pattern (taken at 0.25 Hz breathing frequency and 2s inhalation in this work) and the concentration (viral_copies/m^3^) was summed at each time step corresponding to an inhalation and matched with the volume inhaled during the time step (depending on the total inhalation time and the considered tidal volume).

A11. Lung deposition model

The fraction of droplets depositing in the respiratory tract was modelled using the NCRP (National Council on Radiation Protection and Measurements) model, based on both empirical and theoretical equations. The equations and parameters were taken from [27] and [4]. The lung morphology used in the model is given in Table S10, taken from data published in [27] and [47].

| n | N | D [cm] | L [cm] | θ [°] | ϕ [°] | S [cm2] | V [cm3] | Vel. [cm/s] | Re [-] |
| --- | --- | --- | --- | --- | --- | --- | --- | --- | --- |
| 1 | 1 | 2.01 | 10 | 1 | 1 | 3.17 | 31.73 | 197 | 2084 |
| 2 | 2 | 1.56 | 4.36 | 33 | 20 | 3.82 | 16.67 | 215 | 1540 |
| 3 | 4 | 1.13 | 1.78 | 34 | 31 | 4.01 | 7.14 | 236 | 1151 |
| 4 | 8 | 0.827 | 0.965 | 22 | 43 | 4.3 | 4.15 | 251 | 827 |
| 5 | 16 | 0.651 | 0.995 | 20 | 39 | 5.33 | 5.3 | 202 | 533 |
| 6 | 32 | 0.574 | 1.01 | 18 | 39 | 8.28 | 8.36 | 161 | 331 |
| 7 | 64 | 0.435 | 0.89 | 19 | 40 | 9.51 | 8.47 | 126 | 208 |
| 8 | 128 | 0.373 | 0.962 | 22 | 36 | 13.99 | 13.46 | 98 | 133 |
| 9 | 256 | 0.322 | 0.867 | 28 | 39 | 20.85 | 18.07 | 72 | 79 |
| 10 | 512 | 0.257 | 0.667 | 22 | 45 | 26.56 | 17.72 | 52 | 47 |
| 11 | 1024 | 0.198 | 0.556 | 33 | 43 | 31.53 | 17.53 | 37 | 28 |
| 12 | 2048 | 0.156 | 0.446 | 34 | 45 | 39.14 | 17.46 | 26 | 16 |
| 13 | 4096 | 0.118 | 0.359 | 37 | 45 | 44.79 | 16.08 | 17 | 9.7 |
| 14 | 8192 | 0.092 | 0.275 | 39 | 60 | 54.46 | 14.98 | 11 | 5.4 |
| 15 | 16384 | 0.073 | 0.212 | 39 | 60 | 68.57 | 14.54 | 7.2 | 3.1 |
| 16 | 32768 | 0.06 | 0.168 | 51 | 60 | 92.65 | 15.57 | 4.3 | 1.3 |
| 17 | 65536 | 0.054 | 0.134 | 45 | 60 | 150.09 | 20.11 | 2.2 | 0.64 |
| 18 | 131072 | 0.05 | 0.12 | 45 | 60 | 257.36 | 30.88 | 1.7 | 0.39 |
| 19 | 262144 | 0.047 | 0.092 | 45 | 60 | 454.81 | 41.84 | 0.92 | 0.21 |
| 20 | 524288 | 0.045 | 0.08 | 45 | 60 | 833.34 | 66.71 | 0.51 | 0.11 |
| 21 | 1048576 | 0.044 | 0.07 | 45 | 60 | 1594.39 | 111.61 | 0.29 | 0.057 |
| 22 | 2097152 | 0.044 | 0.063 | 45 | 60 | 3188.78 | 200.89 | 0.18 | 0.033 |
| 23 | 4194304 | 0.043 | 0.057 | 45 | 60 | 6090.97 | 347.19 | 0.099 | 0.017 |
| 24 | 8388608 | 0.043 | 0.053 | 45 | 60 | 12181.95 | 645.64 | 0.066 | 0.01 |
| 25 | 16777216 | 0.03 | 0.025 | 45 | 60 | - | 3871.8 | - | - |

**Table S10**: Lung morphology used in the lung deposition module of the proposed model. The data are taken from [27] and [47]. n = generation number; N = number of airways per generation; D = airway diameter; L = airway length; θ = branching angle; ϕ = inclination angle relative to gravity; S = cross section; V = volume; Vel. = average airflow velocity; Re = average airflow Reynolds number.

The inhalability represents the fraction of droplets that reach the nose and mouth and can therefore enter the respiratory system. The nose inhalability is given in **Equation 41**, the mouth equation is given in **Equation 42**.

$$\begin{aligned} I_{n}\left( d_{p} \right)=0.035+0.965e^{-0.000113d_{p}^{2.74}}\#41 \end{aligned}$$

$$\begin{aligned} I_{0}\left( d_{p} \right)=0.5\left[ 1+e^{-0.06d_{p}} \right]\text{ }\text{for}\text{ }1<U_{0}<4\text{m/s}\text{ }\text{and}\text{ }d_{p}<100\mu m\#42.1 \end{aligned}$$

$$\begin{aligned} I_{0}\left( d_{p},U_{0} \right)=0.5\left[ 1+e^{-0.06d_{p}} \right]+{10}^{-5}U_{0}^{2.75}e^{0.055d_{p}}\text{ }\text{for}\text{ }4<U_{0}<9 \text{m/s}\text{ }\text{and}\text{ }d_{p}<100 \mu m\# 42.2 \end{aligned}$$

$$\begin{aligned} I_{0}\left( d_{p} \right)=24.14+75.86\cdot e^{-0.00607d_{p}^{1.4}}\text{ }\text{for}\text{ }0.4<U_{0}<1.6\text{m/s}\text{ }\text{and}\text{ }d_{p}<141\mu m\#42.3 \end{aligned}$$

$$\begin{aligned} I_{0}\left( d_{p} \right)=1-0.0038d_{p}\text{for}\text{ }U_{0}<1\text{m/s}\#42.4 \end{aligned}$$

Once the inhalable particles enter the respiratory system, they travel through the extra thoracic regions (upper airways) where a fraction is deposited. This deposition is different for nose or mouth inhalation and for inhalation and exhalation and is described by **Equations 43** and **44**.

$$\begin{aligned} \eta_{In}=\frac{1}{1+(0.000217\rho d_{p}^{2}Q)^{-0.94}}\text{ }\text{nose}\text{ }\text{inhalation}\text{, }\text{for}\text{ }d_{a}>0.2 \mu m\#43.1 \end{aligned}$$

$$\begin{aligned} \eta_{En}=\frac{1}{1+(0.000435\rho d_{p}^{2}Q)^{-1.01}}\text{ }\text{nose}\text{ }\text{exhalation}\text{, }\text{for}\text{ }d_{a}>0.2 \mu m\#43.2 \end{aligned}$$

$$\begin{aligned} \eta_{Io}=\frac{1}{1+(0.0000333\rho d_{p}^{2}Q)^{-1.37}}\text{ }\text{mouth}\text{ }\text{inhalation}\text{, }\text{for}\text{ }d_{a}>0.2 \mu m\#43.3 \end{aligned}$$

$$\begin{aligned} \eta_{Eo}=\eta_{Io} mouth exhalation, \text{f}\text{or}\text{ }d_{a}>0.2\mu m\#43.4 \end{aligned}$$

$$\begin{aligned} \eta_{In}=1-e^{-18.2D^{\frac{1}{2}}Q^{-\frac{1}{8}}}\text{ }\text{nose}\text{ }\text{inhalation}\text{, }\text{for}\text{ }d_{a}<0.2\mu m\#44.1 \end{aligned}$$

$$\begin{aligned} \eta_{En}=1-e^{-21.3D^{\frac{1}{2}}Q^{-\frac{1}{8}}}\text{ }\text{nose}\text{ }\text{exhalation}\text{, }\text{for}\text{ }d_{a}<0.2\mu m\#44.2 \end{aligned}$$

$$\begin{aligned} \eta_{Io}=1-e^{-14.6D^{\frac{1}{2}}Q^{-\frac{1}{8}}}\text{ }\text{nose}\text{ }\text{inhalation}\text{, }\text{for}\text{ }d_{a}<0.2\mu m\#44.3 \end{aligned}$$

$$\begin{aligned} \eta_{Eo}=1-e^{-12.1D^{\frac{1}{2}}Q^{-\frac{1}{8}}}\text{ }\text{nose}\text{ }\text{inhalation}\text{, }\text{for}\text{ }d_{a}<0.2\mu m\#44.4 \end{aligned}$$

The deposition in the thoracic regions (trachea, lungs, alveola) is dominated by Brownian diffusion, gravitational settling and inertial impaction. The lungs geometry as well as the airflow data in each compartment are taken from [27]. The flow velocities and lung dimensions were adapted to the initial parameters considered in the model.

The deposition by diffusion is given in **Equations 45.1** (turbulent flow) and **45.2** (laminar flow). The deposition during a pause (no flow) is given in **Equation 45.3** and the deposition in the vicinity of a carinal ridge is given by **Equation 45.4**.

$$\begin{aligned} \eta_{td}=3.544\left( \frac{Dl_{t}}{Q} \right)^{\frac{1}{2}}-1.395\frac{Dl_{t}}{Q}\#45.1 \end{aligned}$$

$$\begin{aligned} \eta_{tl}=0.819e^{-11.49\frac{Dl_{t}}{Q}}+0.0976e^{-70.07\frac{Dl_{t}}{Q}}+0.0325e^{-179\frac{Dl_{t}}{Q}}+0.0509e^{-107.2\left( \frac{Dl_{t}}{Q} \right)^{\frac{2}{3}}}\#45.2 \end{aligned}$$

$$\begin{aligned} \eta_{d}=1-e^{-7.712\frac{kTC_{c}t_{h}}{\pi\mu d_{p}d_{t}^{2}}}\#45.3 \end{aligned}$$

$$\begin{aligned} \eta_{e}=1-\left( 1-\eta_{ld} \right)^{1+\frac{2\theta}{\pi}\left( 13-\frac{12\theta}{\pi} \right)\frac{d_{t}}{l_{t}}}\#45.4 \end{aligned}$$

$\eta_{ld}$ is the diffusion in a tube under laminar flow. The deposition by gravitational settling is given in **Equation 46**. If the flow velocity is zero (during a breathing pause), the term $\frac{l_{t}}{U}$ is replaced by the duration of the breathing pause.

$$\begin{aligned} \eta_{s}=1-e^{-\frac{2gC_{c}\rho d_{p}^{2}l_{t}\sin\phi}{9\pi\mu d_{t}U}}\#46 \end{aligned}$$

The deposition by inertial impaction is given by **Equations 47.1** and **47.2**.

$$\begin{aligned} \eta_{i}=1-\frac{2}{\pi}arccos\left( \theta\cdot Stk \right)+\frac{1}{\pi}sin\left( 2arccos\left( \theta\cdot Stk \right) \right), \text{for}\text{ }\theta\cdot Stk<1\#47.1 \end{aligned}$$

$$\begin{aligned} \eta_{i}=1 ,\text{for}\text{ }\theta\cdot Stk\geq1\#47.2 \end{aligned}$$

$$\begin{aligned} Stk=\frac{\rho d_{p}^{2}UC_{c}}{18\mu d_{t}}\#47.3 \end{aligned}$$

The calculation of the overall deposition in the thoracic regions is based on a compartments-in-series model, considering the deposition in each individual lung compartment for both inhalation and exhalation. The fractional deposition in the different compartments is given by **Equation 48** during inhalation and by **Equation 49** during exhalation.

$$\begin{aligned} E_{Ii}=\prod_{n=1}^{i-1} \left( 1-\eta_{In} \right)\left[ \eta_{Ii}\cdot\left( \sum_{n=i+1}^{f} V_{n} \right)+\alpha_{Ii}\eta_{Ii}V_{i} \right]\frac{1}{V_{t}}\#48 \end{aligned}$$

﻿$E_{Ii}$ is the fractional deposition in compartment i during inhalation, $\eta_{Ii}$represents the efficiency of deposition in compartment i during inhalation, $V_{i}$is the volume of compartment i, $V_{f}$ is the volume of the final compartment, $V_{t}$is the total volume of the lungs and $\alpha_{Ii}$is a weighing factor and depends on the residence time of the particles in compartment i. It is taken as 0.5.

$$\begin{aligned} E_{Ei}=\left[ \prod_{j=1}^{i-1} \left( 1-\eta_{Ij} \right) \right]\cdot\left( 1-\alpha_{Ii}\eta_{Ii} \right)\alpha_{Ei}\eta_{Ei}\frac{V_{i}}{V_{t}}+\left[ \prod_{j=1}^{i} \left( 1-\eta_{Ij} \right) \right]\cdot\eta_{Ei}\cdot\\ \sum_{j=i+1}^{f} \left[ \prod_{k=i+1}^{j-1} \left( 1-\eta_{Ek} \right)\left( 1-\eta_{Ik} \right) \right]\cdot\left( 1-\alpha_{Ij}\eta_{Ik} \right)\left( 1-\alpha_{Ej}\eta_{Ej} \right)\frac{V_{j}}{V_{t}}\#49 \end{aligned}$$

The total deposition in the compartment i during a complete breathing cycle is given by **Equation 50**.

$$\begin{aligned} E_{i}=E_{Ii}+E_{Ei}\#50 \end{aligned}$$

The deposition in the thoracic regions $E$ is the sum of the fractional deposition coefficients. The total deposition in the respiratory system is given by **Equations 51.1** to **51.4**. 51.1 is the fraction deposited in the upper airways at inhalation, 51.2 the deposition in the lungs, 51.3 represents the deposition in the upper airways at exhalation and 51.4 is the total deposition in the lungs.

$$\begin{aligned} \eta_{up,In}=I_{0}\cdot\eta_{In}\#51.1 \end{aligned}$$

$$\begin{aligned} \eta_{lungs}=I_{0}\cdot\left( 1-\eta_{In} \right)\cdot E\#51.2 \end{aligned}$$

$$\begin{aligned} \eta_{up, Ex}=I_{0}\left( 1-\eta_{In} \right)\left( 1-E \right)\eta_{Ex}\#51.3 \end{aligned}$$

$$\begin{aligned} \eta_{total}=\eta_{up,In}+\eta_{lungs}+\eta_{up,Ex}\#51.4 \end{aligned}$$

The NCRP model describes the deposition of particles up to 100 nm. The model developed in this work considered sizes up to 1 mm, the deposition of the particles between 100 nm and 1 mm had therefore to be approximated. Droplets larger than 100 nm followed a ballistic trajectory and were only marginally influenced by the surrounding airflow. The assumption was made that during mouth inhalation, particles with a diameter larger than 100 nm have the same probability to deposit in the mouth than 100 nm droplets, and the inhalability was considered constant. For nose inhalability however, the deposition rate is strongly dependent on the droplet’s relaxation time as it has to follow the airflow to enter the nose. The inhalability was therefore considered to be proportional to the relaxation time. The fraction of inhaled droplets depositing in the respiratory system as well as the considered nose and mouth inhalability are given in **Figure S8**.

| 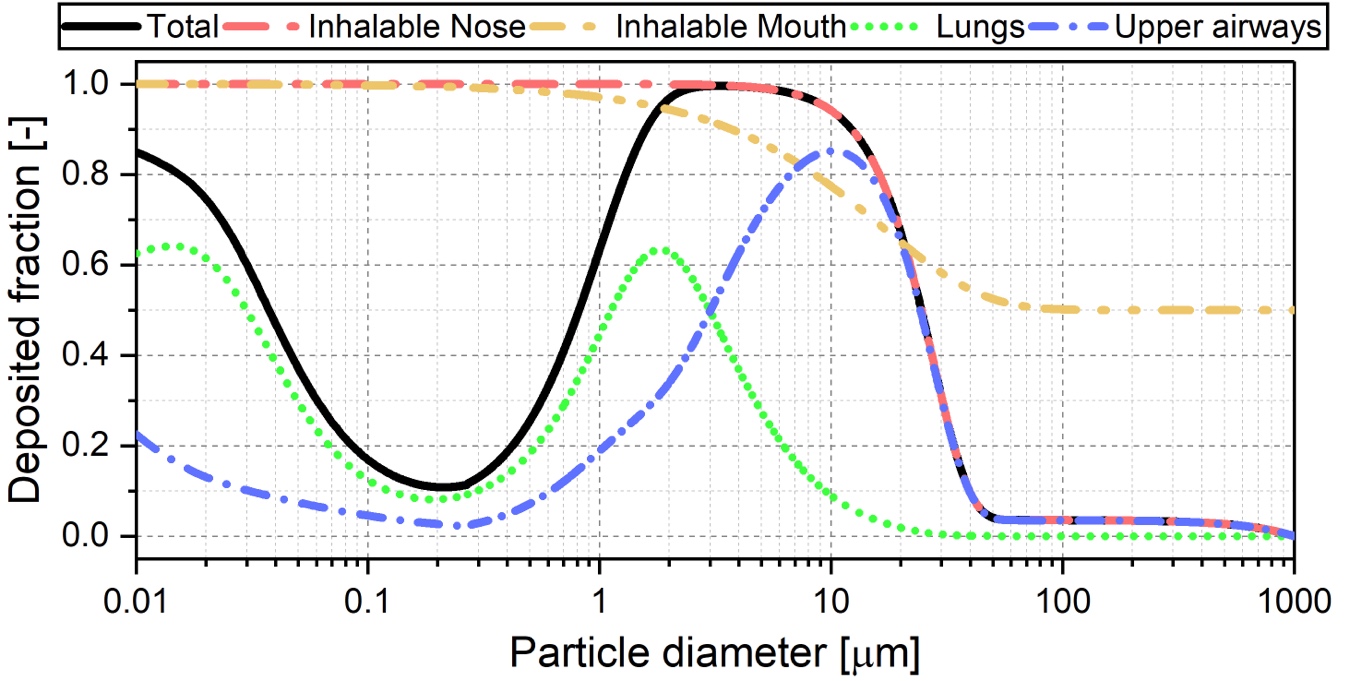 |
| --- |
| **Figure S8**: Lung deposition as a function of the particle size, based on the NCRP deposition model. The partial depositions in the lungs and in the upper airways are also included, as well as the inhalable fraction considering nose breathing and mouth breathing. |

A12. Calculation of the viral charge

The viral charge was calculated based on the droplets deposited in the respiratory system and on the viral charge carried by the droplets. The calculation of the viral charge is given in **Equation 52**.

$$\begin{aligned} C_{v}=L_{v}\cdot\frac{4}{3}\pi\left( \frac{d_{init}}{2} \right)^{3}\#52 \end{aligned}$$

﻿$L_{v}$ is the concentration of plaque forming units in the pulmonary fluid. The initial size at emission $d_{init}$ was considered for the calculation of the initial liquid volume and therefore the concentration of viral copies. The evaporation of particles only changed their mechanical properties without changing their viral load.

1. Implementation

The complete model is composed of different modules as shown in Figure 1 of the main text. Their implementation is detailed in this section.

B1. Interaction between the emitted airflow and the emitter’s mask

An overview of the inputs and outputs of the first module is given in **Figure S9**.

| **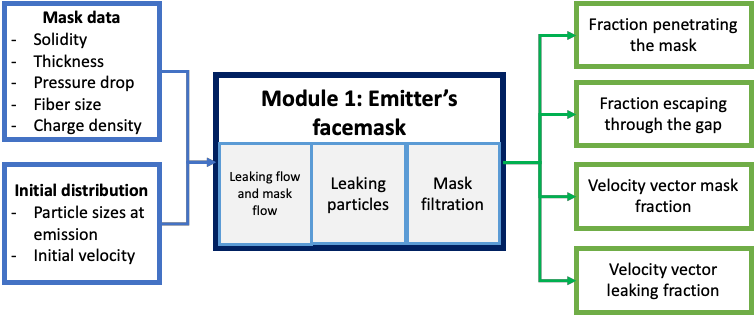** |
| --- |
| **Figure S9:** Summary of the module describing the influence of the emitter’s facemask |

B1.1 Generation of the input data

The parameters of the emitter’s facemask were loaded from a text file containing the parameters to calculate the filtration efficiency: solidity, thickness, average fiber size, charge density, and fiber size distribution. The file also contains the viscous porous resistance at inhalation and exhalation, calculated from the measured or required pressure drop. These values were used in the calculation of the flow resistance to determine the leaking flow. The emitted size distribution was imported from another text file, containing the configuration of the emission scenario (particle size at emission, particle count, and initial velocity). Both modules are described in section **B7** and **B8**. The diameters at emission were used for the calculation of the penetration through the emitter’s mask. It was considered that no evaporation occurred at this stage.

The mask’s fiber size matrix was created from a normal distribution taking the average fiber size and its standard deviation as inputs.

B1.2 Calculation of the flow velocities

The initial volumetric flow was calculated from the opening diameter (mouth/nose) and the emission velocity gathered from the available literature. The volumetric flow going through the mask was defined as the difference between the total emitted flow and the volumetric flow through the leakage.

The leakage was defined by three different methods:

- Set a fixed leaking fraction: the leaking fraction was set between 0 and 1 and defined the fraction of the emitted volumetric flow going into the leaking flow, the remaining volumetric flow went through the mask and the corresponding velocities were derived.
- Set a gap dimension: the length, width, and height of the gap between the wearer’s face and the facemask were manually set and the leaking fraction was calculated accordingly
- Set the pressure drop generated by the mask: the gap’s dimension was adjusted in order to reach the desired pressure drop through the facemask.

B1.3 Calculation of the penetrating fraction

The velocities calculated in the previous sections were attributed to the emitted flow, impactor flow, leaking flow, and mask flow as shown in **Figure S10**.

| 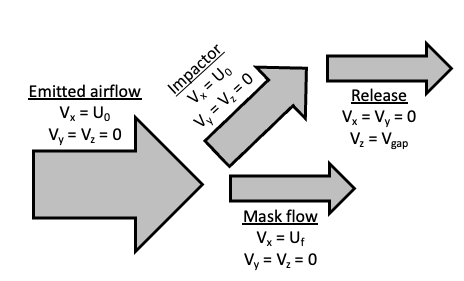 |
| --- |
| **Figure S10**: Velocities attributed to the mask and leaking flows. The velocity of the flow going through the impactor was considered to be equal to the emitted flow U_0_. The released flow was accelerated to V_gap_ when going through the leakage and considered to be redirected upward, modelling a leakage around the nose. The remaining flow was going through the mask with the calculated face velocity U_f_. In this module, the particles were considered to have the same velocity as the airflow they travel in. The influence of the relaxation time was taken into account in the next module. |

The distribution of the particles between the leaking flow and the mask flow was done considering an impactor to take into account the change of direction of the leaking flow. The principle is summarized in **Figure S11**. The calculation of the impactor’s cut-off size was based on the mouth diameter and the velocity of the emitted airflow (which was different for breathing, speaking, coughing, and sneezing). Both the mask flow and the leaking flows were virtually separated: the mask flow penetrated the mask and the carried droplets were filtered. The leaking flow did not penetrate the mask, which acted as the plate of the impactor and fully diverted the flow. The ratio between the mask flow and the leaking flow was determined by the equilibrium of the flow resistances generated by the mask and the gap. Particles that fail to follow the diverted leaking flow (larger than the cut-off size) were counted in the mask flow where they interacted with the mask.

| 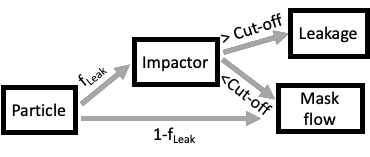 |
| --- |
| **Figure S11**: Distribution of the emitted particles between the leaking flow and the mask flow. |

B2. Trajectories and evaporation

The second module calculated the trajectories of the particles. In each simulation, this module ran twice to consider both the absence of the emitter’s mask (particles emitted from the mouth) and the presence of the mask (particles emitted from the mask and leakage).

| 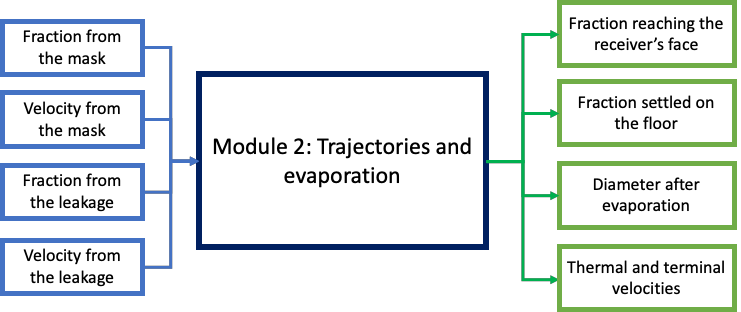 |
| --- |
| **Figure S12**: Summary of the module describing the influence of the emitter’s facemask |

B2.1 Generation of particles

The first iteration of the calculation was done without the emitter’s mask. The start area was the mouth/nose and the initial velocity was the exhalation velocity U_0_. Emission points were defined in the starting area, regularly spaced along the X and Y axis. A standard calculation considered about 300 emission points, and for each size one particle was generated at each point.

The emitter’s mask was worn for the second iteration. The generated particles were attributed to the leaking flow or the mask flow with a probability equal to the leaking or mask fraction.

| 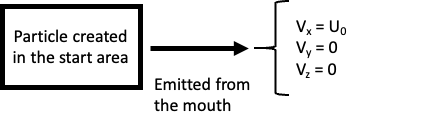 |
| --- |
| 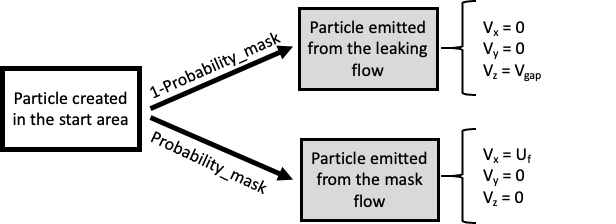 |
| **Figure S13**: Emission of particles whether the calculation considered that the emitter did not wear a mask (a) or did wear a mask (b). |

B2.2 Calculation of the particles’ trajectories

The calculation of the trajectories, also including the drying, was performed at discrete time intervals. The differential equations of motion were numerically solved using Euler’s method (**Equation 53**) to calculate the acceleration, velocity, and position at each time step.

$$\begin{aligned} f\left( t \right)=f\left( t-1 \right)+\frac{df\left( t \right)}{dt}\cdot\Delta t\#53 \end{aligned}$$

The position of the particle was calculated at every time step until the maximum time was reached. This time was set to 10 s in the present model, this value is discussed in the sensitivity assessment (**Section C**). The variables calculated during one time step are presented in **Figure S14**. The influence of the evaporation rate on the particle’s diameter was considered in the particle’s motion. The computation was repeated for every generated particle and each size. One iteration of the module lead to the calculation of 300 particles for each size, 36 size classes were considered, leading to the trajectories of 10’800 particles.

| 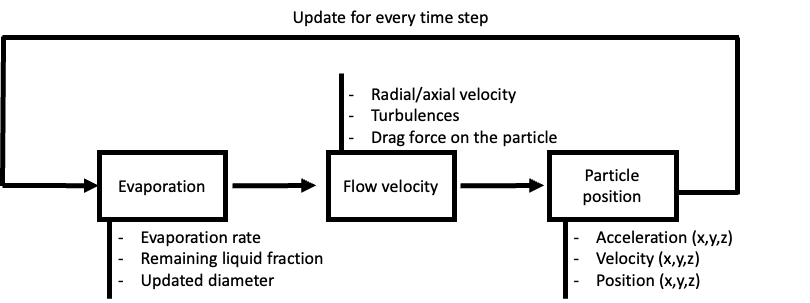 |
| --- |
| **Figure S14**: Variables updated at every time step |

B2.3 Output of the module

The module calculated for each droplet, whether it reached the receiver’s face or not. The receiver’s face was modelled by disk with a diameter of 15 cm placed at a given distance along the x-axis from the emission point. The fraction reaching the receiver’s face was included in the calculation of the near-field exposure level, as shown in **Figure S15**.

| 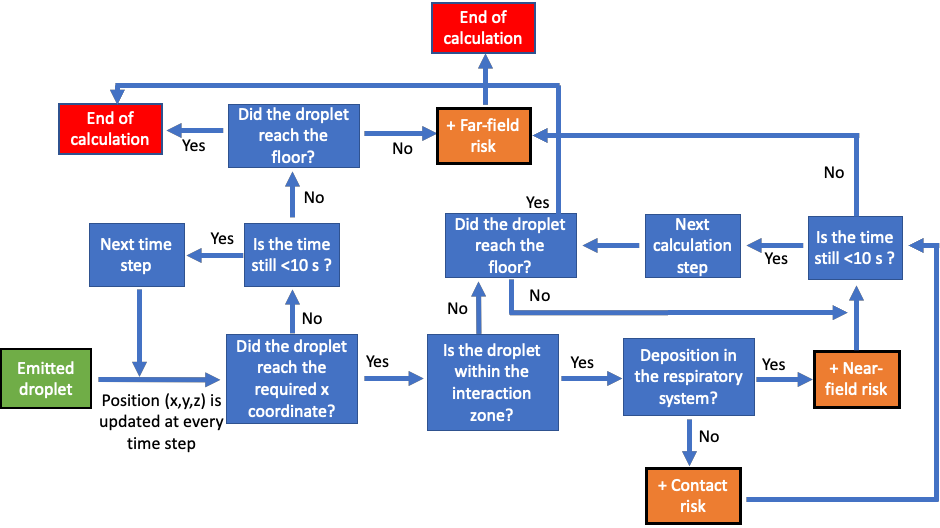 |
| --- |
| **Figure S15**: Calculation of the near-field, far-field, and contact exposure levels |

Other parameters calculated in this module were needed for further calculation:

- The fraction of particles settled at the end of the 10 s calculation
- The final diameter after evaporation and the droplet nuclei size. Larger droplets did not fully evaporate, but they were removed from the calculation as they settle on the floor. More information is given in the sensitivity analysis (**Section C**).
- The droplet nuclei size used for the calculation of the viral charge for each droplet size
- The thermal and terminal velocities used in the calculation of the accumulation of viral charges

The total viral charge calculated from the particles reaching the receiver’s face was calculated based on the droplet nuclei size and the initial diameter (before evaporation). A summary of the different considered particles sizes is given in **Figure S16**.

| 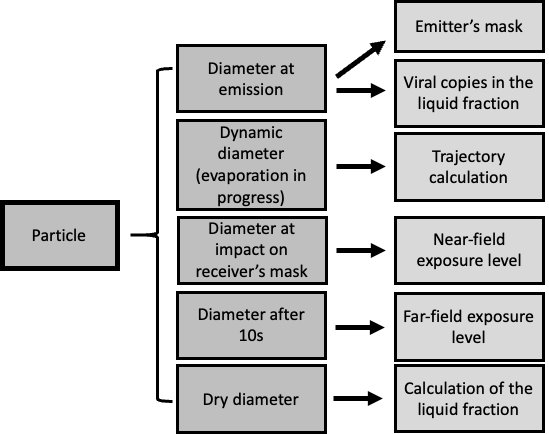 |
| --- |
| **Figure S16**: Consideration of the different particle sizes for the calculation of the exposure level |

B3. Accumulation of viral charges around the emitter

Viral charges carried by small particles with a high residence time (several minutes or hours) accumulate around the emitter, and can spread over long distances due to air movements (wind, ventilation, convection) or diffusion. The mechanical properties of the particles (settling velocity, thermal velocity) were calculated based on their diameter after evaporation. This assessment is not valid for larger droplets (>100 μm) as they do not fully evaporate within the 10 s considered in the previous module. However, they did not influence the calculation of the far-field viral charge due to their high settling velocity.

| 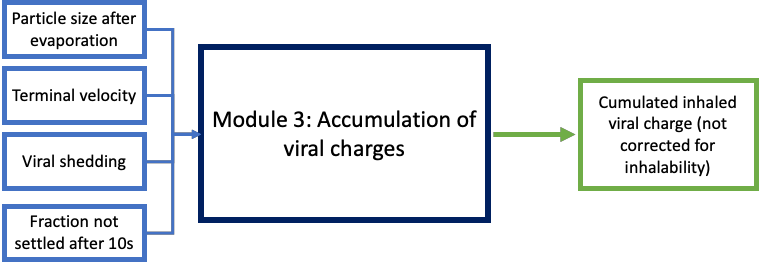 |
| --- |
| **Figure S17**: Structure of the module calculating the accumulation of viral charges around the emitter |

The viral shedding was calculated considering the number of viral copies in the liquid fraction of one droplet in each size bin and multiplied by the number of particles of each size emitted during one second (or one cough/sneeze). For recurring activities (speaking and breathing), the shedding was considered as constant for the entire duration of the interaction. For coughing and sneezing, the viral charges were emitted during the first step only.

The viral charges were removed through decay, ventilation, and deposition on the room’s surfaces.

The total interaction time was set to one hour and the time resolution to 1 s. The total viral charge inhaled during the interaction was calculated based on the lung’s tidal volume, the breathing frequency, and the duration of an inhalation. A frequency of 15 movements per minute was considered, with an inhalation duration of 2s. The average inhaled volume was the tidal volume, estimated at 0.77 L. The principle is illustrated in **Figure S18**. The concentration at the beginning of the inhalation was multiplied by the lung’s volume for each inhalation, and the total inhaled viral charge was the sum of all the single inhalations.

| 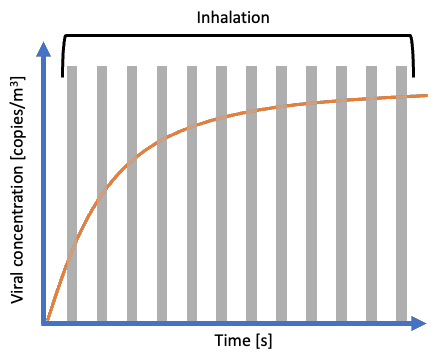 |
| --- |
| **Figure S18**: Illustration of the calculation of the viral charge carried by the inhaled airflow |

B4. Filtration performances of the receiver’s facemask

The receiver’s facemask was modelled with a similar method than the emitter’s facemask. The major differences lie on the flow velocity and on the considered particle diameter.

The flow velocity was constant and did not depend on the expiratory activity, which only impacted the emitter’s exhaled flow. The total inhaled flow was based on the assumption that the receiver inhaled at 30 L/min through a 2 cm opening (mouth or nose), leading to a flow velocity of 1.59 m/s. The flow balance between the leaking flow and the mask flow was calculated in the same way as for the emitter’s mask. The virtual impactor was based on the face velocity and the mask diameter to calculate the fraction not following the leaking flow.

The virtual impactor and the facemask both consider the diameter after evaporation. A summary of them module is given in **Figure S19**.

| 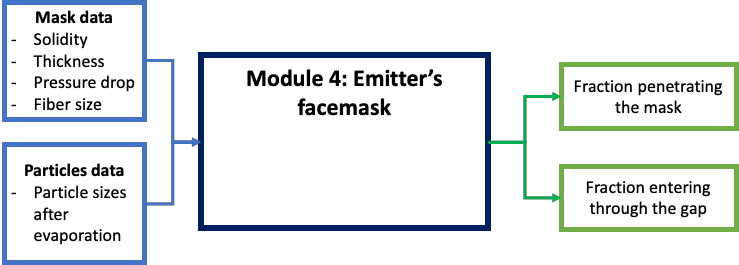 |
| --- |
| **Figure S19**: Inputs and outputs of the module describing the filtration efficiency of the emitter’s facemask |

B5. Lung deposition model

The lung deposition was based on the NCRP model (National Council on Radiation Protection and Measurements), which has been chosen over the ICRP model (International Commission on Radiological Protection) because the analytical equations make it easier to adapt the calculation to different lung parameters.

The calculation of the deposition in the lungs was based on the inhalation velocity calculated in the previous module (1.59 m/s, corresponding to 30 L/min) and considered the diameter of the particles after evaporation.

| 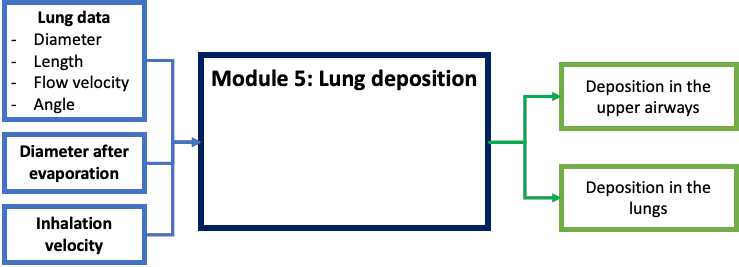 |
| --- |
| **Figure S20**: Structure of the module calculating the fraction deposited in the lungs based on the NCRP deposition model |

1. Sensitivity assessment

The dependence of the model’s output on several parameters having a large uncertainty was assessed. The considered parameters and their limits are given in **Table S11**.

| **Parameter** | **Lower limit** | **Upper limit** | **Value considered** |
| --- | --- | --- | --- |
| Dose-response relationship | 10^3^ | 10^7^ | 1.5*10^5^ |
| Particle initial count | Low | Top | Top |
| Viral concentration | 10^10^ | 10^16^ | 10^13^ |
| Viral decay | 1 min | 10 hr | 1.1 hr |
| Room size | 10 m^2^ | 1000 m^2^ | 100 m^2^ |
| Temperature | 0°C | 40°C | 20°C |
| Humidity | 0% | 100% | 50% |
| **Table S11**: Parameters considered in the sensitivity analysis | | | |

The **dose-response relationship** is the link between the inhaled viral charge and the infection risk. It is calculated according to **Equation 53**. $p$ is the infection risk, $C_{v}$ the viral charge and $k$ the dose-response relationship.

$$\begin{aligned} p=1-e^{- \frac{C_{v}}{k}}\#53 \end{aligned}$$

The influence of a variation in the dose-response coefficient $k$ is given in **Figure S21a**, while the near-field, far-field, and contact exposure levels as a function of the coefficient $k$ is given in **Figure S21b**. The relation between the infection risk and the viral charge can be approximated by a linear function for an infection risk between 0.2 and 0.8. However, the viral charge leading to an infection risk within that range is highly dependent on the dose-response coefficient, and moving away from the linear range (infection risk approaching 0 or 1) makes the comparison of the facemasks also dependent from the dose-response coefficient. Therefore, the comparison between the facemasks was based on the viral charge and not on the infection risk.

| 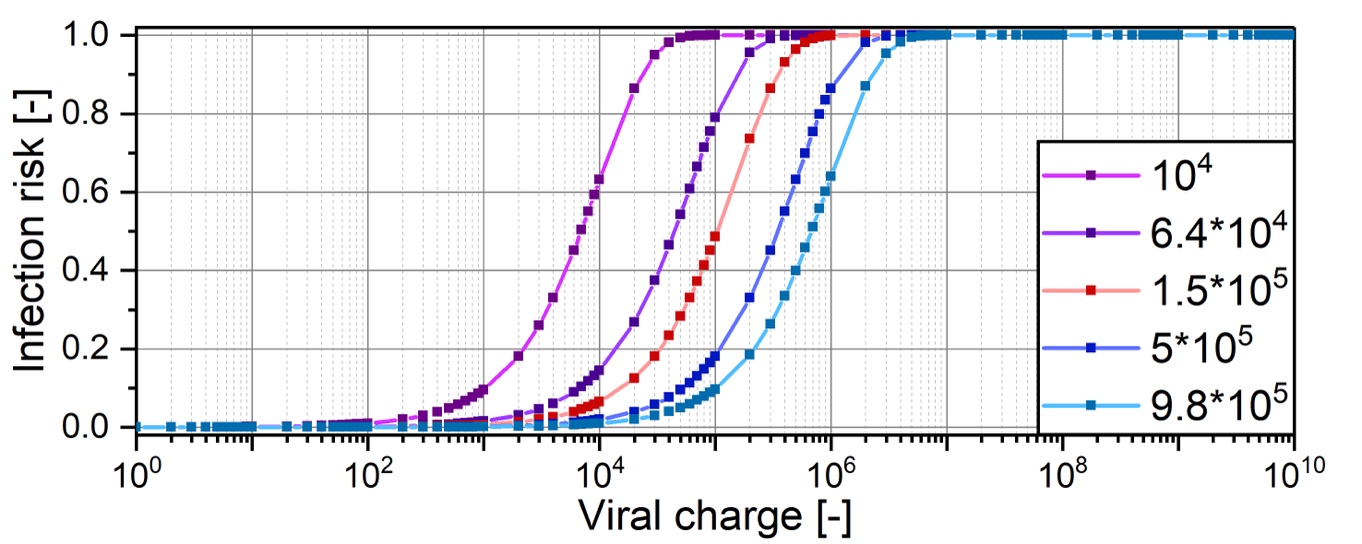 |
| --- |
| 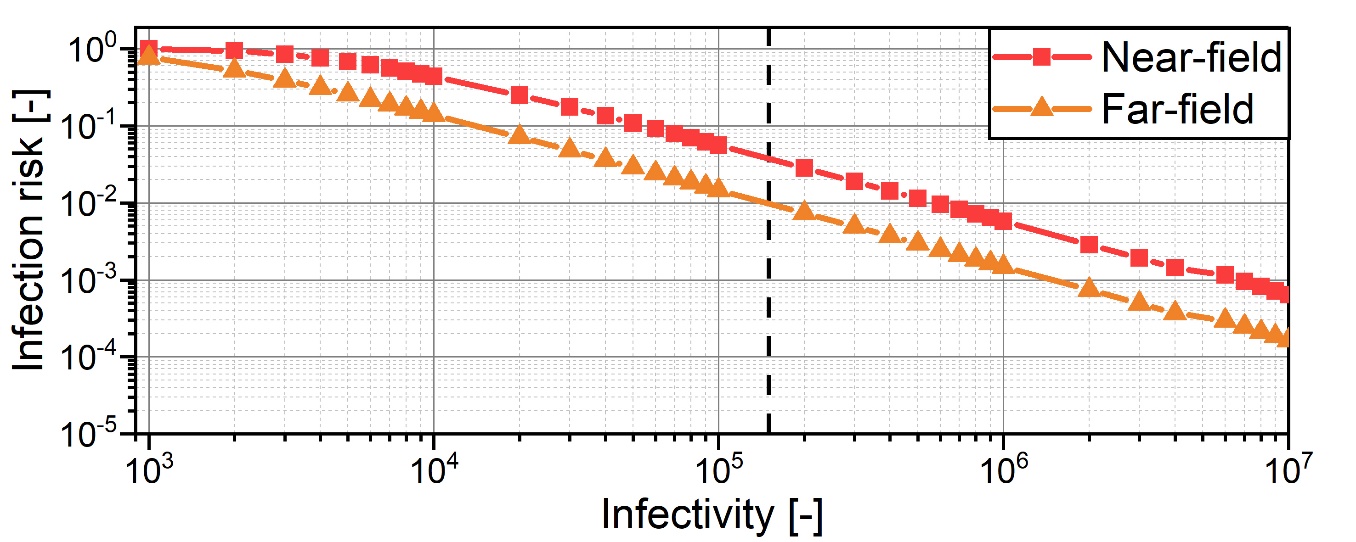 |
| **Figure S21**: Influence of the dose-response coefficient on the infection risk. The dose-response relationship is given for different coefficients is (a), considered the range given by [25], the average value used in this work is highlighted in red. The values of the near-field, far-field, and contact infection risks for different values of the coefficient k are given in (b). |

The **total count** of emitted droplets during the four expiratory activities shows a high inter- and intrapersonal variability, and has a large influence on the model’s output. The results presented in this work were normalized to make the results independent from the initial particle count.

| 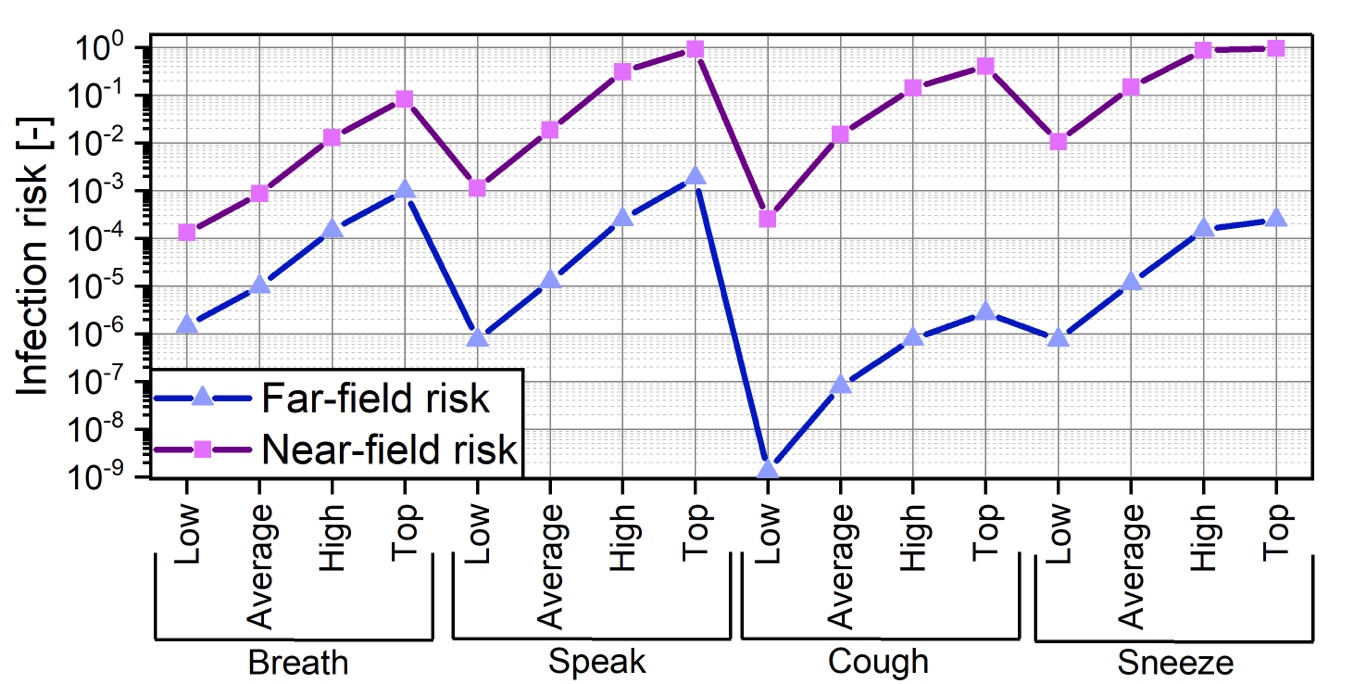 |
| --- |
| **Figure S22**: Infection risk as a function of the different emission scenarios considered. The particle count for the low, average, high, and top levels are based on the values given in **Table S3**. |

The **viral concentration** represents the concentration of viral copies in the liquid fraction of the emitted droplets. It is considered to be equal to the concentration in the lungs and independent from the origin of the droplets. Both assumptions can be challenged as the viral charge concentration in the lungs varies between infected individuals and depends on the infection status and duration [28]. The relation between the viral concentration in the liquid fraction and the viral charge inhaled by the receiver is linear. The emitted droplets are produced by different mechanisms depending on the expiratory activity: during breathing, most of the droplets are generated in the lungs from the ﻿bronchiolar fluid film burst [29], the liquid fraction originating from the lungs. Other expiratory activities generate droplets from the larynx and the mouth where the concentration of viral charges might be different [30]. However, considering the high variability of the of the viral concentration in the lungs, the distinction is not made between the origin of the droplets. The dependence of the output on the concentration of viral copies in the liquid fraction is reduced by normalizing the calculated viral charge and using the size distribution generated during breathing as the reference. The data related to speaking, coughing, and sneezing are calculated relatively to breathing. The influence of the viral concentration on the exhaled viral charge contributing to the near-field, far-field, and contact exposure levels is given in **Figure S23**.

| 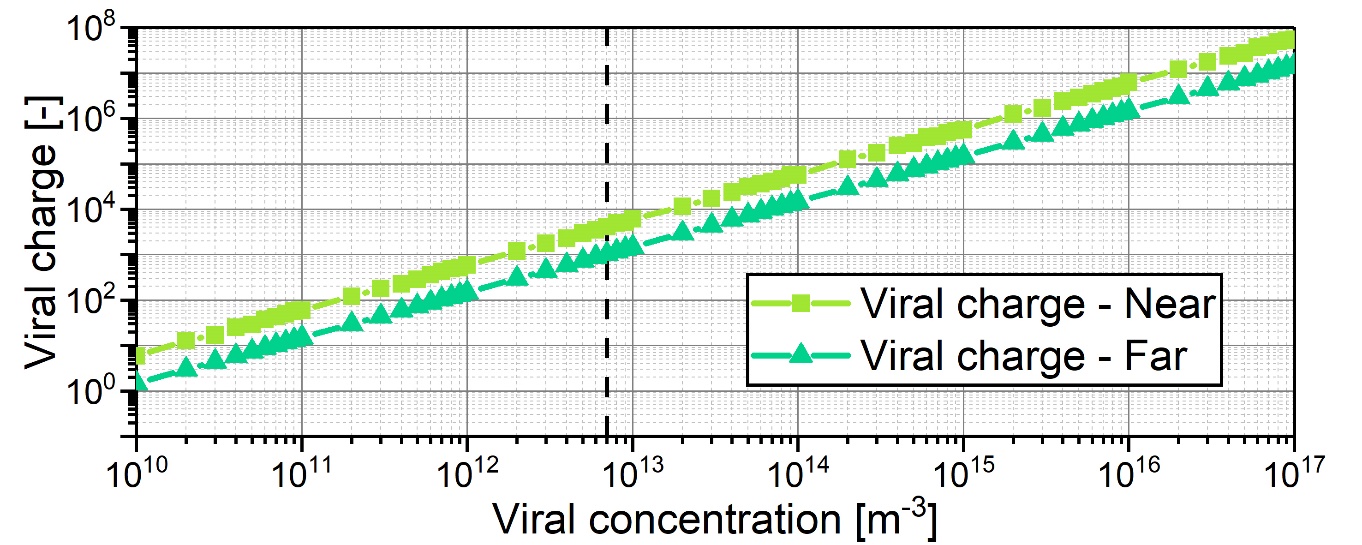 |
| --- |
| **Figure S23**: Viral charge inhaled by a receiver as a function of the viral concentration in the liquid phase of the emitted droplets. The value considered in the comparison of the masks is set to 7x10^12^ copies/m^3^. |

The **viral decay** describes the inactivation of viral copies as a function of the time. This value depends on the type of virus [25] as well as environmental conditions, such as the exposure to UV radiations, the temperature and the relative humidity [31]. The viral decay does not influence the near-field exposure as the time for the calculation is limited to 10 s. The background concentration does depend on the viral decay rate, and changing its value changes the near-field to far-field ratio. However, the relative efficiency of the facemasks or the influence of the leaking fraction are not influenced by a change in the viral half-life. [32] estimates the half-life of aerosolized SARS-CoV-2 at 1.1 to 1.2 hour for a temperature of 22°C and a relative humidity of 40%, close to the conditions simulated in this work (20°C, 50% RH). [31] shows that the presence of UV radiation significantly reduces the half-life. A decrease of the half-life leads to a higher loss through viral decay, which leads to a reduction of the background concentration, responsible for the far-field exposure. The present work considers the hypothesis leading to the maximum infection risk, corresponding to a half-life of 1.1 hour. The influence of the half-life on the infection risk caused by the accumulation of viral charges around the emitter (far-field exposure) is given in **Figure S24**.

| 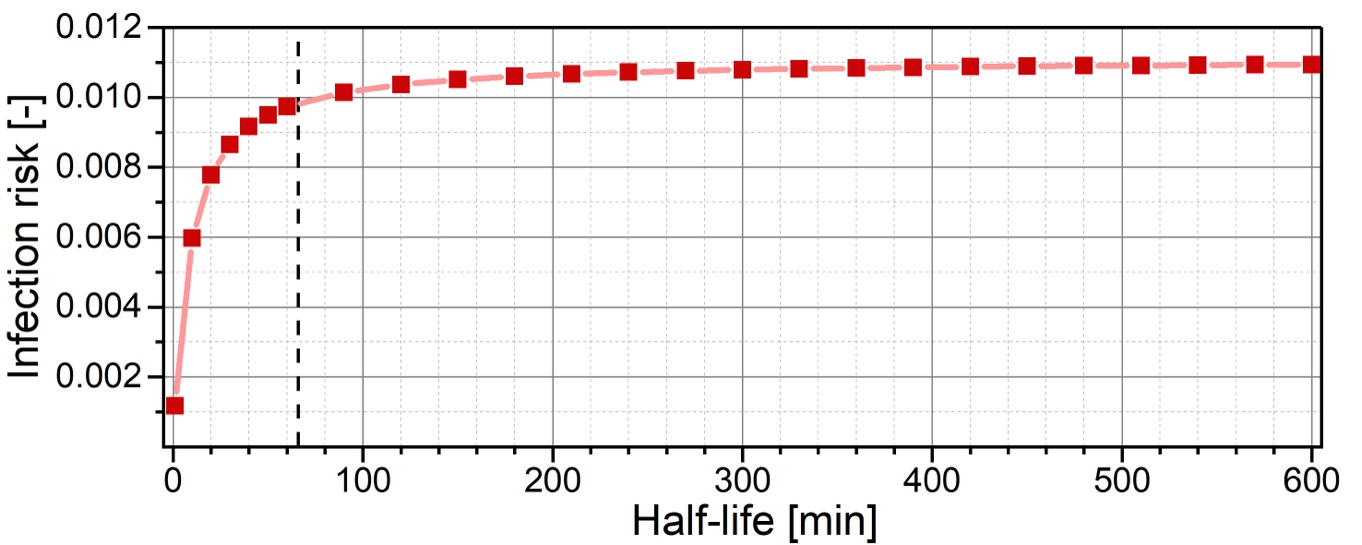 |
| --- |
| **Figure S24**: Far-field infection risk as a function of the half-life of the virus. The near-field risk is not considered here as it is based on a 10 s interaction time and are not significantly affected by the half-life. The dashed line represents the half-life of 1.1 hour considered in the present work. |

The **temperature** influences the filtration efficiency of the facemasks as it changes the diffusion coefficient. The droplets’ trajectories are also influenced by a change in temperature through the thermal velocity and the evaporation rate. However, the influence on the calculated viral charge is negligible (<1%).

The **relative humidity** of the air changes the droplets’ evaporation rate and their final size. For humidity values below 80%, the change in evaporation rate mostly affects larger droplets (larger than 200 microns) and causes a negligible change in the inhaled viral charge (<1%). Humidity values over 80% significantly influence the dry nuclei size and the droplet’s final size, reducing the inhaled viral charge as a higher fraction of the emitted droplets settle on the floor. The influence of the relative humidity on the infection risk is given in **Figure S25**.

| 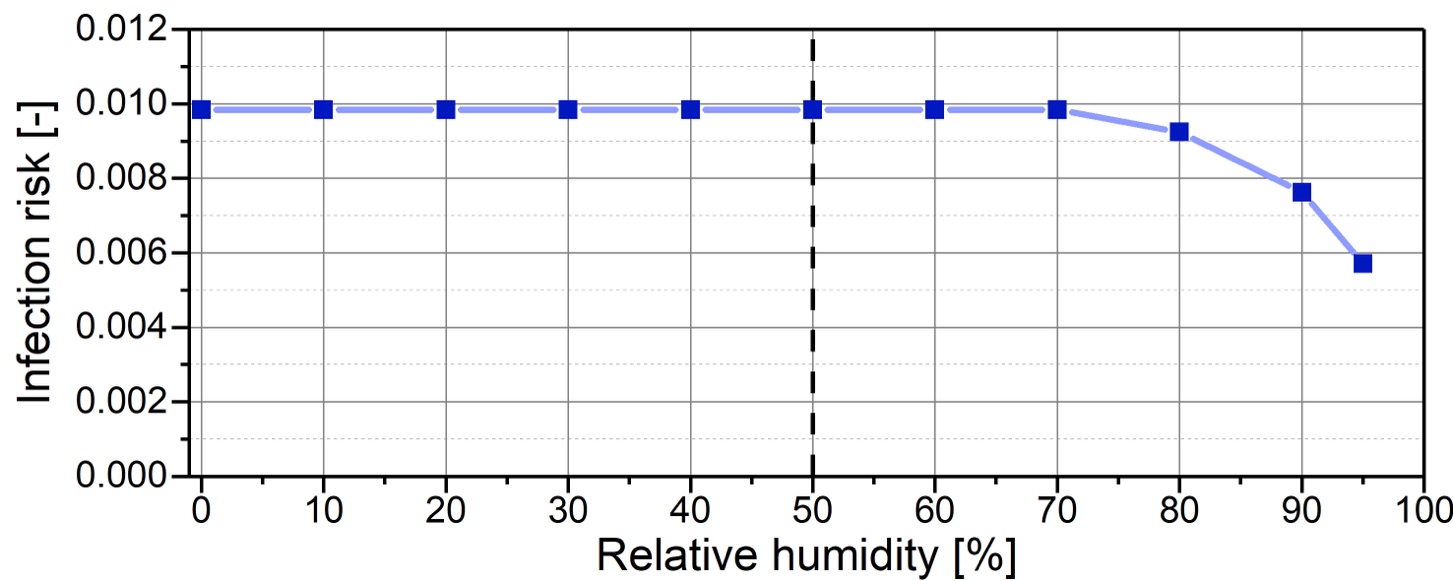 |
| --- |
| **Figure S25**: Infection risk as a function of the ambient humidity. |

The **maximum time** for the calculation of the particles’ trajectories has been set to 10 s. The calculation is interrupted if the maximum time is reached and the particle did not settle on the floor or reach the receiver’s face. The calculation is stopped before the time limit if the particle reaches the floor or the receiver’s face. The value has been chosen to limit the calculation time while reaching a steady state of the system, with all events with a high dynamic already calculated. Slower events (settling and spread of small particles) is not described by the calculation of single particles but in another module by the calculation of the viral charge concentration around the emitter. Changing the maximum time influences the reach rate of the particles (if the limit is too small, particles might not be given enough time to reach the receiver) and the drying time. Both variables have been investigated for a maximum time of 1s, 2s, 5s, 10s, 20s, and 50s. The results are given in **Figures S26** to **S30**.

| 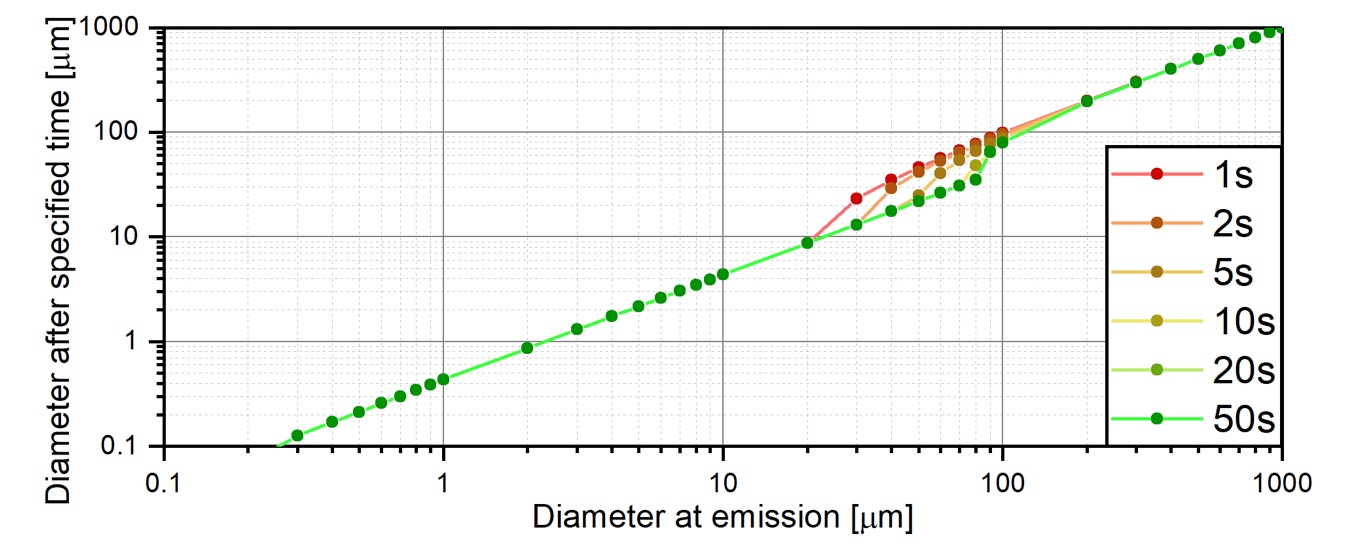 |
| --- |
| **Figure S26**: Influence of the maximum time on the diameter at the end of the calculation |

The diameter at the end of the calculation depends on the time point the calculation for the considered particle is stopped. This parameter does not have a significant influence on small droplets (<20 μm) as they reach their nuclei size in a short time. Larger particles take more time to evaporate and are influenced by the maximum calculation time. Using shorter times, the particles do not have enough time to reach the receiver as it is shown in the next figures and the differences in diameter are linked to differences in reach rate. There is no significant difference between 10 s and 50 s.

| 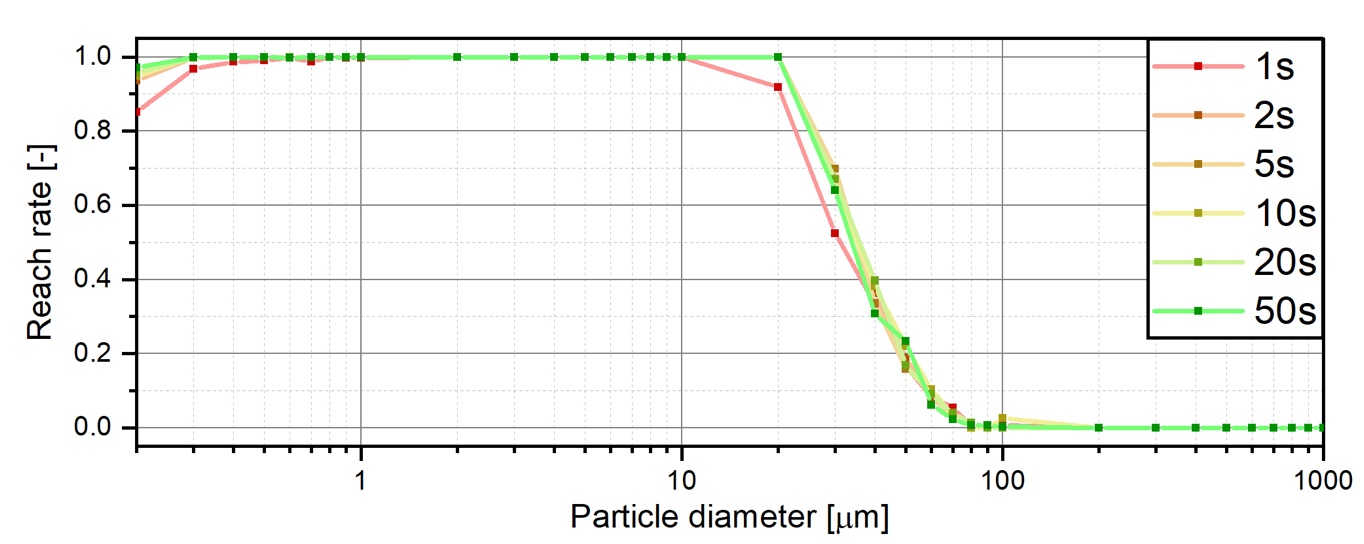 |
| --- |
| **Figure S27**: Influence of the maximum time on the reach rate at 0.25 m without a mask while breathing |

| 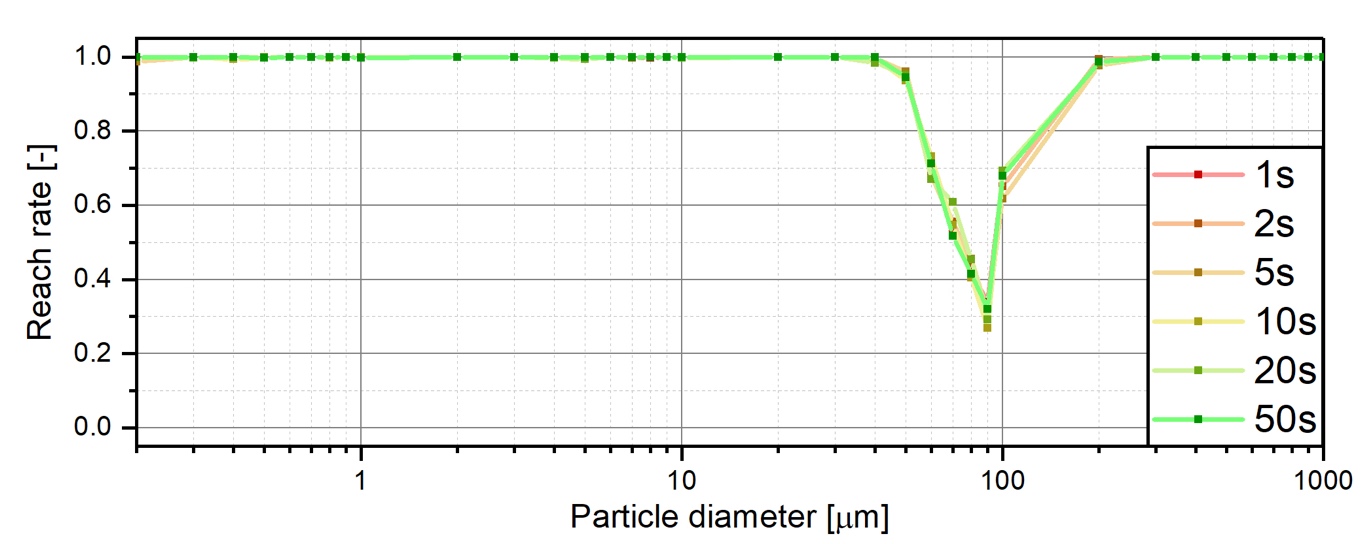 |
| --- |
| **Figure S28**: Influence of the maximum time on the reach rate at 0.25 m without a mask while speaking |

| 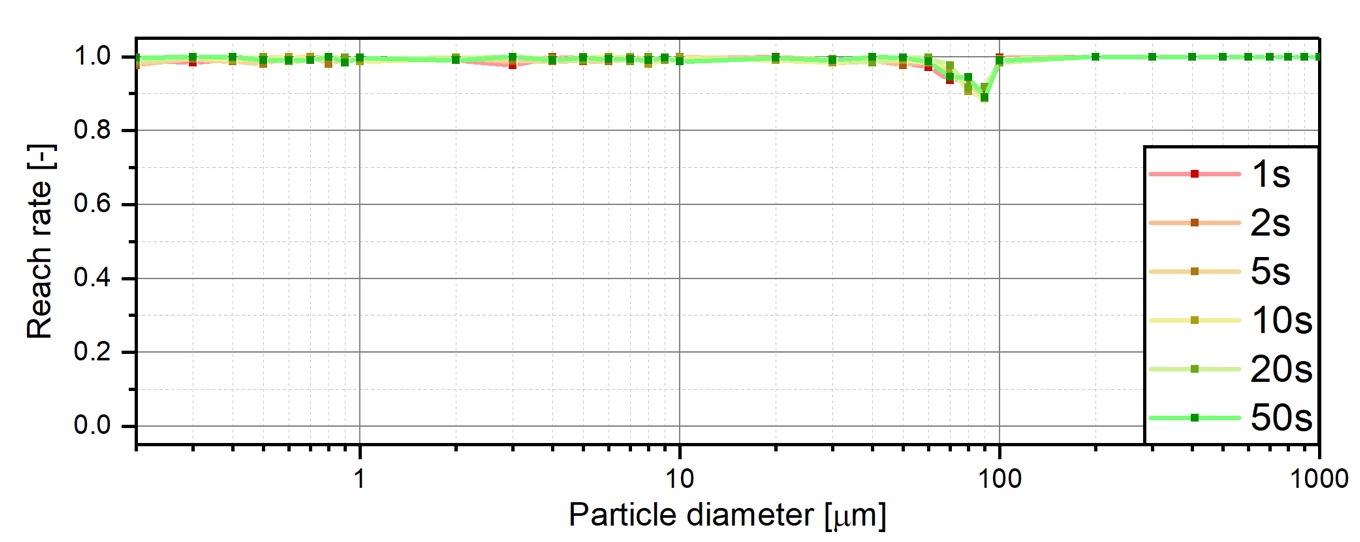 |
| --- |
| **Figure S29**: Influence of the maximum time on the reach rate at 0.25 m without a mask while coughing |

| 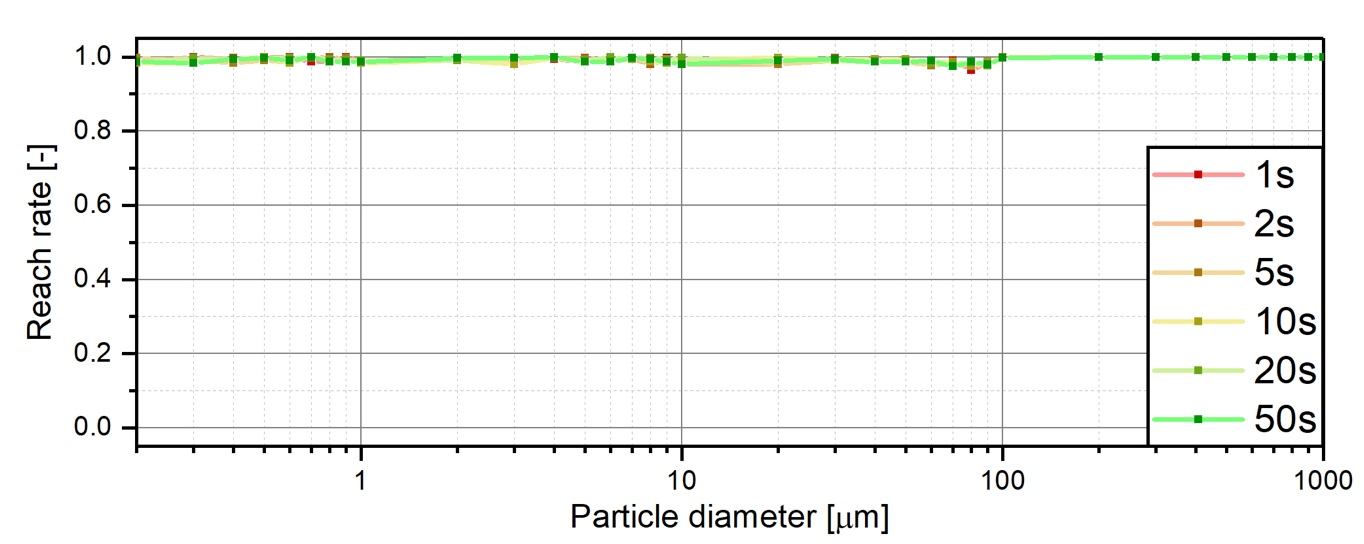 |
| --- |
| **Figure S30**: Influence of the maximum time on the reach rate at 0.25 m without a mask while sneezing |

The reach-rate without a mask increases with increasing initial flow velocity but is not significantly influenced by the maximum calculation time.

1. Model validation and walkthrough

D1 Validation

The model’s outputs were compared with the available literature on the protection efficiency of facemasks. Facemasks reduce the risk of viral transmission, which decreases from 17.4% without a mask to 3.1% with a mask (5.6 times reduction of the risk). N95 facemasks are associated with a larger reduction in the infection risk compared to surgical masks [33]. The study is based on masks worn by the those exposed to infected individuals.

Considering a linear relationship between the infection risk and the inhaled viral charge for a low viral charge [25], the reduction of the viral charge calculated in the present study can be compared to the reduction of the infection risk. Considering that medical staff is trained to properly wear surgical masks, a fit factor according to the minimum required by the standards is realistic (scenario No.1). These assumptions lead to a reduction of infection by a factor 5 with surgical masks and 20 with FFP2 masks, considering an exposure to breathing. The protection efficiency increases when the other expiratory activities are considered, as facemask efficiently block larger particles generated by speaking, coughing, and sneezing generated larger particles compared to breathing. Considering the far-field exposure, the calculated reduction of the infection risk is 10 times for a surgical mask and 40 times for a FFP2 considering speaking, and respectively 5.6 times and 24 times for coughing, and 10 times and 50 times for sneezing.

|  | | **Surgical** | **FFP2** | **Ref. [33]** |
| --- | --- | --- | --- | --- |
| **Breath** | **Ratio Near** | 0.198 | 0.049 | 0.178 |
|  | **Ratio Far** | 0.188 | 0.042 |  |
| **Speak** | **Ratio Near** | 0.007 | 0.002 |  |
|  | **Ratio Far** | 0.107 | 0.025 |  |
| **Cough** | **Ratio Near** | 0.002 | 0.001 |  |
|  | **Ratio Far** | 0.185 | 0.043 |  |
| **Sneeze** | **Ratio Near** | 0.007 | 0.002 |  |
|  | **Ratio Far** | 0.106 | 0.024 |  |
| **Table S12**: Relative reduction of the viral charge compared to the risk reduction estimated in [33] | | | | |

D2 Walkthrough

This second part presents the detailed inputs and outputs of each module through one example: the emitter is coughing once and breathing for one hour. The protection efficiency of the mechanical FFP2 mask used as respiratory protection and source control is calculated. The considered leaking situation is the scenario No.1, with the leaking flow according to the standards. The emitter and receiver are separated by 0.5 m. The details for the calculation are presented following the order of the modules described in Section B.

D2.1 Initial data

- Facemask: FFP2, worn by both the emitter and the receiver
- Distance: 0.5 m
- Expiratory activity: coughing followed by breathing

The size distributions (size at emission and count per size bin) are extracted from text files depending on the initial parameters. A resolution of 10 points per decade is used throughout the calculations. The emission velocities are defined together with the expiratory activities, in this case: 1.6 m/s for breathing and 11.7 m/s for coughing. The initial size distributions considered in the present example are given in **Figure S31**.

| 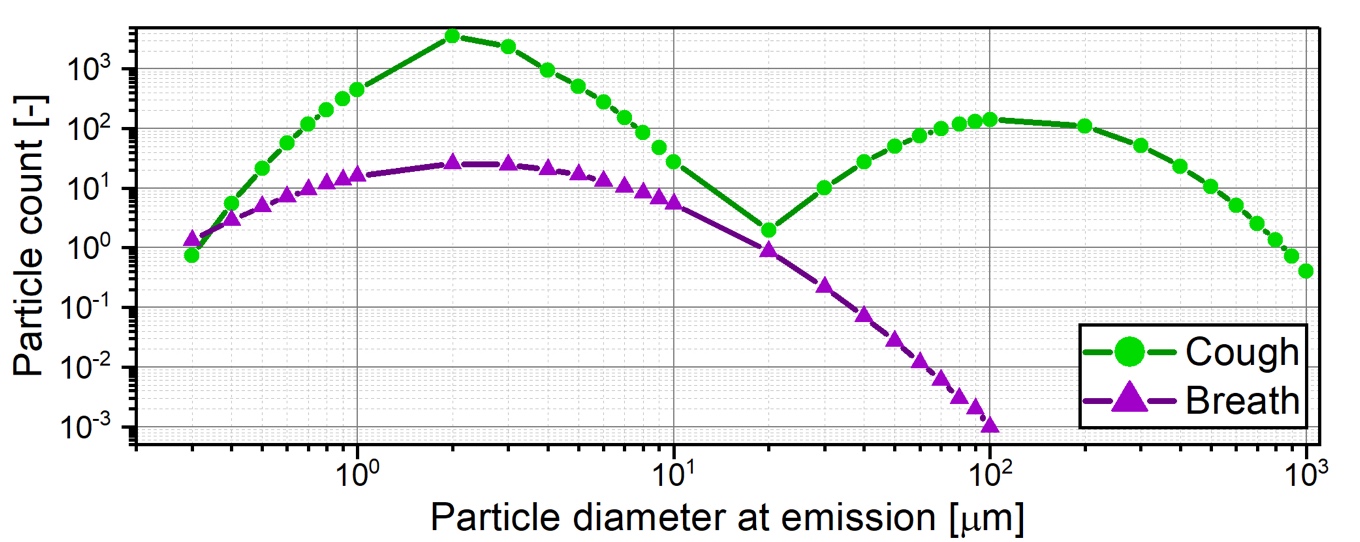 |
| --- |
| **Figure S31**: Initial size distribution considered in the present example. The data is not normalized and corresponds to the emitted number of particles in each size class for breathing (corresponding to one minute emission) and coughing (corresponding to one cough). The size resolution is 10 points per decade. |

D2.2 Emitter’s facemask including leakage

The mask parameters (solidity, thickness, fiber size distribution and charge density) are extracted from text files. The mask and leaking flows are calculated depending on the calculation method chosen by the user. In this example, the leaking flow is directly set to 5% of the emitted flow. Other options include targeting a pressure drop or entering a gap size. The desired pressure drop is reached by modifying the gap size. The flows and corresponding face velocity are calculated considering a mask active diameter of 10 cm.

- Data for breathing:
  - Emitted flow = 1.6*π(2x10^-2^/2)^2^ = 5.027x10^-4^ m^3^/s (based on the mouth diameter and the initial flow velocity)
  - Mask flow: 95%*5.027x10^-4^ = 4.77x10^-4^ m^3^/s
  - Leaking flow: 5%*5.027x10^-4^ = 2.51x10^-5^ m^3^/s
  - Face velocity: 4.77x10^-4^/((10^-1^/2)^2^*π)=0.0608 m/s
- Data for coughing:
  - Emitted flow = 11.7*π(2x10^-2^/2)^2^ = 3.7x10^-3^ m^3^/s (based on the mouth diameter and the initial flow velocity)
  - Mask flow: 95%*3.7x10^-3^ = 3.5x10^-3^ m^3^/s
  - Leaking flow: 5%*3.7x10^-3^ = 1.85x10^-4^ m^3^/s
  - Face velocity: 0.44 m/s

The face velocity is used to calculate the filtration efficiency by diffusion, inertial impaction, interception, and interception of diffusing particles, combined to calculate the efficiency of the mask.

The cut-off size for the particles able to follow the leaking flow is calculated based on the initial flow velocities. Particles smaller than the cutoff size follow the leaking flow, and particles larger than the cut-off size stay in the mask flow. The calculated cut-off size was 8 μm for coughing, and 20 μm for breathing. The filtration efficiency and the fraction of the particles leaving through the leakage are corrected by the corresponding volumetric flow fractions. A threshold sets the penetrating fraction to zero if the number of penetrating particles is less than one over the duration of the interaction. This correction is applied to avoid an overestimation of the contribution of large particles with a low penetration coefficient but a high viral load to the inhaled viral charge. The fraction of particles released from the emitter’s mask (filtration and leakage) is presented in **Figure S32**.

| 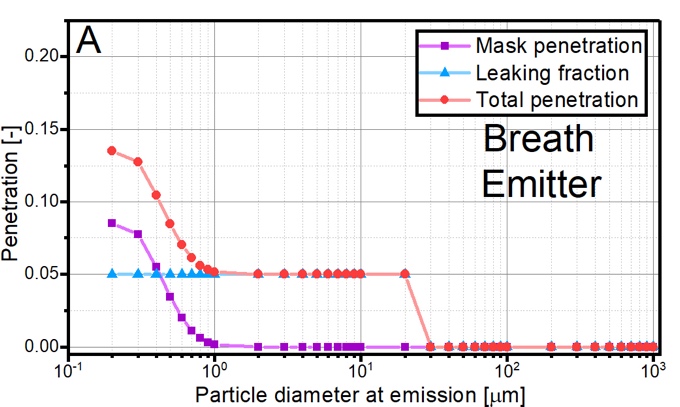 | 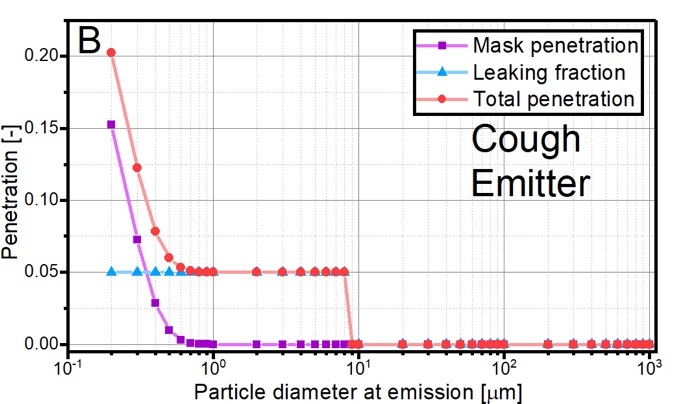 |
| --- | --- |
| 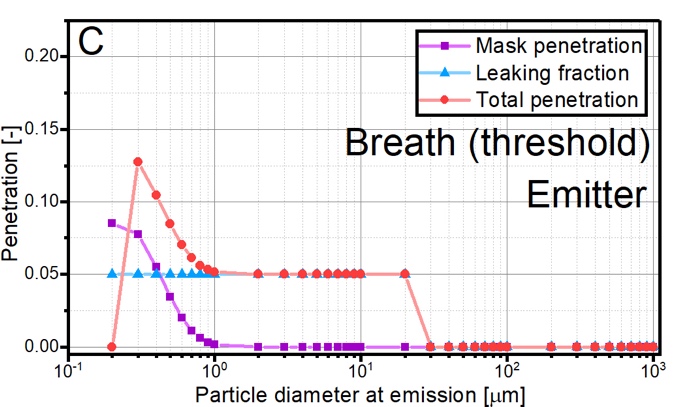 | 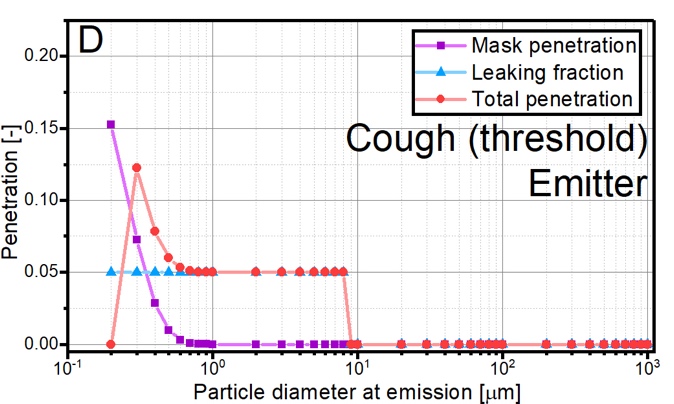 |
| **Figure S32**: Fraction of the particles released from the emitter’s mask, showing the breakdown into the mask penetration and leaking fraction. Breathing and coughing are shown without the threshold (A and B) and with the threshold (C and D), showing the removal of one datapoint at 200 nm. | |

D2.3 Particles transport and evaporation

This module runs four times in the current example: for each expiratory activity (breathing and coughing here), it runs with mask and without the emitter’s mask in order to consider the impact of the emitter’s mask on the initial velocity of the droplets. The droplets are generated at different initial positions from a circle representing the mouth/nose. A fraction of the emitted droplets, corresponding to the previously calculated leaking fraction, is generated with a velocity corresponding to the leak velocity (calculated at 0.08 m/s for breathing and 0.59 m/s for coughing) and directed upward to simulate a gap around the nose. The sizes of the particles upon reaching the receiver’s mask are given in **Figure S33** for breathing and coughing. They are compared with the dry nuclei diameter.

| 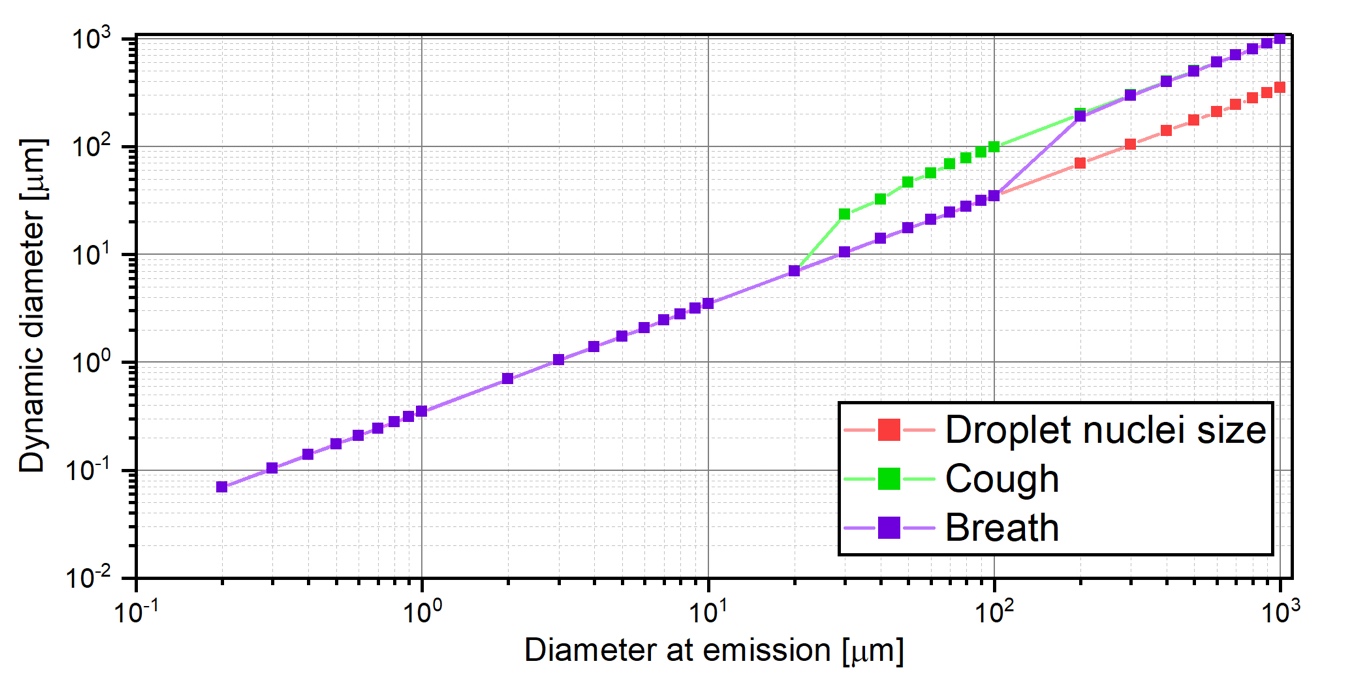 |
| --- |
| **Figure S33**: Comparison of the different diameters considered for the calculation. The droplet nuclei size (red curve) is the reference for the calculation of the dynamic diameter. The calculation of the near-field viral charge (receiver’s mask and lung deposition modules) is based on the diameter at the time the particle reaches the receiver’s mask (green and blue curves). The calculation of the near-field is based on the assumption that the particles are fully evaporated and is based on the nuclei size. |

D2.4 Accumulation of viral charges

The accumulation of viral charge leads to the calculation of the far-field exposure level. The output is the concentration of viral charges in a defined space around both the emitter and the receiver, considering a homogenous distribution of the viral charges within the considered space. The module takes into the breathing pattern, set to 15 respiratory movements per minute with both the inhalation and exhalation taking 2s. The viral charge is scaled down to the volume of air inhaled (tidal volume = 0.77 L similar to the value used in the lung deposition module). **Equation 34** is used to calculate the evolution of the concentration of viral charges over the time (considering an interaction time of 1 hour). The viral shedding for recurrent activities is based on the number of emitted particles corresponding to one minute and scaled down to the required interaction time. For single activities (coughing, sneezing), the shedding is set to zero and the emitted viral charges are set as initial concentration. The evolution of the accumulated viral charges as a function of the interaction time is given in **Figure S34**. This dataset is combined with the breathing pattern (inhalation time and volume) to calculate the total viral charge entering the receiver’s lungs over the interaction time. The data is shown in **Figure S35**.

| 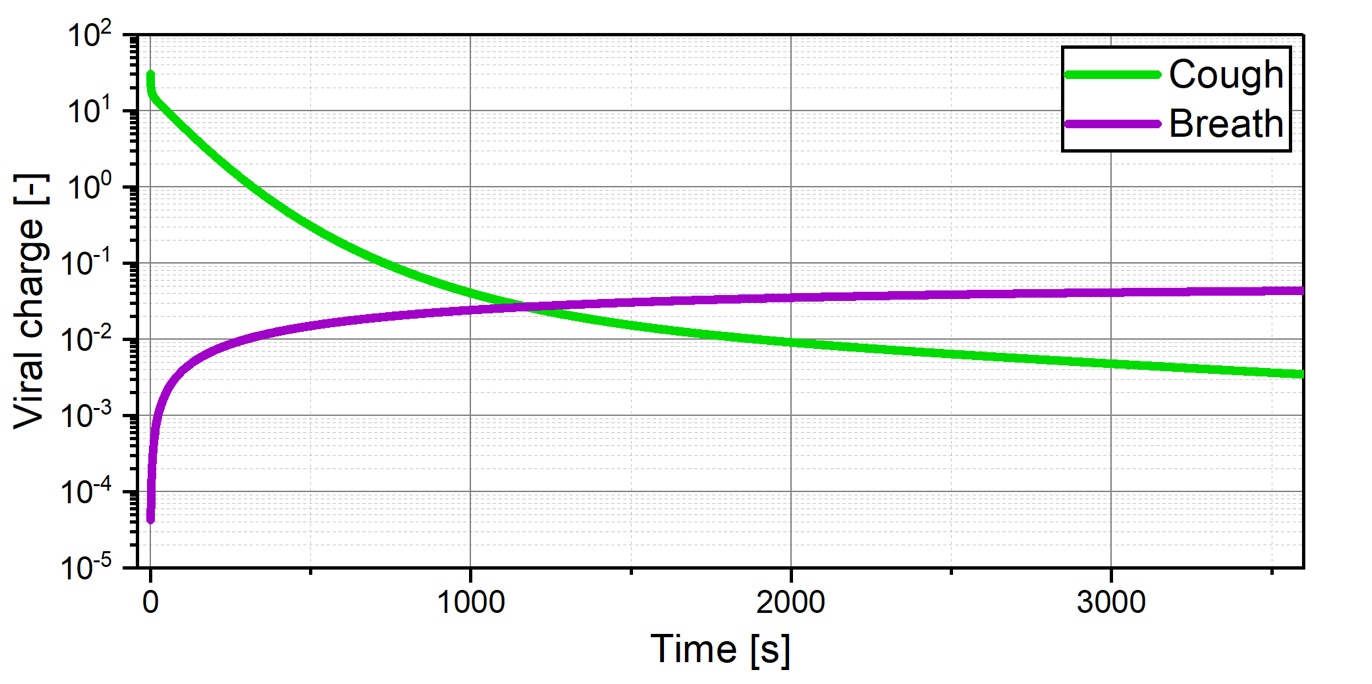 |
| --- |
| **Figure S34**: Evolution of the viral charge as a function of the time, considering coughing as a single activity (green curve) and breathing as a recurring activity (breath). |

| 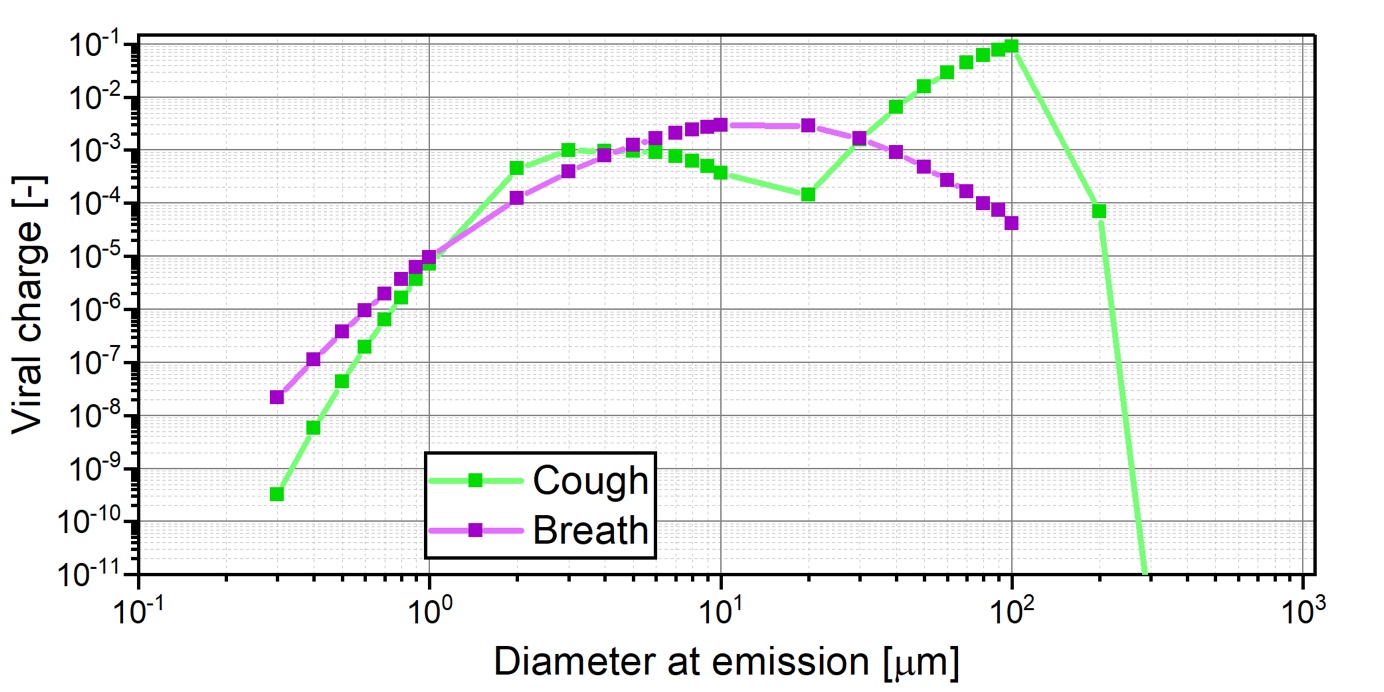 |
| --- |
| **Figure S35**: Contribution of each size to the transmitted viral charge. The lung deposition module and emitter’s/receiver’s facemasks are not applied at this stage. |

D2.5 Receiver’s facemask including leakage

The penetration of the particles through the receiver’s mask is calculated in a similar way than the emitter’s mask. The inhaled flow does not depend on the expiratory activity and is set to 30 L/min, corresponding to a constant face velocity of 0.0605 m/s in the current example (95% of the volumetric flow of 30/60000=5x10^-4^ m^3^/s divided by the active mask surface of π(10^-1^/2)^2^ m^2^). The inward leakage is modelled by another impactor, whose cut-off size is based on the mouth/nose diameter and the inhalation velocity (1.6 m/s, corresponding to 30 L/min flowing through the 2 cm diameter opening of the mouth/nose). The calculations are based on the diameter at the time they reach the mask, corresponding to the dry nuclei size for particles ≤ 200 μm, larger particles did not have the time to fully evaporate. This approximation does not influence the calculation of the far-field exposure level, as particles larger than 200 μm have a high settling velocity and have a negligible contribution to the far-field exposure level. The total particle penetration (mask and leakage) is shown in **Figure S36**.

| 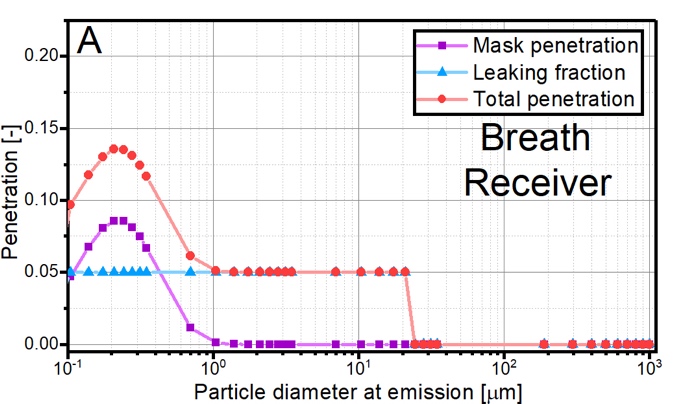 | 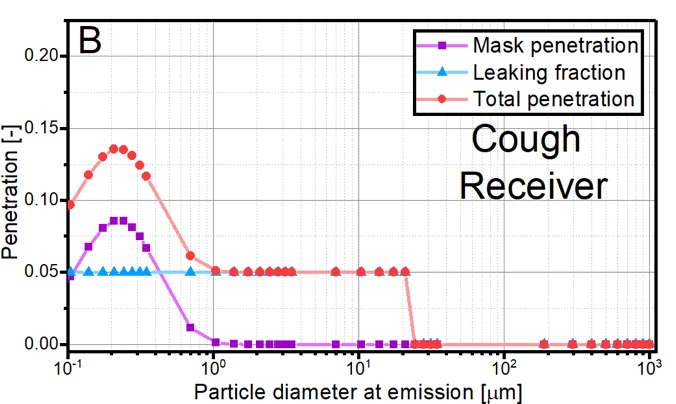 |
| --- | --- |
| 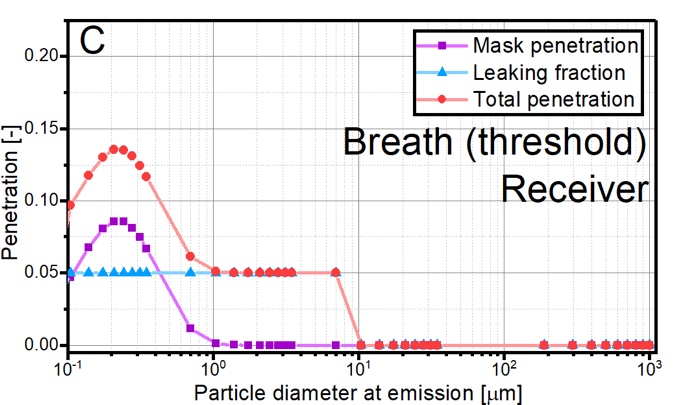 | 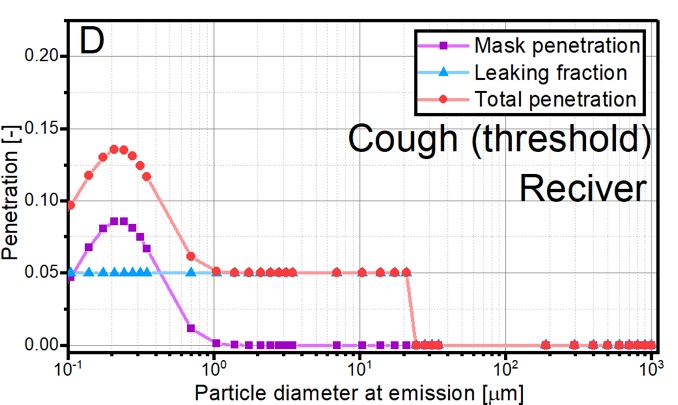 |
| **Figure S36**: Fraction of the particles penetrating the receiver’s mask, showing the breakdown into the mask penetration and leaking fraction. Breathing and coughing are shown without the threshold (A and B) and with the threshold (C and D). | |

D2.6 Lung Deposition

The lung deposition module is based on the lung data stored in a text file and describing the geometry of the lungs, the flow velocities and the corresponding Reynolds numbers. The dimensions are adapted to a tidal volume of 0.77 L. The inhalable fraction and deposition in different parts of the respiratory system is given in **Figure S8**. The lungs parameters and the inhalation flowrate are kept constant in the present work.

D2.7 Calculation of the inhaled viral charge

The outputs from the different modules are combined to calculate the near-field and far-field exposure levels. The results are given in **Table S13**, the distribution of the exposure levels in the different size classes is given in **Figure S37**.

| **No masks** | Near-field exposure | 914.29 |
| --- | --- | --- |
|  | Far-field exposure | 0.0617 |
| **Only the emitter has a mask** | Near-field exposure | 0 |
|  | Far-field exposure | 0.0018 |
| **Only the receiver has a mask** | Near-field exposure | 2.9918 |
|  | Far-field exposure | 0.0018 |
| **Both wear masks** | Near-field exposure | 0 |
|  | Far-field exposure | 8.8064x10^-5^ |
| **Table S13**: Calculated exposure levels | | |

| **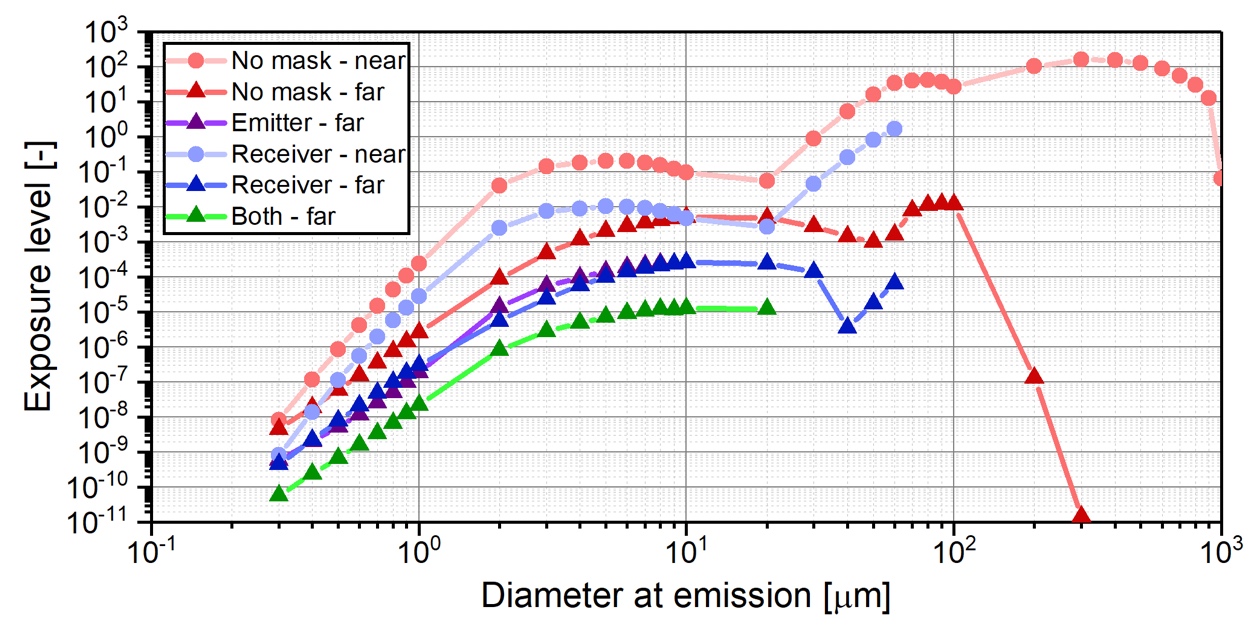** |
| --- |
| **Figure S37:** Size-resolved exposure level |

1. Additional data

E1 Additional data and discussion on the protection efficiency of facemasks considering a no-leakage scenario

The distributions of viral charge in the five size classes of the inhaled particles generated by the four expiratory activities are given in **Figure S38a**. The far-field exposure was mainly associated with particles below 30 μm at emission for breathing and partially with particles below 100 μm in the other cases.


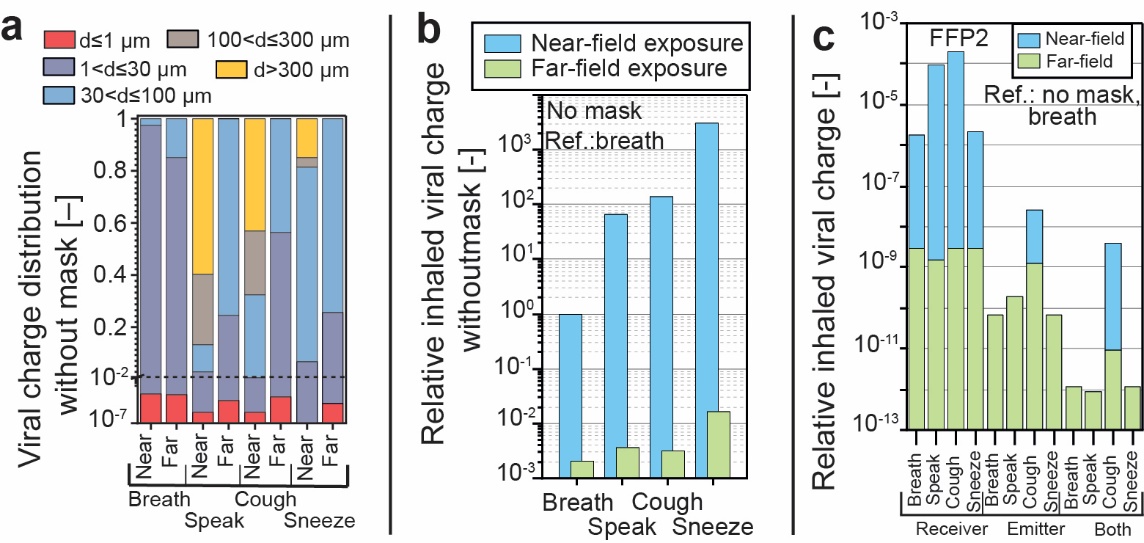


**Figure S38**: Additional data to the protection efficiency with a mask considering a no leakage scenario (**Figure 4**). The relative contribution of the five different size classes to the inhaled viral charge without a mask is given in (**a**). The relative viral charge inhaled considering the four emission scenarios and the contributions of the near-field and far-field exposure levels are given in (**b**) without a mask, and in (**c**) with a FFP2 mask. The data were normalized using the near-field exposure from breathing as a reference in (**b**) and the total exposure due to breathing without a mask as reference in (**c**).

The near-field exposure generated from breathing had a similar composition as the far-field exposure, while particles larger than 100 μm became dominant for speaking and coughing. The contribution of particles between 30 and 100 μm significantly increased and became prevailing in the case of sneezing. The increased reach-rate between 30 and 100 μm, due to a higher initial velocity (previously shown in **Figure** **4b**), and a larger emission of viral charges (**Figure 2**) explain the rising contribution of particles between 30 and 100 μm to the near-field exposure. Detailed data are given in **Figures S39** to **S43**. The comparison of the near-field and far-field exposure levels for the four expiratory activities is given in **Figure S38b** without a mask and **Figure S38c** with a FFP2 mask. Sneeze induced significantly higher near- and far-field exposure than other activities, which was linked to the increase of the fraction of the viral charge carried by particles between 30 and 100 μm shown in **Figure S38a**. The coughing emission scenario was based on one cough followed by one hour nose breathing and the speaking scenario was composed of 30 minutes speaking combined with 30 minutes nose breathing, generating a volume-based emission ratio between both activities of 1.6 considering one hour exposure. Coughing generated larger particles than speaking, leading to a higher loss of particles due to gravitational settling, and therefore a limited accumulation of viral charges around the emitter, explaining the lower far-field exposure. The comparison of the near-field to far-field ratio in **Figure S38c** shows that the contribution of the near-field exposure was negligible due to the filtration of the exhaled air when the mask was used as source control on the emitter. It is worth noting that only coughing generated a non-negligible near-field exposure when the mask was worn by the emitter. Coughing led to an increase of the initial velocity and number of emitted particles compared to breathing and speaking, thus higher reach-rate and amount of emitted viral charge, and eventually lead to a higher near-field exposure. Sneezing provided even higher amount of emitted viral charge than coughing, however, the higher sizes of the emitted particles limited their penetration and therefore the near-field exposure. Therefore, coughing led to a higher near-field exposure level than sneezing in the scenarios with masks.

The fraction of particles between 30 and 100 μm significantly increases in both the near-field and far-field exposure levels when the expiratory activity moves from breathing to sneezing, as shown in Figure 4b.

The data presented in **Figure S39** show an increase of the reach-rate for every particle size as the initial velocity increases. This change is more significant for particles larger than 30 μm.

| **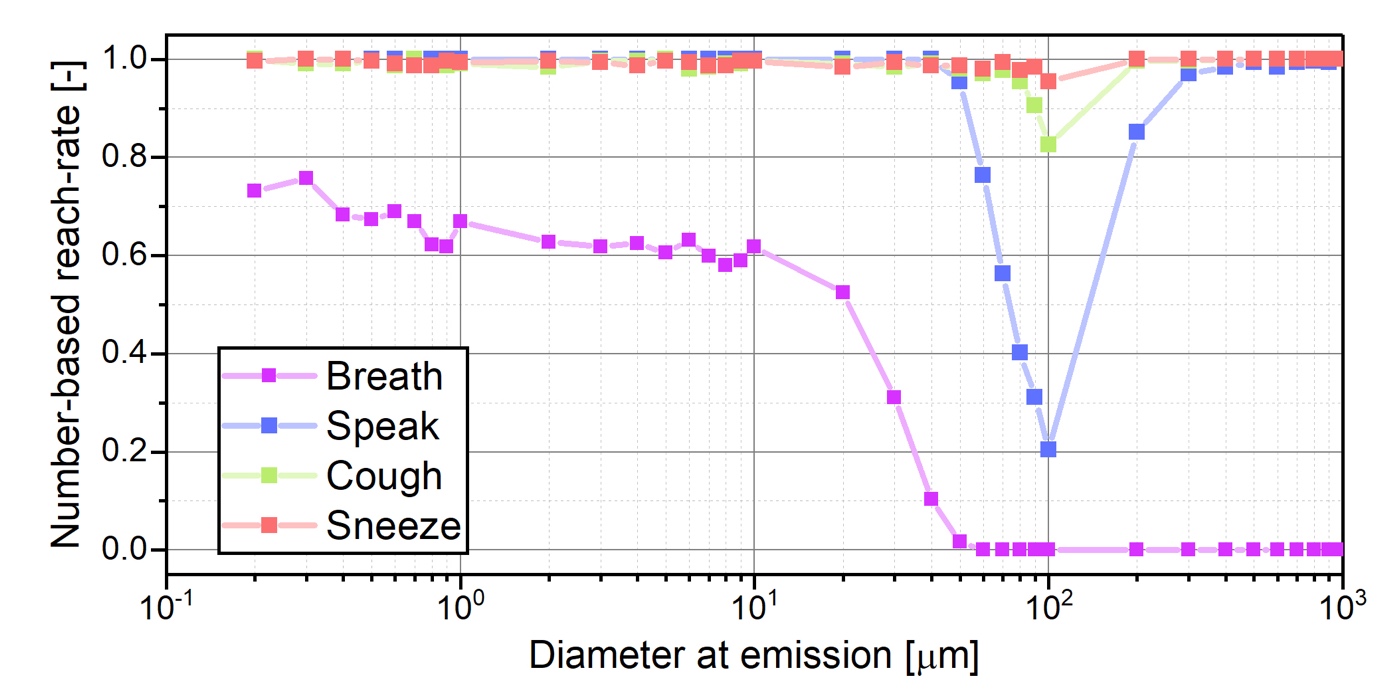** |
| --- |
| **Figure S39:** Number-based reach-rate of the particles emitted by the four expiratory activities (speaking, coughing, and sneezing are considered alone, without the additional breathing to isolate the influence of the initial velocity). The receiver is placed 0.25 m away from the emitter and none of the individuals wear a mask. |

The data presented in **Figure S40** shows the inhaled viral charge for the different expiratory activities without masks, the normalized data is given in **Figure S41** to highlight the relative contribution of each size class to the overall exposure level.

| **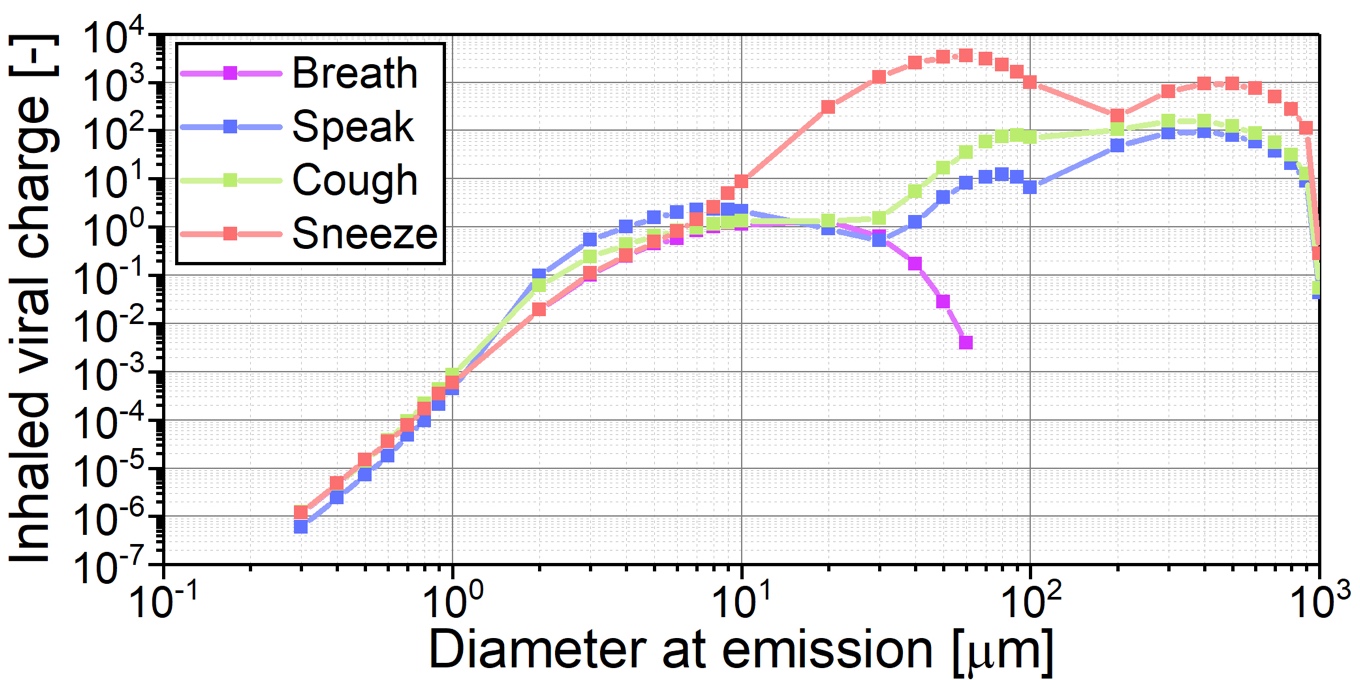** |
| --- |
| **Figure S40:** Near-field exposure level considering the four expiratory activities (breathing is added to speaking, coughing, and sneezing). The receiver is placed 0.25 m away from the emitter and none of the individuals wear a mask. |

| **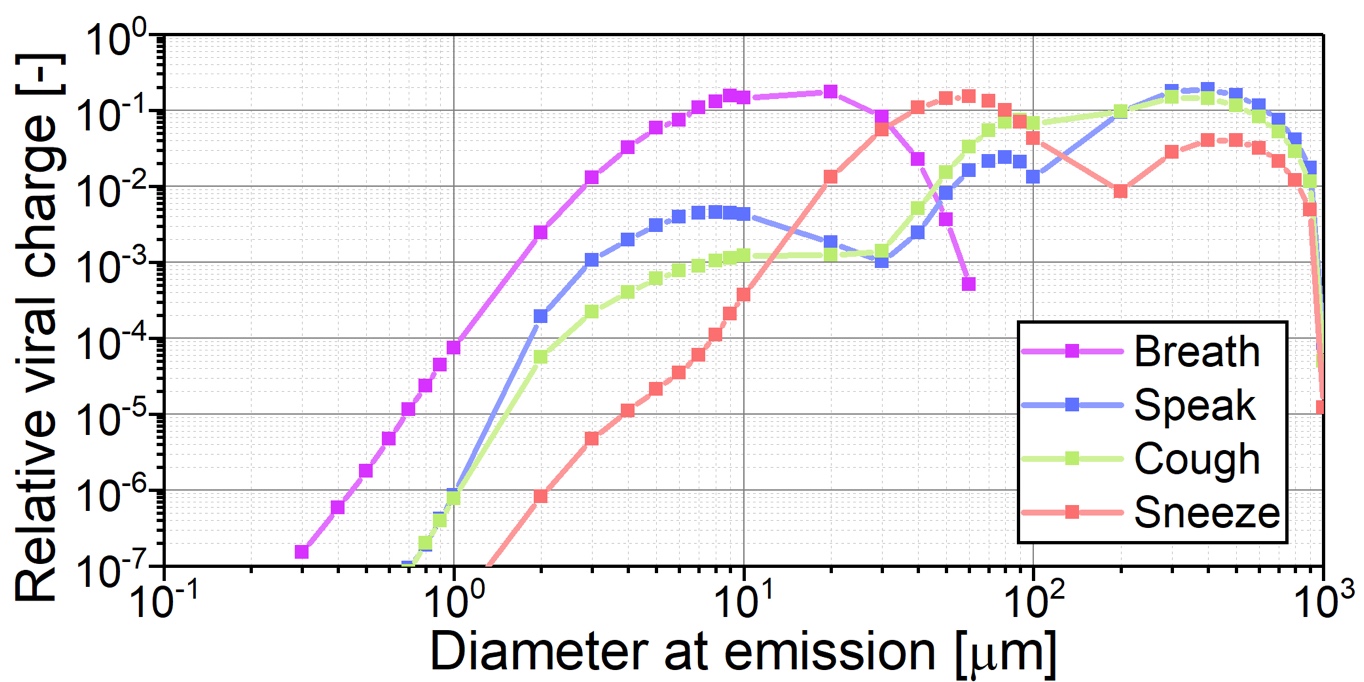** |
| --- |
| **Figure S41:** Relative near-field exposure level, taking the total emitted viral charge for each activity as reference to highlight the contribution of each size class to the overall exposure level. |

The data given in **Figure S40** is to be compared with the data in **Figure S38c** and the data in **Figure S41** reflects the data in **Figure S38b**.

An increase of the viral charge carried by particles in the 30-100 μm range was also calculated for the far-field exposure level. The data is given in **Figure S42** and **Figure S43** (relative values).

| 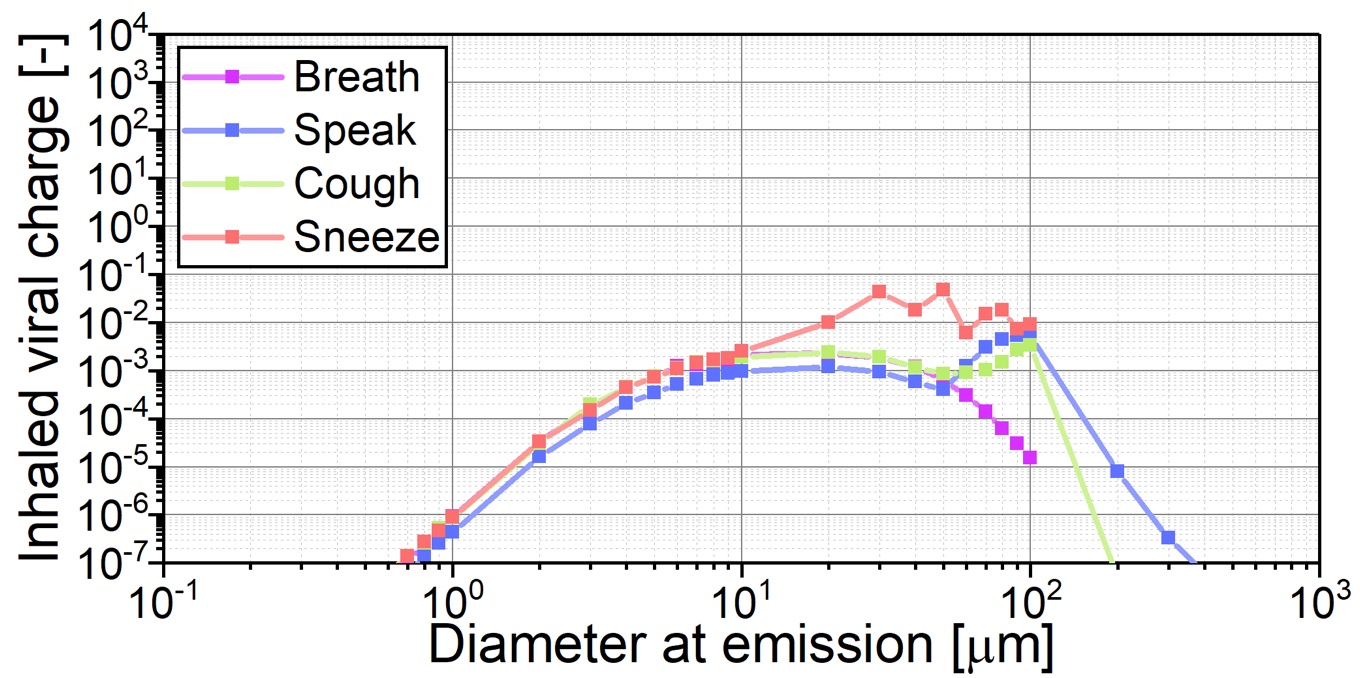 |
| --- |
| **Figure S42**: Far-field exposure level considering the four expiratory activities (breathing is added to speaking, coughing, and sneezing). The receiver is placed 0.25 m away from the emitter and none of the individuals wear a mask. |

| 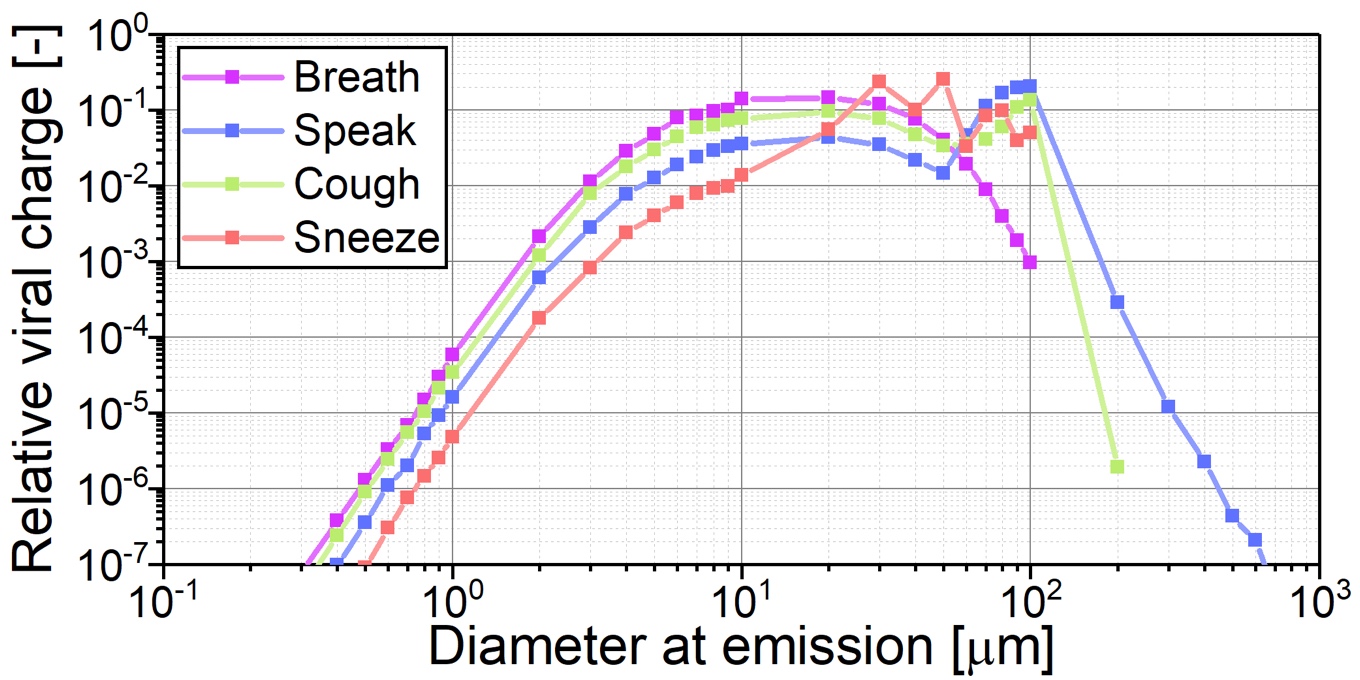 |
| --- |
| **Figure S43**: Relative far-field exposure level, taking the total emitted viral charge for each activity as reference to highlight the contribution of each size class to the overall exposure level. |

It is worth noting that the contribution of the breathing is the main source of viral charges carried by particles smaller than 20 μm.

The near-field exposure level is significantly higher for coughing compared to the other expiratory activities, as shown in Figure 4d. The reach-rate presented in **Figure S44** shows that no particles reach the receiver for breathing and speaking. The data shown in **Figure S45** indicates that the higher initial velocity of the particles released from a sneeze causes an increase of the filtration efficiency for particles larger than 250 nm, as the MPPS shifts toward smaller particles, explaining the absence of a near-field exposure from sneezing.

| 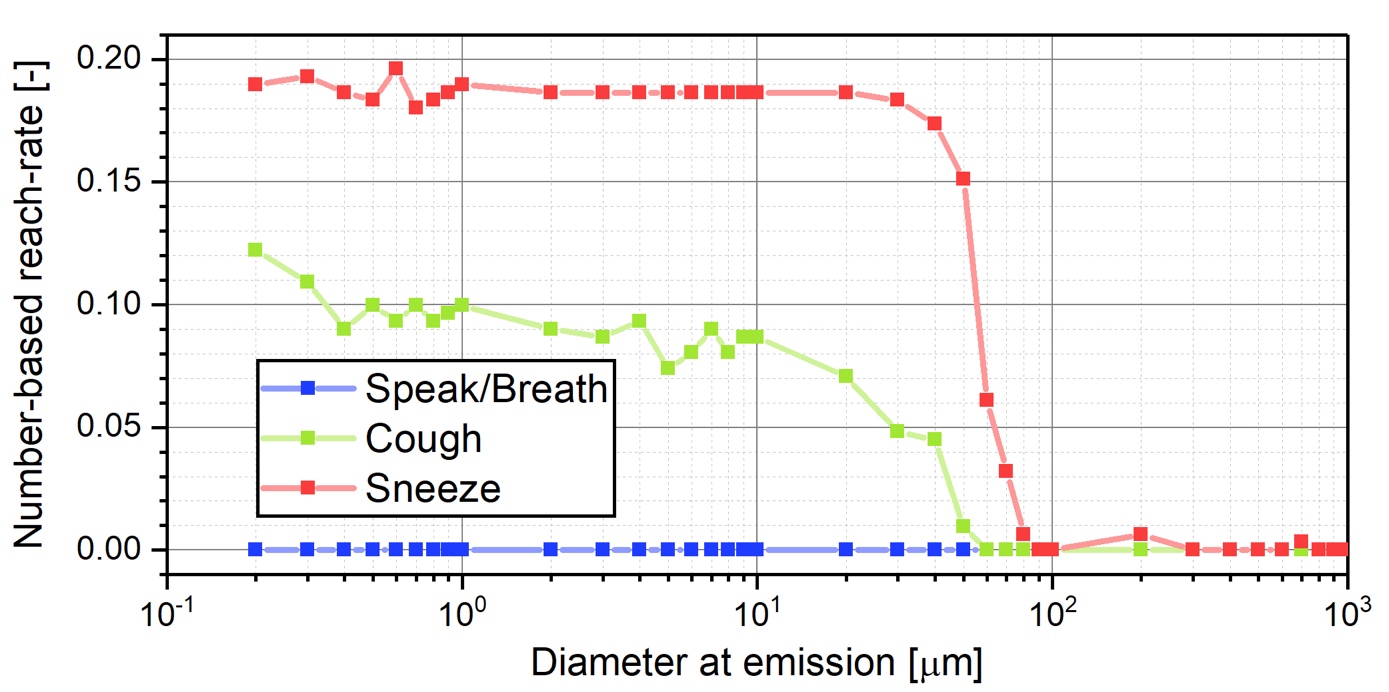 |
| --- |
| **Figure S44**: Number-based reach-rate for the four expiratory activities considering a receiver located 0.25 m away from the emitter. The emitter wears a perfectly fitted FFP2 mask (no leakage). |

| **** |
| --- |
| **Figure S45:** Filtration curve of a perfectly fitted FFP2 facemask considering the four expiratory activities. It is worth noting that the mask has a higher penetration (>9% for breathing at 200 nm) than the maximum allowed by the EN 149 standard (6%) due to the higher flow velocity in the expiratory activities (6.1 cm/s for breathing) compared to the test velocity (5 cm/s) |

E2 Discussion on the differences between the different types of FFP masks in the different leaking scenarios (complementary discussion to Figure 6)

Notable differences in protection efficiency considering the two leaking scenarios based on pressure drops (No.3 and No.4) appeared in **Figure 6** among the FFP masks and reflected their different breathing resistances: at equal pressure drop, the leaking flow was lower for the FFP1 mask compared to the FFP3 mask (modelled with a pressure drop 45% higher than the FFP1). The exposure level with a FFP3 mask used as source control was 65% higher compared to a FFP1 mask in scenario No.3 and 4% in scenario No.4 (respectively 58% and 5% when the masks were used as respiratory protection). The FFP2 mask based on mechanical filtration generated the lowest protection efficiency (face-shield excluded) in scenario No.3. The FFP2 mask after loss of its electrostatic charges provided the lowest protection in scenario No.4 with the mask on the receiver. The role of the pressure drop in the protection efficiency is further highlighted by the comparison between the three FFP2 mask. In both scenarios based on equal leaking flows (No.1 and No.2), the FFP2 mask after loss of its electrostatic charges provided the lowest protection (the average inhaled viral charge was 91% higher compared to the FFP2 in scenario No.1 and 16% in scenario No.2, the average was calculated from the inhaled viral charges in the three configurations: mask on the emitter, on the receiver, and on both) while the electrostatic and mechanical mask had comparable efficiencies. When the level of leakage was based on the hypothesis of equal pressure drops (No.3 and 4), the FFP2 mask after loss of electrostatic charges was linked to an inhaled viral charge on average 9% (No.3) and 7% (No.4) higher than the FFP2, while the mechanical FFP2 mask caused the inhaled viral charge to be on average 97% (No.3) and 10% (No.4) higher than the FFP2. The significantly higher pressure drop generated by the mechanical FFP2 mask, 240 Pa at 5 cm/s at inhalation and 300 Pa at 8.5 cm/s at exhalation (maximum allowed values according to EN 149) compared to 60 Pa at 5 cm/s (inhalation and exhalation) for the electrostatic version^49^, led to a significantly higher leaking flow for the mechanical FFP2 in scenarios No.3 and 4.

E3 Relative protection of facemasks for speaking, coughing, and sneezing (complementary discussion to Figure 6)

The relative viral charge inhaled by the receiver considering the four expiratory activities is given in **Figure S46**. The protection efficiency rises as the size of the emitted particles and the initial velocity increases. The protection efficiency provided by the face-shield highlights the importance of stopping incoming droplets in reducing the exposure level.

| ****  **a.** |
| --- |
| ****  **b.** |
| ****  **c.** |
| **Figure S46**: Relative protection efficiency of different types of masks and a face-shield for the four expiratory activities. The receiver was located 0.25 m away from the emitter and on the trajectory of the emitted droplets. Scenario No.2 was considered for the calculation of the leaking fraction. The masks were worn by the emitter (a), the receiver (b), and both individuals (c). |

**b.**

E4 Detailed data for the comparison between the exposure level without leakage and with leakage according to the standards (comparison of data in figures 5 and 6)

The introduction of a leakage causes a significant increase of the exposure level. The data shown in Figure 6d indicates a 6.08x10^4^ times increase of the exposure level for a FFP2 mask used as source control between the no-leakage condition and the leakage according to the EN 149 standard (5% leaking flow). The data used to calculate this ratio is given here.

The calculation of the viral charge released from the mask is based on the fraction of the particles penetrating the mask and the fraction released through the leakage. The considered FFP2 mask has a minimum filtration efficiency of 94% at 250 nm, and the efficiency sharply increases for larger particles. The leakage is set to 5% of the emitted flow and the cut-off size, defined by the velocity at exhalation, is calculated at 20 μm. The number-based released fraction is given in **Figure S47**. The corresponding exposure level deposited in the receiver’s lungs is given in **Figure S48**.

| **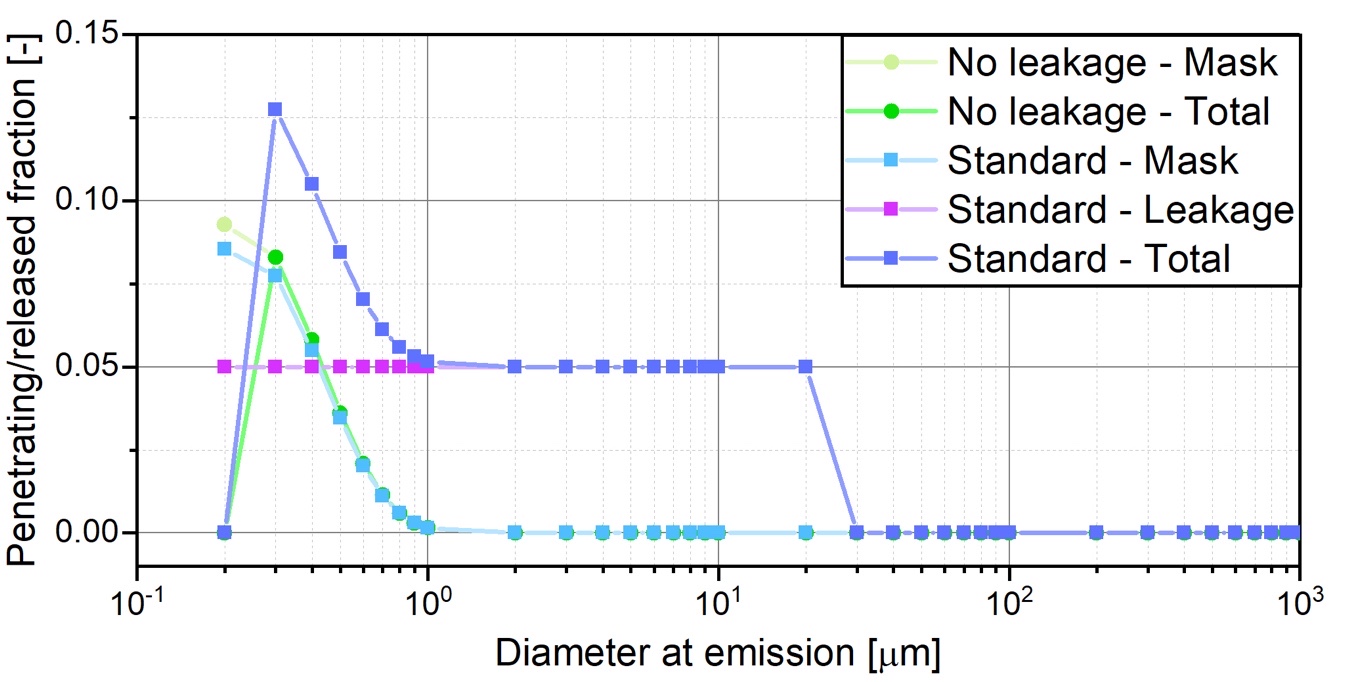** |
| --- |
| **Figure S47:** Comparison of the fraction released from the emitter’s mask considering no leakage and leakage according to the EN 149 standard for FFP2 mask. The calculation refers to the number-based fraction without considering the reach-rate, lung deposition or viral charge accumulation. |

| **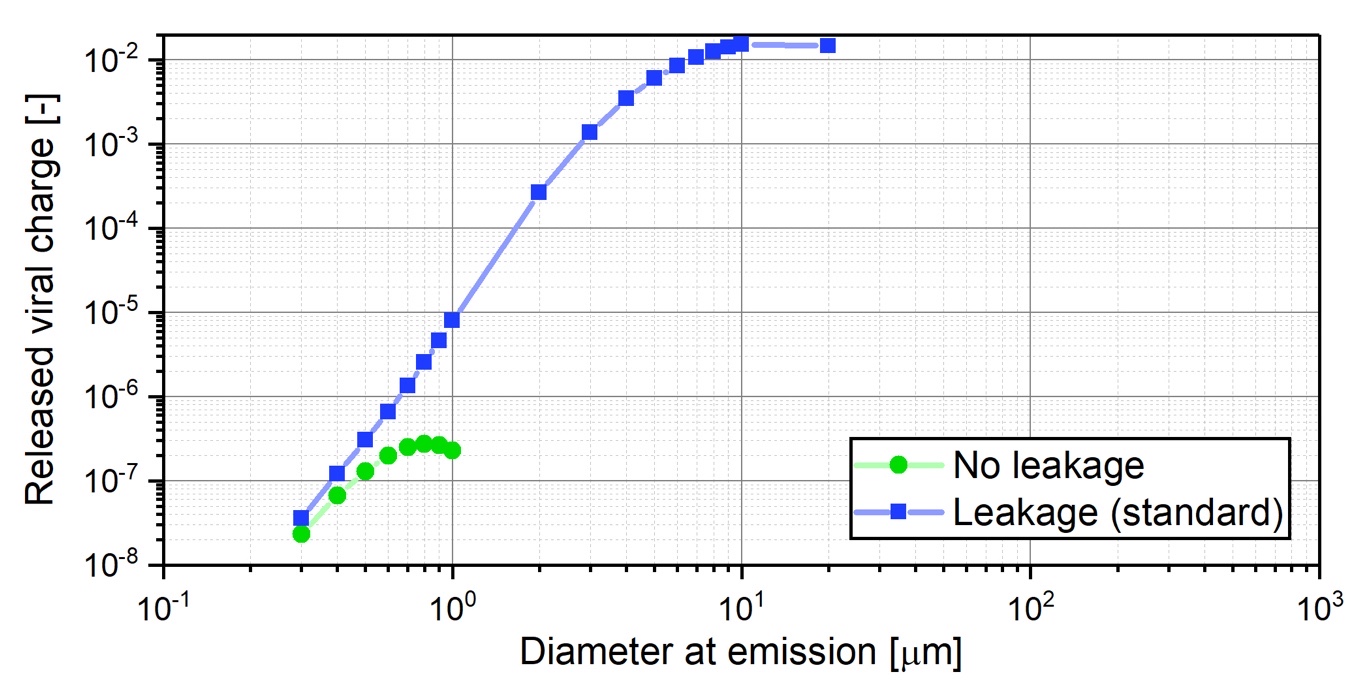** |
| --- |
| **Figure S48:** Comparison of the viral charge inhaled by the receiver considering no leakage and a leakage according to the EN 149 standard for FFP2 masks. Data is not normalized. |

The number-based release shown in **Figure S47** shows that in both situations, the fractions of the particles penetrating the mask are similar. In the situation including the leakage, 95% of the flow passes through the mask compared to 100% in the absence of leakage, explaining the differences. Adding the leaking fraction of 5% increases the penetrating fraction between 200 nm and 20 μm. The conversion into the viral charge inhaled by the emitter (taking into account the emission distribution, lung deposition, reach-rate, and accumulation) shown in **Figure S48** explains the significant difference between the two situations. Between 200 nm and 1 μm, the no leakage situation generates an exposure level of 1.43x10^-6^ viral copies while with the addition of the leakage, it increases to 1.76x10^-5^ viral copies (about a 10-fold increase). Larger particles, between 1 and 20 μm, are released exclusively by the leakage and represent an additional exposure of 8.7x10^-2^ viral copies, leading to a ratio between both scenarios of 6.08x10^4^.

E5. Comparison of the reach-rate and mask filtration efficiency of a FFP2 mask considering leakages of 0% and 29% (Comparison Figures S38c and 6e)

The exposure from breathing without a mask was taken as the reference and the data can be compared to **Figure S38c** (without leakage). The relative importance of the near-field exposure level was significantly higher in **Figure 6e** compared to **Figure S38c** in the cases where only the receiver wore a mask, and dominated the far-field, as the inward leaking flow carried significantly larger particles than the mask flow. The near-field exposure dropped to zero when the mask was worn by the emitter and by both individuals, while in **Figure S38c** it contributed for coughing. The leaking flow led to a reduction of the mask’s face velocity compared to the no-leakage hypothesis, as a smaller fraction of the flow moved through the mask. Consequently, the particles’ reach-rate was reduced, preventing the particles penetrating the emitter’s mask from reaching the receiver.

| **Diameter [μm]** | **FFP2 - leak 0% - Face vel. 0.468 m/s** | | | **FFP2 - leak 29% - Face vel. 0.332 m/s** | | |
| --- | --- | --- | --- | --- | --- | --- |
|  | **Emitter’s mask penetration [-]** | **Leak penetration [-]** | **Reach-rate [-]** | **Emitter’s mask penetration [-]** | **Leak penetration [-]** | **Reach-rate [-]** |
| 2.00E-07 | 0.2192973 | 0 | 0.12540193 | 0.11853747 | 0.29 | 0.00436681 |
| 3.00E-07 | 0.12712361 | 0 | 0.11254019 | 0.06403011 | 0.29 | 0 |
| 4.00E-07 | 0.0729702 | 0 | 0.09967846 | 0.03470528 | 0.29 | 0 |
| 5.00E-07 | 0.04188018 | 0 | 0.07395498 | 0.01905875 | 0.29 | 0 |
| 6.00E-07 | 0.02409437 | 0 | 0.08360129 | 0.01062254 | 0.29 | 0 |
| 7.00E-07 | 0.01389665 | 0 | 0.09324759 | 0.00600329 | 0.29 | 0 |
| 8.00E-07 | 0.00802816 | 0 | 0.09967846 | 0.00343347 | 2.90E-01 | 0 |
| 9.00E-07 | 0.00463993 | 0 | 0.09003215 | 0.00198288 | 0.29 | 0 |
| 1.00E-06 | 0.00267954 | 0 | 0.08360129 | 0.00115381 | 0.29 | 0 |
| 2.00E-06 | 9.85E-06 | 0 | 0.09324759 | 6.31E-06 | 0.29 | 0 |
| 3.00E-06 | 0 | 0 | 0.08038585 | 0 | 0.29 | 0 |
| 4.00E-06 | 0 | 0 | 0.10289389 | 0 | 0.29 | 0 |
| 5.00E-06 | 0 | 0 | 0.09646302 | 0 | 0.29 | 0 |
| 6.00E-06 | 0 | 0 | 0.08038585 | 0 | 0.29 | 0 |
| 7.00E-06 | 0 | 0 | 0.07717042 | 0 | 0.29 | 0 |
| 8.00E-06 | 0 | 0 | 0.06752412 | 0 | 0.29 | 0 |
| 9.00E-06 | 0 | 0 | 0.08038585 | 0 | 0 | 0 |
| 1.00E-05 | 0 | 0 | 0.10610932 | 0 | 0 | 0 |
| 2.00E-05 | 0 | 0 | 0.07395498 | 0 | 0 | 0 |
| 3.00E-05 | 0 | 0 | 0.07073955 | 0 | 0 | 0 |
| 4.00E-05 | 0 | 0 | 0.03215434 | 0 | 0 | 0 |
| 5.00E-05 | 0 | 0 | 0.01286174 | 0 | 0 | 0 |
| 6.00E-05 | 0 | 0 | 0.00321543 | 0 | 0 | 0 |
| 7.00E-05 | 0 | 0 | 0 | 0 | 0 | 0 |
| 8.00E-05 | 0 | 0 | 0 | 0 | 0 | 0 |
| 9.00E-05 | 0 | 0 | 0 | 0 | 0 | 0 |
| 0.0001 | 0 | 0 | 0 | 0 | 0 | 0 |
| 0.0002 | 0 | 0 | 0 | 0 | 0 | 0 |
| 0.0003 | 0 | 0 | 0 | 0 | 0 | 0 |
| 0.0004 | 0 | 0 | 0 | 0 | 0 | 0 |
| 0.0005 | 0 | 0 | 0 | 0 | 0 | 0 |
| 0.0006 | 0 | 0 | 0 | 0 | 0 | 0 |
| 0.0007 | 0 | 0 | 0 | 0 | 0 | 0 |
| 0.0008 | 0 | 0 | 0 | 0 | 0 | 0 |
| 0.0009 | 0 | 0 | 0 | 0 | 0 | 0 |
| 0.001 | 0 | 0 | 0 | 0 | 0 | 0 |

|  |
| --- |
| E6 Detailed data on the filtration of charged filters as a function of the face velocity  The data in Figure 4 show a different response to increased initial velocity for FFP masks relying on electrostatic filtration. The dependance of the electrostatic filtration to the face velocity is shown in **Figure S49** considering the initial velocities for breathing, speaking, coughing, and sneezing. The data in **Figure S50** shows a comparison between the FFP2 mask (electrostatic and mechanical filtration) and the mechanical FFP2.  ****  **Figure S49**: Electrostatic filtration efficiency as a function of the initial flow velocity. Breathing corresponds to 0.064 m/s face velocity, speaking to 0.156 m/s, coughing to 0.468 m/s, and sneezing to 0.8 m/s.  ****  **Figure S50**: Evolution of the filtration efficiency of the FFP2 mask based on electrostatic and mechanical filtration mechanisms (FFP2-E) and the FFP2 mask based only on mechanical filtration (FFP2-M) |

1. References

[1] ƒ, L., Wei, J., Li, Y. & Ooi, A., ﻿Evaporation and dispersion of respiratory droplets from coughing. ﻿*Indoor Air*, **27,** 179–190 (2017). 10.1111/ina.12297

﻿[2] ﻿Wang, J., Chen, D. R. & Pui, D. Y. H., ﻿Modeling of filtration efficiency of nanoparticles in standard filter media. ﻿*Journal of Nanoparticle Research*, **9,** 109-115 (2007). 10.1007/s11051-006-9155-9

[3] Wang, J., Tronville, P., ﻿Toward standardized test methods to determine the effectiveness of filtration media against airborne nanoparticles. ﻿*J Nanopart Res*, **16,** 2417 (2014). 10.1007/s11051-014-2417-z

[4] Hinds, W. C., *Aerosol Technology; Properties, Behavior, and Measurement of Airborne Particles*, Second Edition, Wiley-Interscience (1999).

[5] Lathrache, R., Fissan, H. J. & Neumann, S., ﻿Depostion of Submicron Particles on Electrically Charged Fibers. *J. Aerosol Sci.*, **17,** 3, 446-449 (1986). 10.1016/0021-8502(86)90127-8

[6] Chen, S., Wang, J., Bahk, Y. K., Fissan, H. & Pui, D. Y. H., ﻿Carbon Nanotube Penetration Through Fiberglass and Electret Respirator Filter and Nuclepore Filter Media: Experiments and Models. ﻿*Aerosol Science and Technology*, **48,** 997-1008 (2014). 10.1080/02786826.2014.954028

[7] He, W., Guo, Y., Gao, H., Liu, J., Yue, Y. & Wang, J. ﻿Evaluation of Regeneration Processes for Filtering Facepiece Respirators in Terms of the Bacteria Inactivation Efficiency and Influences on Filtration Performance. ﻿*ACS Nano*, **14,** 13161−13171 (2020). 10.1021/acsnano.0c04782

[8] Oberg, T. & Brosseau, L. M., ﻿Surgical mask filter and fit performance. *American Journal of Infection Control*, **36,** 4 (2008). 10.1016/j.ajic.2007.07.008

[9] Drewnick, F., Pikmann, J., Fachinger, F., Moormann, L., Sprang, F. & Borrmann, S., ﻿Aerosol filtration efficiency of household materials for homemade face masks: Influence of material properties, particle size, particle electrical charge, face velocity, and leaks. ﻿*Aerosol Science and Technology*, **55,** 1 (2021). 10.1080/02786826.2020.1817846

[10] Davies, A., Thompson, K., Giri, K., Kafatos, G., Walker, J. & Bennett, A., ﻿Testing the Efficacy of Homemade Masks: Would They Protect in an Influenza Pandemic? ﻿*Disaster Medicine and Public Health Preparedness*, **7,** 4 (2013). 10.1017/dmp.2013.43

[11] Zhao, M., *et al.*, Household Materials Selection for Homemade Cloth Face Coverings and Their Filtration Efficiency Enhancement with Triboelectric Charging. *Nano Lett.*, **20,** 5544-5552 (2020). 10.1021/acs.nanolett.0c02211

[12] Perić, R. & Perić, M., ﻿Analytical and Numerical Investigation of the Airflow in Face Masks used for Protection against COVID-19 Virus – Implications for Mask Design and Usage. ﻿*Journal of Applied Fluid Mechanics*, **13,** 6, 1911-1923 (2020). 10.47176/jafm.13.06.31812

﻿[13] Bruus, H., *﻿Theoretical microfluidics*, First Edition, Oxford University Press (2008). ISBN: 978–0–19–923509–4

﻿[14] Urs Pauli, Stephane Karlen, and Kathrin Summermatter (2014). ﻿The Importance of Fit-Testing Particulate Filtering Facepiece Respirators!, ﻿*Applied Biosafety*, **19**, 4, DOI: 10.1177/153567601401900402

[15] Lawrence, R. B., Duling, M. G., Calvert, C. A. & Coffey, C. C., ﻿Comparison of Performance of Three Different Types of Respiratory Protection Devices. ﻿*Journal of Occupational and Environmental Hygiene*, **3,** 465-474 (2009). 10.1080/15459620600829211

﻿[16] van der Sande, M., Teunis, P. & Sabel, R., ﻿Professional and Home-Made Face Masks Reduce Exposure to Respiratory Infections among the General Population. ﻿*PLoS ONE*, **3,** 7 (2008). 10.1371/journal.pone.0002618

﻿[17] Kim, J., Roberge, R. J., Powell, J. B., Shaffer, R. E., Ylitalo, C. M. & Sebastian, J. M., ﻿Pressure Drop Of Filtering Facepiece Respirators: How Low Should We Go? ﻿*Int J Occup Med Environ Health*, **28,** 1, 71–80 (2015). 10.13075/ijomeh.1896.00153

﻿[18] Steinle, S., Sleeuwenhoek, A., Mueller, W., Horwell, C. J., Apsley, A., Davis, A., Cherrie, J. W., Gale, K. S., ﻿The effectiveness of respiratory protection worn by communities to protect from volcanic ash inhalation. Part II: Total inward leakage tests. *﻿International Journal of Hygiene and Environmental Health*, **221,** 977–984 (2018). 10.1016/j.ijheh.2018.03.011

[19] Grinshpun, S. A., Haruta, H., Eninger, R. M., Reponen, T., McKay, R. T. & Lee, S., ﻿Performance of an N95 Filtering Facepiece Particulate Respirator and a Surgical Mask During Human Breathing: Two Pathways for Particle Penetration. ﻿*Journal of Occupational and Environmental Hygiene*, **6,** 593–603 (2009). ﻿10.1080/15459620903120086

[20] Wang, B., Wu, H. & Wan, X., ﻿Transport and fate of human expiratory droplets—A modeling approach. ﻿*Phys. Fluids*, **32,** 083307 (2020). 10.1063/5.0021280

[21] Wei, J. & Li, Y. ﻿Enhanced spread of expiratory droplets by turbulence in a cough jet. ﻿*Building and Environment*, **93,** 86-96 (2015). 10.1016/j.buildenv.2015.06.018

[22] Cushman-Roisin, B., *Environmental Fluid Mechanics*, John Wiley & Sons, Inc. (2013), Chapter 9

[23] ﻿ Chan, S. N., Lee, K. W. Y. & Lee, J. H. W., ﻿Numerical modelling of horizontal sediment-laden jets. ﻿Environ Fluid Mech, **14,** 173-200 (2014). ﻿10.1007/s10652-013-9287-2

[24] Bocksell, T. L. & Loth, E., Random Walk Models for Particle Diffusion in Free-Shear Flows. *AIAA JOURNAL*, **39,** 6 (2001). 10.2514/2.1421

[25] Zhang, X., Ji, Z., Yue, Y., Liu, H. & Wang, J., Infection Risk Assessment of COVID-19 through Aerosol Transmission: a Case Study of South China Seafood Market. *Environ. Sci. Technol.*, **55,** 7, 4123–4133 (2021). 10.1021/acs.est.0c02895

[26] Lai, A. C. K. & Nazaroff, W. W., ﻿Modeling Indoor Particle Deposition From Turbulent Flow Onto Smooth Surfaces. *﻿J. Aerosol Sci.*, **31,** 4, 463-476 (2000). 10.1016/S0021-8502(99)00536-4

[27] Wang, C. *Inhaled Particles*, First Edition, Academic Press (2005). ISBN 9780080455013

﻿[28] Edwards, D. A., *et al.*, ﻿Exhaled aerosol increases with COVID-19 infection, age, and obesity. ﻿*PNAS*, **118,** 8 (2021). ﻿10.1073/pnas.2021830118

﻿[29] Johnson, G. R. & Morawska, L., ﻿The Mechanism of Breath Aerosol Formation. *﻿Journal of Aerosol Medicine and Pulmonary Drug Delivery*, **22,** 3 (2009). ﻿10.1089/jamp.2008.0720

[30] Leung, N. H. L., *et al.*, ﻿Respiratory virus shedding in exhaled breath and efficacy of face masks. ﻿*Nature Medicine*, **26,** 676-680 (2020). ﻿10.1038/s41591-020-0843-2

﻿[31] Dabisch, P., *et al.*, ﻿The influence of temperature, humidity, and simulated sunlight on the infectivity of SARS-CoV-2 in aerosols. ﻿*Aerosol Science and Technology*, **55,** 2, 142-153 (2021). ﻿10.1080/02786826.2020.1829536

﻿[32] van Doremalen N, *et al.*, Aerosol and Surface Stability of SARS-CoV-2 as Compared with SARS-CoV-1. *The New England Journal of Medicine,* **382,** 1564-1567 (2020). 10.1056/NEJMc2004973

[33] Chu, D. K., Akl, E. A., Duda, S., Solo, K., Yaacoub, S. & Schünemann, H. J., ﻿Physical distancing, face masks, and eye protection to prevent person-to-person transmission of SARS-CoV-2 and COVID-19: a systematic review and meta-analysis. *Lancet*, **395,** 1973-87 (2020). 10.1016/ S0140-6736(20)31142-9

[34] ﻿Han, Z. Y., Weng, W. G. & Huang, Q. Y. ﻿Characterizations of particle size distribution of the droplets exhaled by sneeze. ﻿*J R Soc Interface*, **10,** 20130560 (2013). 10.1098/rsif.2013.0560

[35] ﻿Alsved, M., *et al*., ﻿Exhaled respiratory particles during singing and talking. ﻿*Aerosol Science and Technology*, **54,** 11 (2020). 1245-1248, 10.1080/02786826.2020.1812502

[36] Duguid, J. P., ﻿The size and duration of air-carriage of respiratory droplets and droplet-nuclei. *J Hyg (Lond)*, **44,** 6, 471–479 (1946). 10.1017/s0022172400019288

[37] Loudon, R. G. & Roberts, R. M., Droplet expulsion from the respiratory tract. *Am Rev Respir Dis*, **95,** 3, 435-42 (1967). 10.1164/arrd.1967.95.3.435

[38] ﻿Chao, C. Y. H., *et al.,* ﻿Characterization of expiration air jets and droplet size distributions immediately at the mouth opening. ﻿*Aerosol Science*, **40,** 122 – 133 (2009). ﻿10.1016/j.jaerosci.2008.10.003

[39] Schijven, J., *et al.,* Exposure assessment for airborne transmission of SARS-CoV-2 via breathing, speaking, coughing and sneezing. preprint on *medRxiv* (2020). n10.1101/2020.07.02.20144832

[40] ﻿Asadi, S., Wexler, A. S., Cappa, C. D., Barreda, S., Bouvier, N. M.& Ristenpart, W. D., ﻿Effect of voicing and articulation manner on aerosol particle emission during human speech. *PLoS ONE*, **15,** 1 (2020). 10.1371/journal. pone.0227699

[41] ﻿Morawska, L., ﻿Size distribution and sites of origin of droplets expelled from the human respiratory tract during expiratory activities. ﻿*Aerosol Science*, **40,** 256-269 (2009). ﻿10.1016/j.jaerosci.2008.11.002

[42] ﻿Fabian, P., Brain, B., Houseman, A. E., Gern, J. & Milton, D. K., ﻿Origin of exhaled breath particles from healthy and human rhinovirus-infected subjects. ﻿*Journal of Aerosol Medicine and Pulmonary Drug Delivery*, **24,** 3 (2011). ﻿10.1089/jamp.2010.0815

[43] ﻿Gerone, P. J., Couch, R. B., Keefer, G. V., Douglas, R. G., Derrenbacher, E. B. & Knight, V., ﻿Assessment of experimental and natural viral aerosols. *Bacteriological Reviews*, **30,** 3 (1966). 10.1128/br.30.3.576-588.1966

[44] Joseph H. W. Lee, and Vincent H. Chu, *Turbulent Jets and Plumes*, Kluwer Academic Publishers (2003), Chapter 2

[45] Hugo B. Fischer, E. John List, Robert C.Y. Koh, Jörg Imberger, and Norman H. Brooks, *Mixing in Inland and Coastal Waters*, Academic Press (1979), Chapter 9

[46] Wolfgang Rodi, *Turbulent Buoyant Jets and Plumes*, Pergamon (1982)

[47] Weibel, E.R., ﻿Design of airways and blood vessels considered as branching trees. *The Lung: Scientific Fundations*, Vol.1, 711-720, Raven Press, New-York (1991).

[48] Sachinidou, P., Heuschling C., Schaniel, J. & Wang, J., ﻿Investigation of surface potential discharge mechanism and kinetics in dielectrics exposed to different organic solvents. *Polymer*, **145**, 447-453 (2018). 10.1016/j.polymer.2018.05.023

[49] ﻿He, W., Guo, Y., Gao, H., Liu, J., Yue, Y. & Wang, J., ﻿Evaluation of regeneration processes for filtering facepiece respirators in terms of the bacteria inactivation efficiency and influences on filtration performance. ﻿*ACS Nano*, **14,** 13161−13171 (2020). 10.1021/acsnano.0c04782
